# A variant of guanidine-IV riboswitches exhibits evidence of a distinct ligand specificity

**DOI:** 10.1080/15476286.2022.2160562

**Published:** 2022-12-22

**Authors:** Felina Lenkeit, Iris Eckert, Malte Sinn, Franziskus Hauth, Jörg S. Hartig, Zasha Weinberg

**Affiliations:** a Department of Chemistry and Konstanz Research School Chemical Biology (KoRS-CB), University of Konstanz, Universitätsstraße 10, 78457 Konstanz, Germany; b Bioinformatics Group, Department of Computer Science and Interdisciplinary Centre for Bioinformatics, Leipzig University, Härtelstraße 16-18, 04107 Leipzig, Germany

**Keywords:** Variant riboswitch, guanidine, guanidinium, comparative genomics, metagenome analysis, N-acetyltransferase, GNAT, agmatine, arginine

## Abstract

Riboswitches are regulatory RNAs that specifically bind a small molecule or ion. Like metabolite-binding proteins, riboswitches can evolve new ligand specificities, and some examples of this phenomenon have been validated. As part of work based on comparative genomics to discover novel riboswitches, we encountered a candidate riboswitch with striking similarities to the recently identified guanidine-IV riboswitch. This candidate riboswitch, the Gd4v motif, is predicted in four distinct bacterial phyla, thus almost as widespread as the guanidine-IV riboswitch. Bioinformatic and experimental analysis suggest that the Gd4v motif is a riboswitch that binds a ligand other than guanidine. It is found associated with gene classes that differ from genes regulated by confirmed guanidine riboswitches. In inline-probing assays, we showed that free guanidine binds only weakly to one of the tested sequences of the variant. Further tested compounds did not show binding, attenuation of transcription termination, or activation of a genetic reporter construct. We characterized an N-acetyltransferase frequently associated with the Gd4v motif and compared its substrate preference to an N-acetyltransferase that occurs under control of guanidine-IV riboswitches. The substrates of this Gd4v-motif-associated enzyme did not show activity for Gd4v RNA binding or transcription termination. Hence, the ligand of the candidate riboswitch motif remains unidentified. The variant RNA motif is predominantly found in gut metagenome sequences, hinting at a ligand that is highly relevant in this environment. This finding is a first step to determining the identity of this unknown ligand, and understanding how guanidine-IV-riboswitch-like structures can evolve to bind different ligands.

## Introduction

In 2002, riboswitches were experimentally validated and broadened the understanding of RNA-based metabolic regulation in bacteria [1–4]. They are non-coding regions in the 5′-UTR of mRNA that form defined structures to bind a small molecule or ion as ligand, and thus enable regulation of the downstream gene [5–8]. Pathways controlled by riboswitches include cofactor, amino acid and nucleotide metabolism [9], as well as motility [10], biofilm formation [11] and virulence [12]. Thus, riboswitches respond to changes in environmental or cellular conditions by adapting gene expression [13].

Many currently known riboswitches were first discovered bioinformatically as conserved RNA structures, known as ‘motifs’, whose ligands were determined later. One example of a widespread motif is the *ykkC-yxkD* RNA motif, which was identified in 2004 and often found upstream of genes encoding multidrug efflux pumps and other transporters, urea carboxylases and purine and amino acid metabolism enzymes [14,15]. Later, the transporter classes and urea carboxylases were also found to be associated with the mini-*ykkC* [16] and *ykkC*-III motifs [17], suggesting that they sense the same ligand. Finally, guanidine was revealed as the natural ligand of examples of all three motifs, now known as guanidine-I, -II and -III riboswitches [18–20]. These riboswitch classes are most commonly associated with the genes *sugE* or *emrE*, later demonstrated to encode guanidine exporters, termed Gdx [21]. However, guanidine riboswitches are also associated with other genes whose products enable the carboxylation and subsequent degradation of guanidine. For instance, enzymes associated with guanidine riboswitches that had been predicted to encode urea carboxylases were shown to favour guanidine as substrate [18,22]. These observations led to the hypothesis that guanidine riboswitches are part of a mechanism to overcome toxic concentrations of guanidine by exporting or degrading it. Additionally, it is possible that guanidine is used by bacteria as a nitrogen source, as we have demonstrated recently. For the purpose of nitrogen assimilation, it seems that guanidine can either be carboxylated and subsequently hydrolysed [22,23] or hydrolysed directly by a Ni-dependent guanidine hydrolase [24]. We recently sought to discover novel guanidine-binding riboswitch classes by applying a comparative discovery strategy to regions upstream of Gdx genes, which are known to be frequently regulated by such riboswitches [25]. This work led to the validation of a fourth class of guanidine riboswitches called guanidine-IV riboswitches [25,26] that was also discovered independently by another group using a different approach [26].

Riboswitches generally consist of an aptamer domain and an expression platform [9]. The aptamer domain forms a highly selective binding pocket to sense a small metabolite or ion, resulting in a conformational change that is transformed by the expression platform into a change in gene expression. The aptamer domains are especially highly conserved, ensuring high selectivity for the cognate ligand and providing the ability to discriminate against other metabolites [27,28]. Point mutations of these highly conserved nucleotides in the aptamer can diminish or eliminate binding affinity, proving that they are essential for ligand recognition. However, highly specific, small mutations in the aptamer domain can also result in riboswitch variants, i.e. riboswitches that have lost the ability to bind the former ligand, and instead sense a different metabolite or signalling compound [29]. For proteins, numerous examples of evolutionary changes in ligands specificity are known, but the list of identified aptamer variants is relatively short. Examples of known aptamer variants are homologous riboswitches binding guanidine and adenine [30] and two flavours of related 2′-deoxyguanidine-binding riboswitches [29,31], c-di-GMP aptamers that are homologous to c-AMP-GMP-binding aptamers [32,33] and coenzyme B_12_ aptamers in mutated forms that sense aquocobalamin [34]. Variants of the FMN riboswitch were shown to no longer bind FMN, but rather the FMN precursor riboflavin and the FMN degradation products lumiflavin and lumichrome [35]. Recently, three guanine riboswitch variants were reported that bind xanthine, 2′-deoxyguanosine, and guanine; the latter was considered a variant because it exhibits improved binding specificity compared to the parental guanine riboswitch [36]. In the case of the guanidine-I riboswitch, different variants carrying mutations in the binding pocket that eliminate guanidine binding were identified and later shown to bind other ligands. Variants of guanidine-I riboswitches that bind ppGpp [37] and PRPP [38] have been validated, and an additional variant class binds certain nucleoside diphosphates, and might sense the total amount of these molecules [39]. At least one more variant class likely exists, but its ligand is not yet known [40]. Riboswitch variants typically exhibit mutations at distinct nucleotide positions that are involved in ligand binding and they bind a variety of ligands [29]. The identification of such variants and their characterization can give insight into the binding mechanisms and sensing capabilities of RNA. Within the present work, we identify an apparent variant riboswitch that is very similar in its conserved structural features to the guanidine-IV motif, but differs from the original sequence at positions that are significantly involved in ligand sensing.

## Results

Discovery of a motif resembling the guanidine-IV riboswitch

As part of a larger screen to enumerate all known protein domains, and find novel motifs upstream of genes encoding each domain family in bacteria and archaea (I.E. Z.W., unpublished data), in an improved version of a previous search strategy [41], we discovered a new RNA motif. This motif was initially detected upstream of genes encoding a predicted transaminase domain. We analysed the initial computational prediction as before [25], with repeated homology searches using Infernal [42] and investigation of structure using CMfinder [41,43], R-scape [44] and manual analysis.

**Figure 1.**
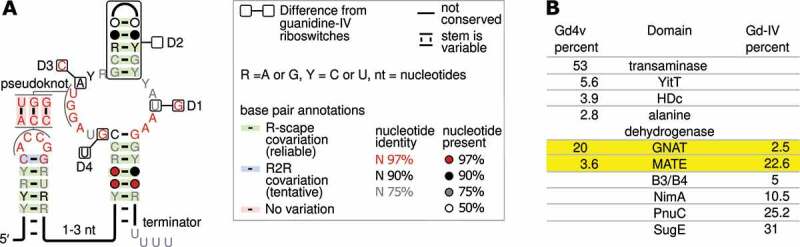
Genetic context and consensus sequence of the Gd4v motif. (A) Consensus features of 395 Gd4v motif RNAs. The inset describes annotations used. ‘Terminator’: this stem appears to be able to function as a Rho-independent terminator. Boxes depict conserved features (named D1-4) of Gd4v RNAs that are incompatible with those of guanidine-IV riboswitches, i.e. that are never or rarely the same. The analogous guanidine-IV riboswitch conservation is shown in the outer boxes. ‘D2’ refers to a hairpin that is missing in guanidine-IV riboswitches. Supplementary Figure 1 depicts the full consensus models, controlling for gene context (see text). (B) The six domains most frequently encoded by genes associated with the 395 Gd4v RNAs or 1,262 guanidine-IV riboswitches. Percentages of RNAs associated with each gene class are given. Yellow rows: domains commonly associated with both RNA motifs. Frequencies add up to less than 100% because of other rarer genes. Conserved domains associated with the listed genes are given in Supplementary Table 1.

We noticed many structural similarities between this motif and the recently published guanidine-IV riboswitch [25,26]. We therefore called this motif the guanidine-IV-variant (Gd4v). Given the similarities, it might be difficult to precisely delineate which RNA sequences belong to the Gd4v motif and which are guanidine-IV riboswitches. Indeed, we noticed 12 sequences in our Gd4v motif alignment that were previously predicted as guanidine-IV riboswitches. Removing these sequences led to an alignment of 395 unique predicted RNAs (Fig. 1A), which are present in the phyla Actinobacteria, Bacteroidetes and Firmicutes. A multiple sequence alignment and depictions of downstream genes are available (Supplementary File 1), as is our alignment in machine-readable Stockholm format (Supplementary File 2). Our impression was that many bacteria containing Gd4v RNAs are associated with human or vertebrate guts. We therefore investigated environmental samples and found a striking apparent enrichment in mammalian guts in comparison to non-gut environments (Supplementary File 3).

Bioinformatic comparison of the new Gd4v motif to guanidine-IV riboswitches

Except for specific differences explained below, the guanidine-IV riboswitch motif and the Gd4v motif are extremely similar in their primary and secondary structure (Fig. 1A). Both motifs consist of two hairpins connected by a pseudoknot, and have many highly conserved nucleotides in and around the pseudoknot. The identities of these highly conserved nucleotides are also very similar in the two motifs. In both motifs, the 3′-most hairpin resembles a Rho-independent terminator, which promotes transcription termination in bacteria [45] and generally consists of a hairpin followed by several U-nucleotides. This terminator appears to play a role in the regulatory mechanism of guanidine-IV riboswitches [25,26]. As both motifs are present in multiple phyla, these similarities in sequence and structure have likely been conserved over a long evolutionary time. The striking similarities thus suggest that the Gd4v motif, like the guanidine-IV riboswitch, consists of riboswitches that use a Rho-independent terminator to upregulate genes in response to their ligand.

Although the similarities could further suggest that Gd4v RNAs also sense guanidine, there are several differences between the motifs that call this assumption into question. We noticed four features (named differences ‘D1’-‘D4’) in which the majority of Gd4v RNAs and the majority of guanidine-IV riboswitches have contradictory patterns of conservation (Fig. 1A). Each of these differences corresponds to properties that are close to 100% conserved in one of the motifs, across multiple phyla, but differ in the other motif. In one case, we previously confirmed that a conserved nucleotide is required for proper binding in guanidine-IV riboswitches [25]. This nucleotide is altered in the Gd4v motif (Fig. 1A, ‘D1’).

Furthermore, the genes regulated by a riboswitch reflect its biological role, and thus its ligand, so riboswitches upstream of dissimilar genes likely bind distinct ligands. Gd4v RNAs are most strongly (over 50%) associated with a gene annotated as transaminase, which is never associated with the guanidine-IV riboswitch (Fig. 1B, Supplementary Table 1), or indeed any known guanidine-binding riboswitch. This transaminase is predicted to be a member of the aspartate aminotransferase (AAT) superfamily of pyridoxal phosphate (PLP)-dependent enzymes, but its precise activity is unknown. On the other hand, the *sugE* gene that is most commonly found downstream of the guanidine-IV riboswitch is never found associated with the variant (Fig. 1B). However, there are also two conserved domains encoded by genes found downstream of both motifs (Fig. 1B): GNAT family N-acetyltransferases, which catalyse the transfer of an acetyl group from acetyl-CoA to a substrate, and multidrug and toxic compound extrusion (MATE)-like proteins. Since the latter family is comprised of transport proteins for which substrate specificities cannot be deduced from the general functional annotation, it is possible or even likely that the guanidine-IV-riboswitch- and Gd4v-associated members of the MATE proteins transport different substrates. (With regard to the associated N-acetyltransferases we characterized both an example of a guanidine-IV riboswitch-associated and a Gd4v-motif-associated enzyme and found that they acetylate similar but different substrates, see below.)

The differences in gene associations suggest that the Gd4v motif senses a ligand other than guanidine, and the structural deviations between the motifs could correspond to binding core changes necessary to alter ligand specificity. However, we wished to confirm that the distinct genes are truly correlated with distinct structural patterns, which would be expected if Gd4v RNAs bind a ligand other than guanidine. The difficulty in drawing a precise boundary between the motifs complicated this analysis somewhat. The domain most commonly encoded by genes predicted to be regulated by the Gd4v motif is PRK07324 [46], which encodes a transaminase. Since this domain is not associated with any known guanidine riboswitch, if any of our predicted Gd4v RNAs do not bind guanidine, the RNAs upstream of PRK07324 genes should certainly not bind guanidine. Similarly, guanidine-I, -II, -III and -IV riboswitches are all commonly upstream of genes encoding COG2076 (presumably guanidine exporters), and COG2076 is not associated with any Gd4v RNA. Therefore, predicted riboswitches upstream of COG2076 genes should bind guanidine. We therefore extracted and compared two RNA subsets: (1) Gd4v RNAs upstream of PRK07324 genes and (2) guanidine-IV riboswitches upstream of COG2076 genes (Supplementary Figure 1). Two features strictly distinguish the two RNA subsets. The D1 position (Fig. 1A, Supplementary Figure 1) is always a G nucleotide in the guanidine-IV riboswitch subset, but never in the Gd4v RNAs. The D2 feature refers to a hairpin on top of the terminator hairpin, which is always present in the Gd4v subset, but never present in the guanidine-IV riboswitch subset. Two additional features, termed D3 and D4, are highly, though not perfectly contrasted (Supplementary Figure 1). Thus, structural changes that could reflect an altered ligand are correlated with distinct gene contexts across multiple phyla. These results are thus consistent with the hypothesis that Gd4v RNAs are riboswitches whose natural ligand is not guanidine.

In-line probing analysis of Gd4v motif RNAs

**Figure 2.**
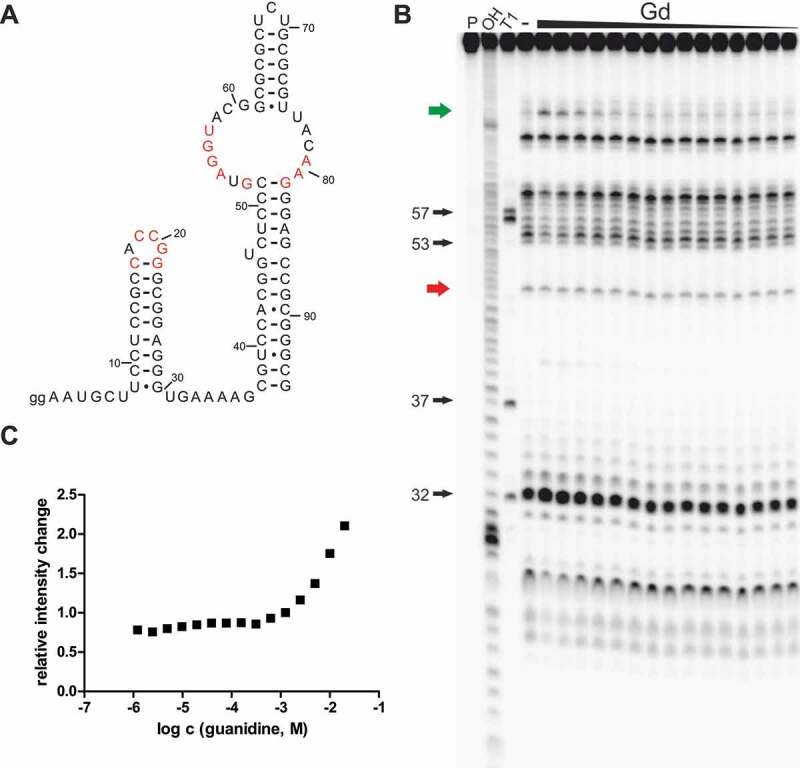
Gd4v motif RNA binds guanidine with low affinity. (A) The predicted structure of *95 Csp* RNA. Red nucleotides have the same meaning as in Fig. 1A. Internucleotide linkages are numbered. (B) PAGE analysis of an in-line probing reaction of 5´ ^32^P-labelled *95 Csp* RNA (sequence from *Cloacibacillus sp*.) without (-) or with guanidine hydrohydrochloride in a range of 0.61 μM – 20 mM. (**C**) Plot of the relative intensity change of modulated RNA cleavage depending on the guanidine hydrochloride concentration. Relative intensity change was determined based on quantification of a modulated band (green arrow in B) relative to the intensity of a constant band (red arrow in B).

To further support the hypothesis that the Gd4v motif senses and binds a ligand other than guanidine, we investigated a 95-nucleotide-long RNA construct (*95 Csp*) (Fig. 2A) from the 5′-UTR of an aminotransferase gene of *Cloacibacillus sp*. in an in-line probing reaction with a large set of compounds (Supplementary Table 2). We found that guanidine causes a concentration-dependent structural modulation to this construct (Fig. 2B) but could not detect any modulation with agmatine or arginine. However, guanidine seems to bind this construct with a very poor affinity compared to the guanidine-IV riboswitch. We quantified the extent of spontaneous cleavage at a modulated position that is located in the bulge opposite to the pseudoknot forming region, but were not able to determine an apparent K_D_ value since no saturation was achieved with concentrations still suitable for the assay (Fig. 2C). However, structural modulation induced by binding of guanidine to the RNA can be observed with concentrations higher than 1 mM. In comparison, the guanidine-IV riboswitch showed changes in modulation pattern at concentrations above ~20 μM. We have also tested constructs of the Gd4v motif found in *Dialister succinatiphilus* (*95 Dsu*) and *Mitsuokella jalaludinii* (*105 Mja*). With these constructs, we did not observe any modulation in response to guanidine, even at high concentrations up to 10 mM.

Although we do see a guanidine-dependent structural modulation with the construct *95 Csp*, the poor affinity indicates that either the chosen construct is not optimal, with a different construct potentially yielding a better affinity, or guanidine is not the natural ligand of the Gd4v motif. It has already been shown for the guanidine-IV riboswitch that the length of the construct tested in in-line probing can be crucial for the observed binding affinity to the ligand [26]. Salvail *et al*. showed that binding to guanidine is significantly reduced when the investigated in-line construct carries the full length of the terminator stem, including the following U residues. Formation of the terminator stem leads to a shorter single-stranded linker between the two stems, which might prevent the helices from forming, thus inhibiting binding. As the guanidine-IV riboswitch and the Gd4v motif are structurally almost identical, they presumably use the same mechanism to bind their cognate ligand. Thus, we have also tested a shorter construct that does not include the full terminator stem sequence (*81 Csp*). The guanidine dose-dependent structural changes observed with *81 Csp* were even weaker compared to the long construct *95 Csp* (Supplementary Figure 2). Thus, changes in the length of the construct do not increase the binding affinity to guanidine, further suggesting that guanidine is not the natural ligand for the Gd4v riboswitch. Many characterized riboswitches, including the guanidine-IV riboswitch, have been shown to bind derivatives of their cognate ligands with a poorer affinity. Thus, it would make sense if the variant binds guanidine with a weak affinity if a guanidine derivative is its natural ligand. Given this hypothesis, we tested several guanidine derivatives, compounds carrying primary amino groups and signalling molecules (Supplementary Table 2). Besides guanidine, only methyl-guanidine and hydroxyl-guanidine led to a structural change of the *95 Csp* construct. However, the affinities of these two guanidine derivatives were even weaker compared to guanidine (data not shown).

**Figure 3.**
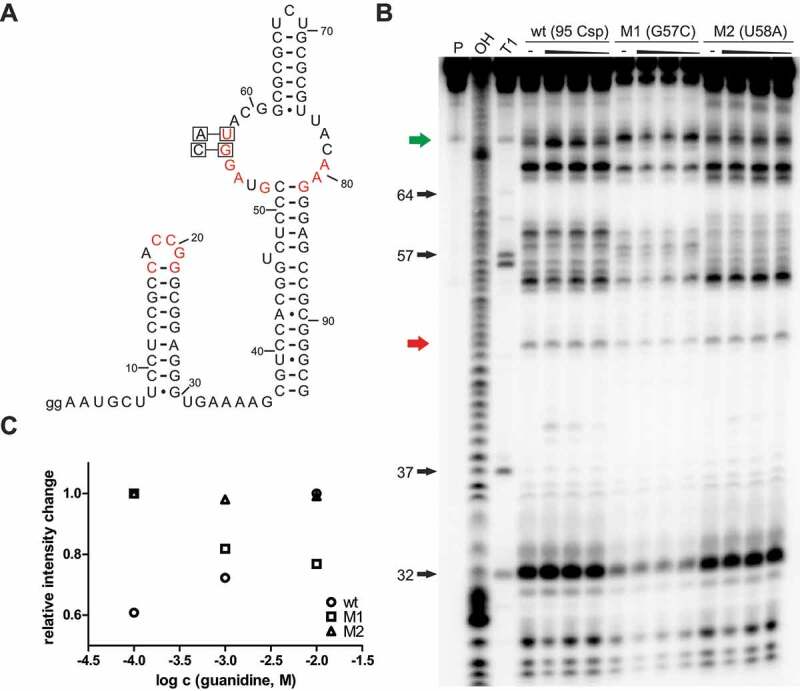
Point mutations of conserved nucleotides compromise guanidine binding to Gd4v motif RNA. (**A**) Sequence and secondary structure of *95 Csp* RNA with the location of point mutations in constructs M1 (G57C) and M2 (U58A). (**B**) PAGE analysis of an in-line probing reaction of 5′ ^32^P-labelled 95 Csp wt RNA and the mutants M1(G57C) and M2(U58A) without (−) or with guanidine hydrochloride with concentrations of 10 mM, 1 mM and 100 μM. P, OH and T1 represent 5′ ^32^P-labelled RNA undergoing no reaction, digest under alkaline conditions, or digest with RNase T1, respectively. (**C**) Plot of the relative intensity change of modulated RNA cleavage depending on the guanidine hydrochloride concentration for the constructs *95 Csp* wt, M1 and M2. Relative intensity change was determined based on quantification of a modulated band (green arrow in (B)) relative to the intensity of a constant band (red arrow in (B)). Relative intensity changes were normalized to the highest value to allow better comparison.

Highly conserved nucleotides riboswitch sequences are often essential for the formation of a binding pocket and directly involved in interactions with the cognate ligand to enable highly selective binding. For many identified riboswitches, point mutations at highly conserved positions in the sequence led to a diminished interaction or fully eliminated it, proving that these nucleotides are involved in binding. This was also shown for the guanidine-IV riboswitch [25]. Guanidine-dependent *in vitro* modulation and *in vivo* gene expression control were fully eliminated by point mutations in the second loop. It seems reasonable to hypothesize that the highly conserved loop regions of stem 1 and stem 2 come together to form a selective binding pocket. We speculated that the Gd4v motif functions as a riboswitch using the same mechanism. Thus, we hypothesized that point mutations in the second loop of the variant would also diminish guanidine-dependent modulation. Two different mutant constructs M1 (C57G) and M2 (U58A) of the *95 Csp* RNA (Fig. 3A) were tested in in-line probing reactions in the absence or presence of guanidine hydrochloride at 10 mM, 1 mM and 100 μM (Fig. 3B). When determining the fraction of RNA bound to guanidine, we observed that the guanidine-dependent modulation effect is extremely diminished, or almost eliminated (Fig. 3C). Thus, these point mutations significantly impair guanidine binding, which implies that the binding of guanidine to the *95 Csp* construct is not due to an unspecific effect in response to high guanidine concentration, but requires highly conserved nucleotides in its primary sequence.

Transcription termination assays

As noted above, the similarity between Gd4v RNAs and guanidine-IV riboswitches suggests that Gd4v uses a Rho-independent terminator to regulate gene expression. Therefore, we used a transcription termination assay to test the potential ligands of this orphan riboswitch. Transcription of a DNA template for a 170-nucleotide-long RNA construct (*170 Csp*) from *Cloacibacillus sp*. and a 162-nucleotide-long RNA construct (*162 Dsu*) from *Dialister succinatiphilus* was monitored. Both constructs carry the Gd4v motif sequence and include a Rho-independent terminator stem followed by 7 U residues, the start codon and 47 (*170 Csp*) and 43 (*162 Dsu*) nucleotides of the open reading frames of the downstream genes. We speculate that the Gd4v acts as a transcriptional ON-switch analogously to the guanidine-IV riboswitch, expecting an increase of the full-length product and a decrease of the termination product in response to the natural ligand. For both constructs used in the transcription termination assay, we observed only the formation of the presumed termination product and could not detect the full-length product in the absence of potential ligands. This finding supports our hypothesis that the Gd4v appears to be an ON-switch. Utilizing this assay, we tested all compounds listed in Supplementary Table 2 on both constructs, *162 Dsu* and *170 Csp*. For the construct *162 Dsu* we did not observe changes in the transcription product ratio in response to any compound tested. For the construct *170 Csp*, we observed a decrease of the termination product in a concentration-dependent manner in response to guanidine hydrochloride. However, we did not detect a full-length transcription product (Supplementary Figure 3). Hence, it seems likely that guanidine is not the natural ligand to this motif or that we did not find the right conditions for this construct. This again supports our hypothesis that the Gd4v motif is a riboswitch whose natural ligand might be structurally related to guanidine. Thus, guanidine is still able to bind the variant motif with a low affinity but does not trigger the change of transcription product formation in the way it is expected to with the natural ligand itself.

*In vivo* analysis of gene regulation

To investigate the effects of the Gd4v motif on gene expression, we monitored its control over a reporter gene *in vivo*. We used a reporter plasmid carrying the Gd4v motif of *Cloacibacillus sp*. in the 5′-UTR of an *egfp* reporter gene. The same reporter plasmid, only varying in the motif sequence, has already been studied by us to characterize the guanidine-IV riboswitch. We showed that the guanidine-IV riboswitch exerts very pronounced control over the expression of the reporter gene in response to guanidine. Given our hypothesis that the Gd4v motif and guanidine-IV riboswitch use the same mechanism, we expect that the variant motif also functions correctly within the context of this plasmid. We transformed the Gd4v reporter plasmid in *S. aureus* and monitored the expression of *egfp* by measuring the fluorescence intensity and normalizing it by the optical density (OD_600_). When cultivating the strain in rich medium (BHI medium), fluorescence was detected only slightly above background level of untransformed *S. aureus* cultures. We conclude that the Gd4v motif essentially turns off expression completely in this case. Assuming the variant to be an ON-switch, these *in vivo* results indicate that the natural ligand is not present in high concentrations in *S. aureus* growing under these conditions. When the *S. aureus* strain was cultivated in the presence of 5 mM guanidine hydrochloride, no change in fluorescence intensity was observed (Supplementary Figure 4). Although a change in the modulation pattern in in-line probing reactions was detected for the Gd4v motif in response to guanidine addition, the binding affinity is very low. *In vivo*, it was not possible to use guanidine hydrochloride concentrations higher than 5 mM in the medium without affecting bacterial growth. It is possible that, with this amount of guanidine hydrochloride in the medium, the intracellular concentration is not high enough to induce a detectable change in gene expression [47]. However, the lack of induction of gene expression further suggests that guanidine is not the natural ligand.

Gd4v- and guanidine-riboswitch-associated genes encode acetyltransferases with different specificities

**Figure 4.**
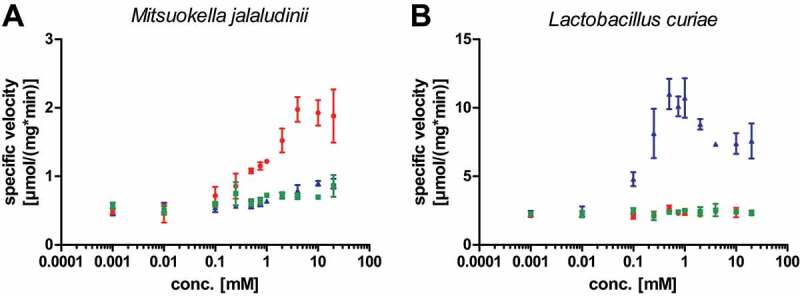
**Characterization of a Gd4v-motif- and guanidine-IV-riboswitch-associated N-acetyltransferases from the GNAT family**. (A): Gd4v-controlled GNAT from *Mitsuokella jalaludinii*: Agmatine (red circles) and to a lesser extent arginine (green squares) and canavanine (blue triangles) were found to be acetylated by the enzyme in a substrate screen (Supplementary Table 4). (B): A GNAT under control of a guanidine-IV riboswitch from *Lactobacillus curiae* only showed activity only towards canavanine. Data points are the mean of technical triplicates (n = 3; s.d.).

While there is a clear difference between genes associated with Gd4v RNAs and guanidine-IV riboswitches, both motifs appear to regulate genes encoding Gcn5-related N-acetyltransferase (GNAT) enzymes (Fig. 1B). If Gd4v RNAs bind a ligand other than guanidine, it is likely that they regulate GNAT proteins with a distinct substrate specificity that fits into the biochemistry of the Gd4v ligand. Moreover, knowledge of the N-acetyltransferase substrate could shed light on the Gd4v motif’s ligand. We recombinantly expressed and purified a representative GNAT found under control of the Gd4v candidate motif from the bacterium *Mitsuokella jalaludinii* and a second GNAT found controlled by a guanidine-IV riboswitch from *Lactobacillus curiae*. We tested a set of primary amines including guanidines (Supplementary Table 3) as possible substrates for the GNATs in acetylation assays utilizing acetyl-coenzyme A as co-substrate. The Gd4v-associated GNAT was found to acetylate agmatine and to a lesser extent arginine and canavanine (δ-oxa-arginine) (Fig. 4A). In contrast, the guanidine-IV-riboswitch-associated GNAT from *L. curiae* acetylates canavanine but discriminates strongly against arginine and agmatine (Fig. 4B). Hence, the GNATs associated with guanidine-IV riboswitches and the Gd4v motif seem to have different but somewhat similar substrate specificities. However, all three compounds identified as substrates of the Gd4v motif-associated GNAT enzyme were ruled out as ligands of the Gd4v RNA via in-line probing and transcription termination assay, as described above. The finding that an enzyme under the control of a guanidine riboswitch acts on canavanine is interesting, since we recently showed that other guanidine riboswitch-controlled activities are also concerned with canavanine (Hauth et al, manuscript submitted), hinting at a so-far underappreciated connection between guanidine and canavanine metabolisms.

## Discussion

Via a bioinformatics strategy based on inspecting homologous genes for conserved RNA structures, we identified a variant of the guanidine-IV riboswitch class. This guanidine-IV variant (Gd4v) motif is highly similar to the guanidine-IV riboswitch in its primary and secondary structure. However, the two motifs exhibit conserved structural and sequence differences at positions involved in ligand binding. Moreover, the associated genes are distinct: e.g. the Gd4v motif is never associated with *sugE* genes, although they are the most common gene found upstream of the guanidine-IV riboswitch and are highly associated with all known guanidine riboswitch classes. Additionally, the most frequently regulated gene class for the Gd4v motif, encoding a transaminase, is not known to be regulated by any known guanidine riboswitch class. Nevertheless, both motifs also show an overlap, as they both appear to regulate genes encoding MATE-like transporters and N-acetyltransferases (GNATs). However, it is possible or even likely that the transporters have different substrates. In addition, we have shown that the GNATs associated with the guanidine-IV riboswitch and the Gd4v motif display different substrate specificities.

Based on motifs’ similarities and differences in their sequence and genetic surroundings, we hypothesized that the Gd4v motif is homologous to guanidine-IV riboswitches, that it likely binds a different ligand and that it acts via the same mechanism to control downstream gene expression. In binding assays, we showed that the Gd4v motif undergoes structural modulation in response to guanidine. However, the affinity is very weak, further suggesting that guanidine is not its natural ligand, but could be structurally related to it. Despite the poor affinity, we demonstrated that the binding is indeed specific and requires the presence of some highly conserved nucleotides. In transcription termination assays, as well as in an *in vivo* reporter system, no detectable effect in response to guanidine or a guanidine analogue was observed. Our results on the Gd4v motif support our initial hypothesis that the motif is a riboswitch, acting as a transcriptional ON-switch that activates gene expression in response to a guanidine derivative.

Assuming that Gd4v motif RNAs are homologs of guanidine-IV riboswitches, a change of specificity between guanidine and the Gd4v motif’s unknown ligand has occurred at least once. The Gd4v motif and guanidine-IV riboswitches are both similarly widespread and numerous, so there is no reason to favour either riboswitch as the more likely original ancestor. It is additionally possible that specificity changes occurred multiple times. Both the Gd4v motif and guanidine-IV riboswitch are present in many – but far from all – species in multiple phyla. This pattern could suggest that the motifs have disappeared from multiple lineages. Alternately, these RNAs might frequently be horizontally transferred. The enrichment of Gd4v motif RNAs in mammalian guts would provide an environment in which such horizontal transfers could easily occur between organisms that presumably have a need to regulate genes based on the Gd4v motif’s unknown ligand.

The genes controlled by the Gd4v riboswitch candidate fall into rather diverse classes (Fig. 1B) but allow some speculation about the chemical features of the ligand. For example, the most abundant class regulated are PLP-dependent enzymes such as transaminases. Hence, it is likely that the ligand of the Gd4v motif is a primary amine, since this functionality is necessary in order to serve as substrate for PLP-dependent transaminase-like enzymes. As noted above, the similarity of the Gd4v motif to the guanidine-IV riboswitch suggests that it is also upregulated in response to its ligand, so it would be logical if the ligand can be processed by the PLP-dependent enzymes, although the ligand and this enzyme might be more distant from each other in the relevant biochemical pathway. In addition, the PLP-dependent enzymes can be found in a common operon that encodes enzymes annotated as ornithine cyclodeaminase, alanine dehydrogenase or mu crystalline family (COG2423), see Supplementary File 1. These enzymes catalyse redox reactions interconverting amines and imines, often acting on intramolecular imines that can form from transamination reactions of diamines and their subsequent cyclization to metabolites such as pyrrolines and piperidines. These compounds are frequently found as intermediates in the metabolism of amino acids such as ornithine, lysine, and proline.

The second most abundant enzymatic activity controlled by the Gd4v riboswitch candidate is an N-acetyltransferase, also suggesting that the ligand contains a primary amine. We have shown that a Gd4v-associated enzyme preferentially acetylates agmatine. However, both compounds did not show activity in both in-line probing and transcription termination assays. Nevertheless, the evidence gathered so far is in accordance with a nitrogen-rich compound, a hypothesis that would also fit the observation that the Gd4v motif is found strikingly prevalent in organisms that live in mammalian guts, whereas the motif seems to be far less frequently found in metagenome sequences that are obtained from soil or plant habitats. The apparent enrichment of the Gd4v motif in mammalian guts suggests that the ligand is prevalent in this environment. Further research is necessary in order to identify the nature of the ligand of the Gd4v riboswitch candidate.

## Methods and Materials

### Bioinformatics

We used the same genome and metagenome sequences and annotation as previously [25]. As before, the final alignment was depicted using R2R [48] based on covariation annotations of R-scape with the -s flag. Conserved features of guanidine-IV riboswitches were analysed using R2R and a previously published alignment [25].

### Oligonucleotides and chemicals

Oligonucleotides and radioactively labelled nucleotides ([γ-^32^P]- and [α-^32^PP]-ATP) were purchased from Sigma-Aldrich and Hartmann Analytic, respectively. Guanidine hydrochloride, urea and arginine were purchased from Roth and other compounds (Supplementary Table 2) were purchased from Acros Organics.

### RNA oligonucleotide preparation

Synthesis of DNA templates and subsequent RNA oligonucleotide labelling were performed as described previously [25]. DNA templates contained a T7-promotor to enable *in vitro* transcription using T7 RNA polymerase (NEB). DNA templates used for this study are listed in Supplementary Table 4. Purified RNA was then dephosphorylated with Shrimp Alkaline Phosphatase (NEB) and its 5′ terminus was labelled using t4 polynucleotide kinase (NEB) and 20 µCi [γ-^32^P]-ATP. Purified and [γ-^32^P]-labelled RNA was precipitated and dissolved in water to a concentration of 1 kBq/µl.

### In-line probing reaction

In-line probing reactions were conducted as described previously [49,50]. For each reaction, 1 kBq of labelled RNA was incubated in the presence or absence of the potential ligand with an in-line probing buffer (20 mM MgCl2, 100 mM KCl and 50 mM Tris HCl (pH 8.3 at 23°C) for ~48 h. Reactions subjected to 10% PAGE were viewed with a phosphorimager (GE Healthcare Life Sciences). Quantification of band intensities and calculation of fraction bound values was performed as described previously [25]. With guanidine, saturation was not achieved with concentrations still suitable for the assay. Thus, band intensity changes were normalized to the highest concentration possible.

### Cloning and overexpression of Gd4v-associated GNAT

The gene fragment encoding an N-acetyltransferase from *Mitsuokella jalaludinii* was synthesized by GeneArt^TM^ (ThermoFisher). The fragment was codon optimized for expression in *E. coli*. The gene fragment was inserted into the pET28a vector by restriction enzyme cloning to obtain an N-terminal His-tagged version of the N-acetyltransferase.

For recombinant expression of the plasmid was transformed into *E. coli* BL21(λDE3) gold (Invitrogen) cells. Cells were grown at 37°C to an OD_600_ of 0.5, transferred to 18°C and induced overnight with 0.5 mM IPTG. Cells were harvested by centrifugation, resuspended in lysis buffer (20 mM TrisHCl, 20 mM Imidazole, 200 mM NaCl, pH 7.5, cOmplete^TM^ protease inhibitor (Roche)), lysed by ultrasonication (Branson). After centrifugation at14500rpm at 4°C the supernatant was filtered through 0.22 µM filters and loaded onto a gravity flow column containing Ni-NTA agarose. After several wash steps with lysis buffer, the protein was eluted with 500 mM imidazole. Then, the His-Tag was cleaved by TEV protease, followed again by Ni-NTA chromatography. The protein was buffer exchanged into 20 mM TrisHCl, 200 mM NaCl, pH 7.5 using PD10 desalting columns (Cytiva) and concentrated using Amicon^TM^ ultracentrifugal filters (Merck). Protein purity was analysed by SDS-Page gels.

### Acetylation assay

For the acetylation assay Ellman’s reagent (5,5′-dithiobis-(2-nitrobenzoic acid) or DTNB) was used. 5 µl of the respective substrate were mixed with 20 µl reaction mix (20 mM TrisHCl, 200 mM NaCl, pH 7.5, 0.5 mM Acetyl-CoA, 500 nM Mja GNAT) in a 96 well plate. After incubation for 15 minutes at room temperature 75 µl stop buffer (100 mM TrisHCl pH 8 M Urea, pH 8.0) were added, followed by the addition of 100 µl of DTNB reagent (100 mM TrisHCl, pH 8.0, 2 mM DTNB, 1 mM EDTA). After 5 min of incubation at room temperature the absorbance at 420 nm was measured. For quantification different concentrations of CoA were used to obtain a calibration curve.

### Transcription termination assay

Transcription termination assay was performed as previously described [25]. DNA templates contained the T5 promotor sequence, motif sequence and parts of the downstream sequence (~50 nt). After amplification and purification, 10 ng/μl of DNA template, 1.8 mM NTPs, 2 μCi [α-^32^P]-ATP, *E. coli* RNA polymerase and the potential ligand were incubated for 8 min at 37°C. For analysis and visualization, a 10% PAGE a phosphorimager (GE Healthcare Life Sciences) was used. Band intensities were quantified using ImageQuant.

### Genetic reporter assays

The plasmid pCN-Pblaz-GFP was used for reporter assays, and was kindly provided by the Romby Group (University of Strasbourg, Strasbourg, France). Methods for transformation into *Staphylococcus aureus* (*S. aureus*) RN4220 and its cultivation conditions were chosen as described previously [25]. GFP analysis was conducted with excitation and emission wavelengths of 488 nm and 535 nm, respectively, with normalization of fluorescence to OD_600_. A Tecan plate reader was used to measure both OD_600_ and GFP fluorescence.

## Supplementary Material

Supplemental Material

## Data Availability

The authors confirm that the data supporting the findings of this study are available within the article and its supplementary materials. Additionally, alignments from the Weinberg group in papers accepted for publication are available in the ZWD repository (https://bitbucket.org/zashaw/zashaweinbergdata/src/master).
