# Supplementary material for: A variant of guanidine-IV riboswitches exhibits evidence of a distinct ligand specificity
Source: RNA Biol. 2022 Dec 22;20(1):10–9. doi: 10.1080/15476286.2022.2160562 (PMC9788692; doi:10.1080/15476286.2022.2160562)
Supplement: Supplemental Material [file KRNB_A_2160562_SM7488.zip › 220926_Gd4var_supplementary.docx]

**Supplementary materials for:**

**A variant of guanidine-IV riboswitches exhibits evidence of a distinct ligand specificity**

**Felina Lenkeit^1^, Iris Eckert^2^, Malte Sinn^1^, Franziskus Hauth^1^, Jörg S. Hartig^1*^, and Zasha Weinberg^2*^**

1: Department of Chemistry and Konstanz Research School Chemical Biology (KoRS-CB), University of Konstanz, Universitätsstraße 10, 78467 Konstanz, Germany

2: Bioinformatics Group, Department of Computer Science and Interdisciplinary Centre for Bioinformatics, Leipzig University, Härtelstraße 16-18, 04107 Leipzig, Germany

* Correspondence should be addressed to [joerg.hartig@uni-konstanz.de](mailto:joerg.hartig@uni-konstanz.de) or zasha.weinberg@gmail.com

**Descriptions of supplementary files**

**Supplementary File 1**: Data on Gd4v motif RNAs in printable PDF format. The taxonomy information and downstream genes of all predicted Gd4v motif RNAs are given. A multiple-sequence alignment is also included.

**Supplementary File 2**: A machine-readable multiple-sequence alignment of the Gd4v motif, in Stockholm format.

**Supplementary File 3**: A spreadsheet showing the frequency of Gd4v motif RNAs in sequence samples from different environments.

**Supplementary figures**


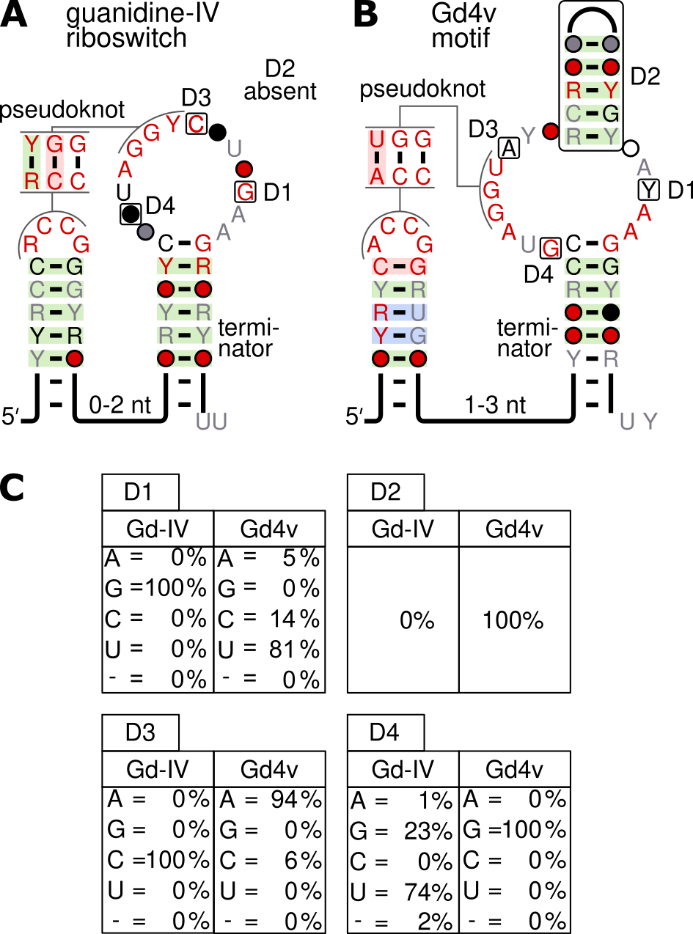


**Supplementary Figure 1: comparison of guanidine-IV riboswitches and Gd4v RNAs using gene controls.** (**A**) Consensus diagram of guanidine-IV riboswitches that are upstream of genes encoding domain COG2076, which presumably encode guanidine exporters ^1^. Due to the gene association, we expect that all considered riboswitches are indeed specific to guanidine. Due to the restricted gene context, the conservation levels differ in some cases from those in Figure 1. In particular, the frequency of U nucleotides at position D4 (see part C) is slightly under the threshold to depict it with the letter U. Annotations have the same meaning as in Figure 1. (**B**) Consensus diagram of Gd4v RNAs that are upstream of genes encoding PRK07324, which are predicted transaminases. This gene association likely eliminates guanidine-IV riboswitches from consideration, but also changes conservation levels from those of Figure 1. Annotations have the same meaning as in Figure 1. (**C**) Numerical conservation levels of the differing conserved features D1-D4. “Gd-IV”: conservation in guanidine-IV riboswitches. “Gd4v”: conservation in Gd4v RNAs. For D1, D3 and D4, the frequencies of the four nucleotides and the gap (-) are shown. Since D2 refers to a hairpin, and not any specific nucleotide position, the numbers depict the percentage of motif examples with a hairpin.


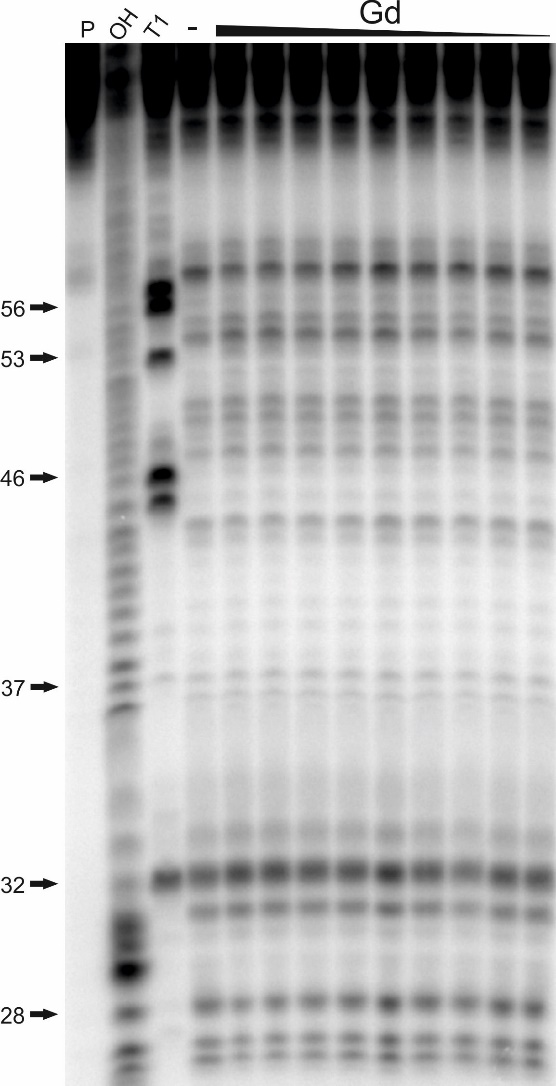


**Supplementary Figure 2: shorter construct of Gd4v motif RNA binds guanidine with poorer affinity than the longer construct.**  PAGE analysis of an in-line probing reaction of 5´ 32P-labeled *81 Csp* RNA (sequence from *Cloabacillus sp.*) without (-) or with guanidine hydrochloride in a range of 39 μM – 10 mM. A quantitative analysis of positions 54 and 58 reveal very weak dose-dependent modulation (data not shown), even though the change is not visually obvious.


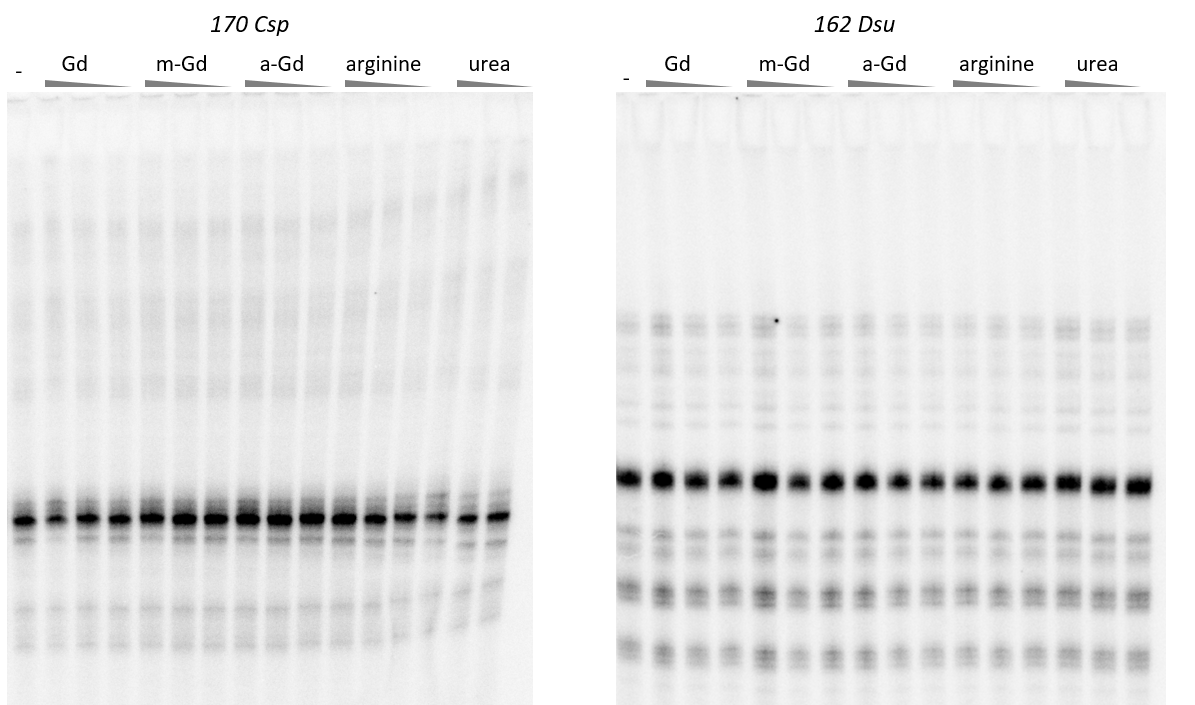


**Supplementary Figure 3: TTA of *170 Csp* and *162 Dsu*.**  PAGE analysis of TTA internally labeled with ^32^P. *170 Csp* from *Cloacibacillus sp.* and *162 Dsu* from *Dialister succinatiphilus*. Assays were supplemented with 10 mM. 1 mM and 100 µM of the designated compound. A control reaction without compound was loaded aside (-). Prominent bands corresponds to the size of the expected termination product.


**Supplementary Figure 4: *In-vivo* reporter assay of the Gd4v construct in comparison with the previously described Guanidine-4 riboswitch construct ^1^.**  The Gd4v motif from *Cloacibacillus sp.* was inserted in the 5’-UTR of *egfp* on a reporter plasmid for expression in *S. aureus* as described previously ^1^. Reporter strains were grown in full medium supplemented with 5 mM guanidine hydrochloride. Fluorescence was measured and normalized to OD_600_. A reporter construct with constitutively expressed *egfp* was used as control. Error bars represent the standard deviation from three independent experiments.

**Supplementary tables**

Supplementary Table 1: Domain accessions used. The domain accessions from the Conserved Domain Database version 3.16 for the categories shown in Figure 1B are listed.

| **Name** | **Accessions** |
| --- | --- |
| Transaminase | cd00609, COG0436, PLN00175, PRK07324, TIGR02371, TIGR03947 |
| YitT | pfam02588, cd16380, COG1284 |
| HDc | smart00471 |
| Alanine dehydrogenase | PRK06046 |
| GNAT | cd04301, pfam00583, pfam13444, pfam13508, PRK10314, TIGR04045 |
| MATE | cd13143, COG0534, pfam14667, PRK09575, TIGR00797 |
| B3/B4 | COG3382 |
| NimA | COG3467, pfam01243, pfam12900 |
| PnuC | COG3201, pfam04973, TIGR01528 |
| SugE | COG2076, PRK11431 |

Supplementary Table 2: Compounds tested as potential ligand of the Gd4v RNA motif. The highest concentrations that were used are listed. All compounds have been tested in in-line probing reactions with the construct *95 Csp* and *95 Dsu* and in transcription termination assays with the constructs *170 Csp* and *162 Dsu*. Compounds that showed apparent structural modulation in an in-line reaction with the *95 Csp* construct are highlighted in gray.

| **compound** | **highest concentration tested** |
| --- | --- |
| **guanidine** | 10 mM |
| **methyl-guanidine** | 10 mM |
| **amino-guanidine** | 10 mM |
| **hydroxy-guanidine** | 10 mM |
| **arginine** | 10 mM |
| **urea** | 10 mM |
| **ppGpp** | 100 μM |
| **pppGpp** | 100 μM |
| **NTPs** | 2.5 mM |
| **dNTPs** | 1 mM |
| **cAMP** | 1 mM |
| **cGMP** | 1 mM |
| **c-di-GMP** | 1 mM |
| **SAM** | 1 mM |
| **biotin** | 100 uM |
| **isoleucine** | 1 mM |
| **glutamate** | 100 μM |
| **lysine** | 1 mM |
| **valine** | 1 mM |
| **alanine** | 1 mM |
| **threonine** | 1 mM |
| **agmatine** | 1 mM |
| **proline** | 1 mM |
| **leucin** | 1 mM |
| **ornithine** | 1 mM |
| **hypoxanthine** | 5 mM |
| **xanthine** | 1 mM |
| **creatine** | 1 mM |
| **Creatinine** | 1 mM |

Supplementary Table 3: Compounds tested as substrates for Gd4v-associated GNAT protein.

|  | guanidine |  | lysine |  | leucine |  | 4-aminobenzoate |
| --- | --- | --- | --- | --- | --- | --- | --- |
|  | aminoguanidine |  | serine |  | ornithine |  | cimetidine |
|  | methylguanidine |  | histidine |  | cadaverine |  | streptomycine |
|  | hydroxyguanidine |  | asparagine |  | spermidine |  | gentamycine |
|  | arginine |  | tyrosine |  | putrescine |  | capromycine |
|  | canavanine |  | phenylalanine |  | taurin |  | kanamycine |
|  | threonine |  | glycine |  | creatinine |  | S-triazine |
|  | aspartate |  | isoleucine |  | citrulline |  |  |
|  | glutamine |  | valine |  | creatine |  |  |
|  | methionine |  | proline |  | agmatine |  |  |
|  | glutamate |  | hydroxyproline |  | homoserine |  |  |
|  | tryptophane |  | alanine |  | dihydroneopterin |  |  |

**Supplementary Table 4: Sequence of oligonucleotides used for the analysis of the *Gd4v RNA* motif.** Sequences were taken from *Cloacibacillus sp*., *Dialister succinatiphilus* and *Mitsuokella jalaludinii*. The T7 RNA Polymerase promoter is shown in green, the T5 RNA Polymerase promoter in blue. Lowercase letters identify non-genomic guanosine nucleotides added to enhance transcription. Nucleotides that were mutated relative to the wt sequence are shaded yellow. Start codons are shaded gray.

| **Construct name** | **Sequence (5´-3´)** |
| --- | --- |
| 95 Csp | TAATACGACTCACTATAggAATGCTTCCTCCGCCACCGGGCGGAGGGTGAAAAGCGTCCACGGTCTCCCGTAGGTACGGCGCGCTCTGCGCGTTACAAGGGAGCCGCGGGCG |
| 95 Csp M1 (G75C) | TAATACGACTCACTATAggAATGCTTCCTCCGCCACCGGGCGGAGGGTGAAAAGCGTCCACGGTCTCCCGTAGCTACGGCGCGCTCTGCGCGTTACAAGGGAGCCGCGGGCG |
| 95 Csp M2 (U58A) | TAATACGACTCACTATAggAATGCTTCCTCCGCCACCGGGCGGAGGGTGAAAAGCGTCCACGGTCTCCCGTAGGAACGGCGCGCTCTGCGCGTTACAAGGGAGCCGCGGGCG |
| 81 Csp | TAATACGACTCACTATAggAATGCTTCCTCCGCCACCGGGCGGAGGGTGAAAAGCGTCCACGGTCTCCCGTAGGTACGGCGCGCTCTGCGCGTTACAA |
| 95 Dsu | TAATACGACTCACTATAggAAACTCTTCCCTGTCACCGGATAGGGAGCCAAGAGGGTTTATTCCTGCTGCAGGTAATATGGCGAAAACCATTGAAAGGCAGGAATAAATCCT |
| 170 Csp | TCATAAAAAATTTATTTGCTTTGTGAGCGGATAACAATTATAATAAATGCTTCCTCCGCCACCGGGCGGAGGGTGAAAAGCGTCCACGGTCTCCCGTAGGTACGGCGCGCTCTGCGCGTTACAAGGGAGCCGCGGGCGCTTTATTTTTTATAAGGAGGGTTCTGCATGTATATCAAGCCGTTTGAAGTGGAGGAATGGATGAACGCATGGGAAAC |
| 162 Dsu | TCATAAAAAATTTATTTGCTTTGTGAGCGGATAACAATTATAATAAAACTCTTCCCTGTCACCGGATAGGGAGCCAAGAGGGTTTATTCCTGCTGCAGGTAATATGGCGAAAACCATTGAAAGGCAGGAATAAATCCTCTTTTTTTTAAGGAGGTTATATCATGAACATTAAACCCTTTGCTGTCGAAGAATGGATGAATGCCTATG |
| 105 Mja | TAATACGACTCACTATAggAACACCGCTTCTGTCACCGGACAGGAGAAATGGGGTGTCTGGGAGTTGTCGTAGGTACGGTATGGTTGCATGCTATAAGACAATTCCTGGGCACCCCTGTTTT |

References

1. Lenkeit F, Eckert I, Hartig JS, Weinberg Z. Discovery and characterization of a fourth class of guanidine riboswitches. Nucleic Acids Res 2020;48:12889–99.
