# Supplementary material for: A variant of guanidine-IV riboswitches exhibits evidence of a distinct ligand specificity
Source: RNA Biol. 2022 Dec 22;20(1):10–9. doi: 10.1080/15476286.2022.2160562 (PMC9788692; doi:10.1080/15476286.2022.2160562)
Supplement: Supplemental Material [file KRNB_A_2160562_SM7488.zip › SupplementaryFile1.pdf]

Supplementary File 1: alignments and information on downstream genes and taxonomy
Discovery and characterization of a variant of the fourth class of guanidine riboswitches

Felina Lenkeit & Iris Eckert & Malte Sinn & Franziskus Hauth & Jörg S.Hartig & Zasha Weinberg

Note: the presentation and explanatory text of this supplementary data on novel RNA motifs follows the pattern of a presentation of previously found conserved RNA motifs (Weinberg, et al., 2010).

Contents

1 G-IV-variant **2**
1.1 Taxa 2
1.2 Gene contexts 2
1.3 Conserved domains 11
1.4 Multiple-sequence alignment 13

1 G-IV-variant

1.1 Taxa

The taxonomy of each organism containing a putative G-IV-variant RNA is listed, with abbreviations identifying each hit (e.g., "Eco-1-1" and "Eco-1-2" might hypothetically represent two distinct RNAs in *E. coli*). The abbreviations will be used to identify each individual G-IV-variant RNA in Sections 1.2 and 1.4:.

**abbrev. of hits**	**taxonomy of species**
Bru-1-1	Bacteria Actinobacteria Bifidobacteriales Bifidobacteriaceae *Bifidobacterium ruminantium* DSM 6489
Cin-1-1	Bacteria Actinobacteria Coriobacteridae Coriobacteriales Coriobacterineae Coriobacteriaceae *Collinsella intestinalis* DSM 13280
Cph-1-1	Bacteria Actinobacteria Coriobacteriia Coriobacteriales Coriobacteriaceae *Collinsella phocaeensis*
Bxy-1-1	Bacteria Bacteroidetes Bacteroidia Bacteroidales Bacteroidaceae *Bacteroides xylanolyticus*
Lfe-1-1	Bacteria Firmicutes Bacilli Lactobacillales Lactobacillaceae *Lactobacillus fermentum*
Lin-1-1	Bacteria Firmicutes Bacilli Lactobacillales Lactobacillaceae *Lactobacillus ingluviei* DSM 15946
Lpe-1-1	Bacteria Firmicutes Bacilli Lactobacillales Lactobacillaceae *Lactobacillus pentosiphilus*
Lsi-1-1	Bacteria Firmicutes Bacilli Lactobacillales Lactobacillaceae *Lactobacillus silagei* JCM 19001
Lsi-2-1	Bacteria Firmicutes Bacilli Lactobacillales Lactobacillaceae *Lactobacillus silagincola*
Lsi-3-1	Bacteria Firmicutes Bacilli Lactobacillales Lactobacillaceae *Lactobacillus similis* DSM 23365 = JCM 2765
Lva-1-1	Bacteria Firmicutes Bacilli Lactobacillales Lactobacillaceae *Lactobacillus vaccinostercus* DSM 20634
Cbe-1-1	Bacteria Firmicutes Clostridia Clostridiales Clostridiaceae *Clostridium beijerinckii*
Cbe-2-1	Bacteria Firmicutes Clostridia Clostridiales Clostridiaceae *Clostridium beijerinckii* HUN142
Cpa-1-1	Bacteria Firmicutes Clostridia Clostridiales Clostridiaceae *Clostridium papyrosolvens* DSM 2782
Csa-1-1	Bacteria Firmicutes Clostridia Clostridiales Clostridiaceae *Clostridium saccharoperbutylacetonicum*
Csa-2-1	Bacteria Firmicutes Clostridia Clostridiales Clostridiaceae *Clostridium sartagoforme* AAU1
Csp-1-1	Bacteria Firmicutes Clostridia Clostridiales Clostridiaceae *Clostridium sp.* 12(A)
Csp-2-1	Bacteria Firmicutes Clostridia Clostridiales Clostridiaceae *Clostridium sp.* 2-1
Csp-3-1	Bacteria Firmicutes Clostridia Clostridiales Clostridiaceae *Clostridium sp.* AT4
Csp-4-1	Bacteria Firmicutes Clostridia Clostridiales Clostridiaceae *Clostridium sp.* Marseille-P2415
Csp-5-1	Bacteria Firmicutes Clostridia Clostridiales Clostridiaceae *Clostridium sp.* SY8519
Eti-1-1	Bacteria Firmicutes Clostridia Clostridiales ClostridialesFamily XIII. Incertae Sedis *Emergencia timonensis*
Ere-1-1	Bacteria Firmicutes Clostridia Clostridiales Eubacteriaceae *Eubacterium rectale* ATCC 33656
Pal-1-1	Bacteria Firmicutes Clostridia Clostridiales Eubacteriaceae *Pseudoramibacter alactolyticus* ATCC 23263
[ae-1-1	Bacteria Firmicutes Clostridia Clostridiales Lachnospiraceae [Clostridium] aerotolerans DSM 5434
[am-1-1	Bacteria Firmicutes Clostridia Clostridiales Lachnospiraceae [Clostridium] aminophilum DSM 10710
[re-1-1	Bacteria Firmicutes Clostridia Clostridiales Lachnospiraceae [Eubacterium] rectale
Lsp-1-1	Bacteria Firmicutes Clostridia Clostridiales Lachnospiraceae *Lachnoclostridium sp.* An131
Lba-1-1	Bacteria Firmicutes Clostridia Clostridiales *Lachnospiraceae bacterium* A2
Lba-2-1	Bacteria Firmicutes Clostridia Clostridiales *Lachnospiraceae bacterium* Marseille-P3773
Osp-1-1	Bacteria Firmicutes Clostridia Clostridiales Lachnospiraceae *Oribacterium sp.* C9
Rfa-1-1	Bacteria Firmicutes Clostridia Clostridiales Lachnospiraceae *Roseburia faecis*
Osp-2-1	Bacteria Firmicutes Clostridia Clostridiales Oscillospiraceae *Oscillibacter sp.* ER4
Psp-1-1	Bacteria Firmicutes Clostridia Clostridiales *Pseudoflavonifractor sp.* An184
Ebi-1-1	Bacteria Firmicutes Erysipelotrichi Erysipelotrichales Erysipelotrichaceae *Eubacterium biforme* DSM 3989
Lfe-2-1	Bacteria Firmicutes Lactobacillales Lactobacillaceae *Lactobacillus fermentum* 28-3-CHN
Smo-1-1	Bacteria Firmicutes Negativicutes Selenomonadales Acidaminococcaceae *Succinispira mobilis* DSM 6222
Mja-1-1	Bacteria Firmicutes Negativicutes Selenomonadales Selenomonadaceae *Mitsuokella jalaludinii*
Ppr-1-1	Bacteria Firmicutes Negativicutes Selenomonadales Sporomusaceae *Pelosinus propionicus* DSM 13327
Dsu-1-1	Bacteria Firmicutes Negativicutes Selenomonadales Veillonellaceae *Dialister succinatiphilus* YIT 11850
Mel-1-1	Bacteria Firmicutes Negativicutes Selenomonadales Veillonellaceae *Megasphaera elsdenii* DSM 20460
Msp-1-1	Bacteria Firmicutes Negativicutes Selenomonadales Veillonellaceae *Megasphaera sp.* BL7
Cma-1-1	Bacteria Firmicutes Negativicutes Veillonellales Veillonellaceae *Caecibacter massiliensis*
Mel-2-1	Bacteria Firmicutes Negativicutes Veillonellales Veillonellaceae *Megasphaera elsdenii*
Mel-3-1	Bacteria Firmicutes Negativicutes Veillonellales Veillonellaceae *Megasphaera elsdenii* T81
Vma-1-1	Bacteria Firmicutes Negativicutes Veillonellales Veillonellaceae *Veillonella magna* DSM 19857
Csp-6-1	Bacteria Synergistetes Synergistia Synergistales Synergistaceae *Cloacibacillus sp.* An23
Dsa-1-1	Bacteria Synergistetes Synergistia Synergistales Synergistaceae *Dethiosulfovibrio salsuginis*
env-1 to env-345	environmental samples

1.2 Gene contexts

Each G-IV-variant RNA (indicated by "RNA→") is listed. For each hit, the downstream genes predicted to reside in a regulated operon are listed. If the nearest downstream gene is encoding in the opposite strand (and therefore presumed to not be a part of a regulated operon), then that gene is still depicted. Some environmental sequences and some RefSeq entries lack gene annotations, and so no genes are available for such sequences. The direction of each gene is indicated with an arrow (→), and each predicted conserved domain in the gene is named. Conserved domains associated with more than one G-IV-variant RNA are assigned a color; other domains are gray. Information about these conserved domains is given in Section 1.3. The accession of the sequence containing each G-IV-variant RNA is given in the column named "Seq. accession". Accessions beginning with "NC_", "NS_", "NW_" or "NZ_" are contained in RefSeq. Other accession refer to environmental samples. The strand is indicated by a plus symbol ('+'), indicating that the RNA is on the sense strand of the containing genomic DNA sequence, or by a minus symbol ('-'), indicating that the RNA is on the reverse-complement strand. Nucleotide coordinates are given for the 5′ and 3′ boundaries of each G-IV-variant RNA. If the 5′ coordinate is greater than the 3′ coordinate, the RNA is present on the reverse-complement strand of the containing genomic DNA sequence. The plus/minus symbol is redundant with the nucleotide coordinates, since the nucleotide coordinates also indicate the strand. Each hit is denoted by an abbreviation (like "Eco-1-1") that refers to a taxonomy given in Section 1.1.

abbrev.	Seq. accession		5′ at	3′ at	genes
Lin-1-1	NZ_AZFK01000087.1	+	39743	39836	RNA→ PRK06046 (PRK06046)OCDMu (COG2423)→ AspB (COG0436)PRK07324 (PRK07324)→ hypo→
env-1	scaffold19895_1_MH0040	+	1423	1520	RNA→ PRK07324 (PRK07324)→
env-2	BMHBC_5109	-	9642	9550	RNA→ Acetyltransf_1 (pfam00583)Acetyltransf_10 (pfam13673)→
env-3	Pasolli2019-5821-25	-	2465	2372	RNA→ AAT_like (cd00609)viomycin_VioD (TIGR03947)→
env-4	UMGS433-9	+	26216	26308	RNA→ AAT_like (cd00609)viomycin_VioD (TIGR03947)→
env-5	scaffold8621_2_V1.CD-8	-	3443	3351	RNA→ PRK07324 (PRK07324)AAT_like (cd00609)→
env-6	Pasolli2019-5820-5	-	38905	38813	RNA→ AAT_like (cd00609)viomycin_VioD (TIGR03947)→
Dsu-1-1	NZ_JH591188.1	-	448757	448664	RNA→ PRK07324 (PRK07324)AAT_like (cd00609)→
env-7	Pasolli2019-5818-0	+	25894	25987	RNA→ AAT_like (cd00609)viomycin_VioD (TIGR03947)→
env-8	scaffold57138_1_MH0052	-	2427	2334	RNA→ AspB (COG0436)PRK07324 (PRK07324)→
env-9	OGUG01003254.1	-	353	260	RNA→ PRK07324 (PRK07324)→
env-10	Pasolli2019-5817-14	+	15021	15115	RNA→ AspB (COG0436)viomycin_VioD (TIGR03947)→
env-11	OGKD01004400.1	+	4339	4433	RNA→ PRK07324 (PRK07324)→
env-12	DJPC01000089.1	+	10005	10098	RNA→ PRK07324 (PRK07324)AAT_like (cd00609)→
env-13	DBLC01000136.1	+	5201	5294	RNA→ PRK07324 (PRK07324)AAT_like (cd00609)→
env-14	DNXJ01000091.1	+	2858	2951	RNA→ PRK07324 (PRK07324)AAT_like (cd00609)→ PRK07324 (PRK07324)AAT_like (cd00609)→
env-15	HCF14C_1_1_1_newblercontig07176	-	221	132	RNA→ hypo→
env-16	OGEZ01002805.1	-	144	44	RNA→
env-17	OGGQ01000011.1	-	197186	197087	RNA→ PRK09856 (PRK09856)YcjR (COG1082)→ PRK09856 (PRK09856)AP_endonuc_2 (pfam01261)→ frlB (PRK11382)AgaS (COG2222)→ PRK09813 (PRK09813)RbsK (COG0524)→ PRK09813 (PRK09813)RbsK (COG0524)→ COG2159 (COG2159)→ TIGR00149_YjbQ (TIGR00149)UPF0047 (pfam01894)→
env-18	DJOA01000008.1	+	58677	58777	RNA→ Acetyltransf_1 (pfam00583)Acetyltransf_7 (pfam13508)→ hypo→ Lactamase_B (smart00849)ElaC (COG1234)→
Lfe-1-1	NZ_CP017151.1	+	1886818	1886921	RNA→ hypo→ AspB (COG0436)PRK07324 (PRK07324)→
Lfe-2-1	NZ_GG704699.1	-	92464	92374	RNA→ PRK06046 (PRK06046)OCDMu (COG2423)→ PRK06046 (PRK06046)OCDMu (COG2423)→ hypo→ hypo→ AspB (COG0436)PRK07324 (PRK07324)→ AspB (COG0436)PRK07324 (PRK07324)→
env-19	Ga0134523_1315158	-	162	72	RNA→ hypo→
env-20	OGEP01028537.1	+	1023	1113	RNA→
env-21	OLFU01008364.1	+	2844	2937	RNA→ PRK06046 (PRK06046)OCDMu (COG2423)→
env-22	Ga0208297_1045394	-	393	298	RNA→ PRK07324 (PRK07324)→
env-23	DJPC01000098.1	-	10304	10218	RNA→ hypo→
env-24	Pasolli2019-3970-106	-	8506	8410	RNA→ hypo→
Eti-1-1	NZ_FLKM01000008.1	+	1660007	1660103	RNA→ MATE_yoeA_like (cd13138)Polysacc_synt_C (pfam14667)→
env-25	Pasolli2019-5859-62	-	9940	9845	RNA→ Acetyltransf_1 (pfam00583)Acetyltransf_7 (pfam13508)→ hypo→
env-26	DOM012_scaffold36545_2	-	223	128	RNA→ hypo→
Mel-3-1	NZ_JHXC01000016.1	+	52023	52117	RNA→ Acetyltransf_1 (pfam00583)Acetyltransf_7 (pfam13508)→ hypo→

Mja-1-1	NZ_CYYU01000028.1	+	9111	9205	**RNA**→ Acetyltransf_1 (pfam00583)Acetyltransf_7 (pfam13508)→ hypo→
env-27	AUXO015988238.1	+	2419	2514	**RNA**→ Acetyltransf_1 (pfam00583)Acetyltransf_7 (pfam13508)→
env-28	OIYF01000927.1	-	370	276	**RNA**→ NAT_SF (cd04301)Acetyltransf_7 (pfam13508)→
env-29	HighMDraftT1_c001256	-	367	273	**RNA**→ ElaA (COG2153)Acetyltransf_7 (pfam13508)→
Mel-2-1	NZ_NQMW01000011.1	+	66706	66800	**RNA**→ Acetyltransf_1 (pfam00583)Acetyltransf_7 (pfam13508)→ hypo→
Mel-1-1	NC_015873.1	-	2212412	2212318	**RNA**→ Acetyltransf_1 (pfam00583)Acetyltransf_7 (pfam13508)→ hypo→
env-30	OIWT01000070.1	+	18419	18513	**RNA**→ Acetyltransf_1 (pfam00583)Acetyltransf_7 (pfam13508)→ hypo→
env-31	DLF014_scaffold108_1	+	1977	2071	**RNA**→ Acetyltransf_1 (pfam00583)Acetyltransf_7 (pfam13508)→ EPS_sugtrans (TIGR03025)→
env-32	Pasolli2019-5853-6	+	29321	29415	**RNA**→ Acetyltransf_1 (pfam00583)Acetyltransf_7 (pfam13508)→ hypo→
Msp-1-1	NZ_APHX01000005.1	-	17395	17300	**RNA**→ Acetyltransf_1 (pfam00583)Acetyltransf_7 (pfam13508)→
env-33	OGEO01011079.1	-	441	346	**RNA**→ Acetyltransf_1 (pfam00583)Acetyltransf_7 (pfam13508)→
env-34	OGEH01002006.1	-	5239	5144	**RNA**→ Acetyltransf_1 (pfam00583)Acetyltransf_7 (pfam13508)→
env-35	OGUG01022925.1	+	120	215	**RNA**→ Acetyltransf_1 (pfam00583)Acetyltransf_7 (pfam13508)→
env-36	DLM005_scaffold57719_1	-	5469	5374	**RNA**→ Acetyltransf_1 (pfam00583)Acetyltransf_7 (pfam13508)→ hypo→ hypo→
env-37	OGLZ01013223.1	-	368	273	**RNA**→ ElaA (COG2153)Acetyltransf_7 (pfam13508)→
Cma-1-1	NZ_LT608311.1	-	1046074	1045979	**RNA**→ Acetyltransf_1 (pfam00583)Acetyltransf_7 (pfam13508)→ hypo→
env-38	Pasolli2019-5852-108	-	5202	5107	**RNA**→ Acetyltransf_1 (pfam00583)Acetyltransf_7 (pfam13508)→ hypo→
env-39	ONTA01000252.1	+	5632	5727	**RNA**→ Acetyltransf_1 (pfam00583)Acetyltransf_7 (pfam13508)→ hypo→
env-40	DLF014_scaffold67_1	-	4865	4771	**RNA**→ hypo→
env-41	OLFZ01026633.1	-	596	501	**RNA**→ Acetyltransf_1 (pfam00583)Acetyltransf_7 (pfam13508)→
env-42	Pasolli2019-5902-311	+	1668	1763	**RNA**→ Acetyltransf_1 (pfam00583)Acetyltransf_10 (pfam13673)→
env-43	Pasolli2019-5906-25	+	20569	20664	**RNA**→ Acetyltransf_1 (pfam00583)Acetyltransf_7 (pfam13508)→ EPS_sugtrans (TIGR03025)→
env-44	4448815.3_Contig24945	+	263	357	**RNA**→
env-45	4448812.3_Contig5522	+	1254	1348	**RNA**→ ElaA (COG2153)Acetyltransf_5 (pfam13444)→
env-46	Ga0129306_1000055	+	177747	177838	**RNA**→ PRK07324 (PRK07324)AAT_like (cd00609)→
Lva-1-1	NZ_AYYY01000007.1	-	38090	37992	**RNA**→ hypo→
env-47	07_22_PECTIN_DNA_scaffold68818_2	-	612	515	**RNA**→ Acetyltransf_1 (pfam00583)Acetyltransf_7 (pfam13508)→
env-48	BMHB3a_c118857	-	577	483	**RNA**→ hypo→
env-49	DEHJ01000057.1	+	3476	3570	**RNA**→ Acetyltransf_1 (pfam00583)Acetyltransf_7 (pfam13508)→ hypo→ hypo→ hypo→ hypo→
[am-1-1	NZ_JONJ01000001.1	-	107243	107149	**RNA**→ hypo→ Acetyltransf_1 (pfam00583)Acetyltransf_7 (pfam13508)→ Acetyltransf_1 (pfam00583)→
env-50	Ga0256405_10080349	+	659	752	**RNA**→ Acetyltransf_1 (pfam00583)Acetyltransf_7 (pfam13508)→ hypo→ Acetyltransf_1 (pfam00583)→
env-51	Ga0116649_1130823	-	138	46	**RNA**→ ←hypo
Bru-1-1	NZ_JHWQ01000001.1	-	378062	377970	**RNA**→ hypo→ hypo→ hypo→ 2_A_01_02 (TIGR00880)MFS_1 (pfam07690)→ efflux_EmrB (TIGR00711)MFS_1 (pfam07690)→
env-52	Pasolli2019-4992-14	-	50645	50548	**RNA**→ MATE_yoeA_like (cd13138)Polysacc_synt_C (pfam14667)→
env-53	scaffold4176_28_O2.UC-18	-	643	546	**RNA**→ NorM (COG0534)matE (TIGR00797)→
env-54	Ga0256406_1013254	-	2583	2490	**RNA**→ PRK07324 (PRK07324)AAT_like (cd00609)→
env-55	DKQZ01000042.1	-	2373	2280	**RNA**→ PRK07324 (PRK07324)AAT_like (cd00609)→
Lsi-1-1	NZ_BCMG01000001.1	-	244568	244461	**RNA**→ PRK06046 (PRK06046)OCDMu (COG2423)→ AspB (COG0436)PRK07324 (PRK07324)→
Lpe-1-1	NZ_BCMH01000010.1	+	91061	91168	**RNA**→ PRK06046 (PRK06046)OCDMu (COG2423)→ AspB (COG0436)PRK07324 (PRK07324)→
env-56	Ga0214925_100014	-	21071	20965	**RNA**→ PRK06046 (PRK06046)OCDMu (COG2423)→ PRK07324 (PRK07324)AAT_like (cd00609)→
Lsi-2-1	NZ_BCMJ01000002.1	-	349956	349850	**RNA**→ PRK06046 (PRK06046)OCDMu (COG2423)→ PRK07324 (PRK07324)AAT_like (cd00609)→
Lsi-3-1	NZ_BBAD01000017.1	-	7256	7153	**RNA**→ PRK06046 (PRK06046)OCDMu (COG2423)→ PRK07324 (PRK07324)AAT_like (cd00609)→
env-57	Ga0129311_1122818	+	318	420	**RNA**→ hypo→
env-58	Ga0129314_1015585	+	45	147	**RNA**→ PRK07324 (PRK07324)AAT_like (cd00609)→ AspB (COG0436)PRK07324 (PRK07324)→
env-59	Ga0129314_1009844	-	2009	1911	**RNA**→ RhaT (COG0697)2A78 (TIGR00950)→
env-60	Pasolli2019-14782-101	+	1328	1427	**RNA**→ COG2316 (COG2316)HDc (smart00471)→
env-61	DLM013_scaffold43269_1	+	8115	8213	**RNA**→ HDc (smart00471)COG2316 (COG2316)→
env-62	Ga0134439_1007031	-	1248	1150	**RNA**→ HDc (smart00471)COG2316 (COG2316)→
env-63	DBPY01000030.1	+	149856	149949	**RNA**→ HDc (smart00471)COG2316 (COG2316)→
env-64	OLGO01001319.1	+	14304	14397	**RNA**→ HDc (smart00471)COG2316 (COG2316)→
env-65	Pasolli2019-14739-14	+	27946	28039	**RNA**→ COG2316 (COG2316)HDc (smart00471)→

env-66	UMGS1432-9	-	34384	34288	**RNA**→ COG2316 (COG2316)HDc (smart00471)→
env-67	Pasolli2019-14746-23	-	18856	18759	**RNA**→ COG2316 (COG2316)HDc (smart00471)→
Cph-1-1	NZ_FQLR01000004.1	+	1411383	1411480	**RNA**→ HDc (smart00471)COG2316 (COG2316)→
env-68	OGDD01009246.1	+	688	782	**RNA**→ HDc (smart00471)COG2316 (COG2316)→
Cin-1-1	NZ_GG692710.1	+	613294	613390	**RNA**→ HDc (smart00471)COG2316 (COG2316)→
env-69	longitudinal_131_27	+	46064	46160	**RNA**→ HDc (smart00471)COG2316 (COG2316)→
env-70	DLM013_scaffold1548_11	-	2180	2083	**RNA**→ HDc (smart00471)COG2316 (COG2316)→
env-71	Pasolli2019-14781-201	-	1879	1780	**RNA**→ COG2316 (COG2316)HDc (smart00471)→
env-72	UMGS1653-7	-	17803	17706	**RNA**→ COG2316 (COG2316)HDc (smart00471)→
env-73	Pasolli2019-15241-235	-	1534	1440	**RNA**→ AAT_like (cd00609)viomycin_VioD (TIGR03947)→ OCDMu (COG2423)ala_DH_arch (TIGR02371)→
env-74	Ga0134448_1006703	-	1319	1227	**RNA**→ PRK07324 (PRK07324)AAT_like (cd00609)→
env-75	scaffold2915_3_MH0013	+	441	534	**RNA**→ PRK07324 (PRK07324)AAT_like (cd00609)→ PRK06046 (PRK06046)OCDMu (COG2423)→
env-76	scaffold59035_1_MH0012	+	160	253	**RNA**→ PRK07324 (PRK07324)AAT_like (cd00609)→ PRK06046 (PRK06046)OCDMu (COG2423)→
env-77	OLGY01006160.1	-	3202	3109	**RNA**→ PRK07324 (PRK07324)AAT_like (cd00609)→ PRK07324 (PRK07324)→ PRK06046 (PRK06046)OCDMu (COG2423)→ PRK06046 (PRK06046)OCDMu (COG2423)→
env-78	DBKY01000146.1	+	830	923	**RNA**→ PRK07324 (PRK07324)AAT_like (cd00609)→ PRK06046 (PRK06046)OCDMu (COG2423)→
env-79	Pasolli2019-15246-107	+	4395	4488	**RNA**→ AAT_like (cd00609)viomycin_VioD (TIGR03947)→ OCDMu (COG2423)ala_DH_arch (TIGR02371)→
env-80	DHKA01000084.1	-	13970	13877	**RNA**→ PRK07324 (PRK07324)AAT_like (cd00609)→ PRK06046 (PRK06046)OCDMu (COG2423)→
env-81	Pasolli2019-15247-12	-	4890	4797	**RNA**→ AAT_like (cd00609)viomycin_VioD (TIGR03947)→ OCDMu (COG2423)ala_DH_arch (TIGR02371)→
env-82	OGFF01011754.1	+	194	287	**RNA**→ AspB (COG0436)PRK07324 (PRK07324)→
env-83	Ga0129307_1017473	+	1976	2074	**RNA**→ ←hypo
env-84	AUXO010132685.1	-	429	332	**RNA**→ PRK07324 (PRK07324)AAT_like (cd00609)→
env-85	scaffold26622_3_MH0032	+	443	537	**RNA**→ AspB (COG0436)PRK07324 (PRK07324)→
env-86	C2540027_1_MH0048	+	452	546	**RNA**→ PRK07324 (PRK07324)→
env-87	OGGT01020056.1	-	632	538	**RNA**→ PRK07324 (PRK07324)→
env-88	OGGM01000031.1	-	79706	79615	**RNA**→ NAT_SF (cd04301)Acetyltransf_7 (pfam13508)→
env-89	Ga0134432_165089	+	80	175	**RNA**→ MSMEG_0567_GNAT (TIGR04045)→
env-90	DCCL01000127.1	-	17787	17689	**RNA**→ NorM (COG0534)vmrA (PRK09575)→
env-91	Ga0116646_1051066	-	744	647	**RNA**→ TPR (sd00006)→
env-92	Ga0116620_1022832	+	352	447	**RNA**→ vmrA (PRK09575)MATE_MepA_like (cd13143)→
env-93	Ga0116619_1035586	+	355	450	**RNA**→ vmrA (PRK09575)MATE_MepA_like (cd13143)→
env-94	Ga0116649_1093153	+	241	336	**RNA**→
env-95	OGCY01010614.1	-	140	39	**RNA**→
Rfa-1-1	NZ_CVRR01000060.1	-	164783	164682	**RNA**→ PRK07324 (PRK07324)AAT_like (cd00609)→
env-96	OGMU01000248.1	-	1822	1723	**RNA**→ PRK07324 (PRK07324)AAT_like (cd00609)→
env-97	DLM020_scaffold19295_1	+	158	259	**RNA**→ PRK07324 (PRK07324)AAT_like (cd00609)→
env-98	OGGZ01036916.1	+	169	270	**RNA**→ PRK07324 (PRK07324)AAT_like (cd00609)→
env-99	Ga0116646_1184072	+	121	217	**RNA**→
env-100	DHJV01000017.1	-	14847	14750	**RNA**→ NorM (COG0534)vmrA (PRK09575)→
env-101	Ga0172382_10987488	+	113	207	**RNA**→ PRK07324 (PRK07324)AAT_like (cd00609)→
env-102	Ga0129314_1006393	-	259	159	**RNA**→ hypo→
env-103	2013448673	+	117	213	**RNA**→ PRK06046 (PRK06046)OCDMu (COG2423)→ PRK06046 (PRK06046)OCDMu (COG2423)→
Csp-4-1	NZ_LT732526.1	+	23997	24093	**RNA**→ hypo→ HTH_XRE (smart00530)XRE (COG1476)→
Csp-6-1	NZ_NFJQ01000010.1	-	47835	47742	**RNA**→ PRK07324 (PRK07324)AAT_like (cd00609)→
env-104	Pasolli2019-15082-30	-	11984	11886	**RNA**→ AspB (COG0436)viomycin_VioD (TIGR03947)→ AspB (COG0436)PLN00175 (PLN00175)→
env-105	AUXO014618521.1	-	249	149	**RNA**→ PRK07324 (PRK07324)→
Pal-1-1	NZ_GL622359.1	-	970773	970683	**RNA**→ hypo→ Acetyltransf_1 (pfam00583)Acetyltransf_7 (pfam13508)→ EamA (pfam00892)→
env-106	Pasolli2019-4718-13	+	26619	26718	**RNA**→ YitT (COG1284)DUF2179 (pfam10035)→ SMC_prok_B (TIGR02168)→
env-107	DGVX01000010.1	+	22339	22442	**RNA**→ PRK07324 (PRK07324)AAT_like (cd00609)→ PRK06046 (PRK06046)OCDMu (COG2423)→ OCDMu (COG2423)PRK08618 (PRK08618)→ Argininosuccinate_lyase (cd01359)PRK00855 (PRK00855)→
env-108	JGI24712J26585_10234890	+	265	367	**RNA**→ PRK07324 (PRK07324)→
env-109	JGI24712J26585_10279194	+	265	367	**RNA**→
env-110	JGI24709J26583_10014374	+	2081	2183	**RNA**→ PRK07324 (PRK07324)AAT_like (cd00609)→

env-111	scaffold70770_1_V1.CD-9	-	422	325	**RNA**→ PRK07324 (PRK07324)AAT_like (cd00609)→
env-112	08_07_XOS_DNA_scaffold77589_1	+	315	415	**RNA**→ PRK07324 (PRK07324)AAT_like (cd00609)→
env-113	Ga0134523_1003125	-	4301	4201	**RNA**→ PRK07324 (PRK07324)AAT_like (cd00609)→ AspB (COG0436)PRK07777 (PRK07777)→ DUF1848 (pfam08902)→
env-114	Ga0129308_1003761	-	3027	2927	**RNA**→ PRK07324 (PRK07324)AAT_like (cd00609)→ COG3603 (COG3603)ACT_7 (pfam13840)→
Dsa-1-1	NZ_FXBB01000002.1	-	6826	6728	**RNA**→ PRK06046 (PRK06046)OCDMu (COG2423)PRK07324 (PRK07324)→
env-115	Ga0075011_10777787	+	92	190	**RNA**→ PRK07324 (PRK07324)→
env-116	DFCS01000020.1	+	123510	123609	**RNA**→ PBP2_Fbp_like_2 (cd13547)phnS2 (TIGR03261)→
env-117	DEYX01000304.1	+	17591	17690	**RNA**→ AspB (COG0436)PRK07324 (PRK07324)→
env-118	Ga0121719_101113	+	1371	1470	**RNA**→ AfuA (COG1840)phnS2 (TIGR03261)→ 3a0106s03 (TIGR00971)AfuA (COG1840)→
env-119	DGKO01000050.1	+	14826	14925	**RNA**→ AfuA (COG1840)phnS2 (TIGR03261)→ PBP2_Fbp_like_2 (cd13547)→ FbpB (COG1178)PhnU2 (TIGR03262)→ AAA (smart00382)PotA (COG3842)→ PRK09977 (PRK09977)SapB (COG1285)→
env-120	Ga0121590_113680	-	308	209	**RNA**→
env-121	Pasolli2019-4509-124	+	174	273	**RNA**→ AAT_like (cd00609)viomycin_VioD (TIGR03947)→ AspB (COG0436)PLN00175 (PLN00175)→
Vma-1-1	NZ_AUAN01000013.1	-	19019	18925	**RNA**→ AspB (COG0436)PRK07324 (PRK07324)→
env-122	scaffold6413_1_MH0054	-	6578	6483	**RNA**→ PRK07324 (PRK07324)→ PRK07324 (PRK07324)AAT_like (cd00609)→ DivIC (pfam04977)→
env-123	scaffold243288_1_MH0006	+	211	306	**RNA**→ PRK07324 (PRK07324)→ PRK07324 (PRK07324)AAT_like (cd00609)→
env-124	Ga0134547_1074131	-	181	86	**RNA**→ PRK07324 (PRK07324)→
env-125	scaffold24601_1_MH0056	-	2581	2486	**RNA**→ PRK07324 (PRK07324)AAT_like (cd00609)→
env-126	scaffold33841_9_MH0053	-	1478	1383	**RNA**→ PRK07324 (PRK07324)AAT_like (cd00609)→
env-127	Ga0116619_1116870	+	46	141	**RNA**→
env-128	DKVN01000102.1	-	2336	2238	**RNA**→ AspB (COG0436)PRK07324 (PRK07324)→ PRK06046 (PRK06046)OCDMu (COG2423)→
env-129	DKYQ01000005.1	-	37927	37825	**RNA**→ MATE_yoeA_like (cd13138)Polysacc_synt_C (pfam14667)→ PRK09856 (PRK09856)YcjR (COG1082)→ PRK09856 (PRK09856)AP_endonuc_2 (pfam01261)→ frlB (PRK11382)SIS_1 (cd05710)→ PRK09813 (PRK09813)RbsK (COG0524)→ PRK09813 (PRK09813)RbsK (COG0524)→ COG2159 (COG2159)→ TIGR00149_YjbQ (TIGR00149)UPF0047 (pfam01894)→
env-130	Ga0129309_1034323	+	546	647	**RNA**→ ←hypo
env-131	Ga0134442_1014104	-	1365	1264	**RNA**→ MATE_yoeA_like (cd13138)Polysacc_synt_C (pfam14667)→
env-132	Ga0134439_1018302	-	1192	1091	**RNA**→ MATE_yoeA_like (cd13138)Polysacc_synt_C (pfam14667)→
env-133	SRS015217_C2285664	-	471	370	**RNA**→ MATE_yoeA_like (cd13138)matE (TIGR00797)→
env-134	Ga0134523_1000815	+	57503	57604	**RNA**→ MATE_yoeA_like (cd13138)Polysacc_synt_C (pfam14667)→
env-135	Pasolli2019-4676-9	-	41446	41345	**RNA**→ MATE_yoeA_like (cd13138)Polysacc_synt_C (pfam14667)→ YcjR (COG1082)myo_inos_iolE (TIGR04379)→ AP_endonuc_2 (pfam01261)OH-pyruv-isom (TIGR03234)→ AgaS (COG2222)glmS (TIGR01135)→ RbsK (COG0524)myo_inos_iolC_N (TIGR04382)→ RbsK (COG0524)myo_inos_iolC_N (TIGR04382)→ COG2159 (COG2159)→ UPF0047 (pfam01894)TIGR00149_YjbQ (TIGR00149)→
env-136	EMG_10055766	-	512	417	**RNA**→ PRK07324 (PRK07324)AAT_like (cd00609)→
env-137	AUXO017867359.1	+	255	346	**RNA**→ PRK07324 (PRK07324)AAT_like (cd00609)→
env-138	_10843780	+	195	288	**RNA**→ PRK07324 (PRK07324)→
env-139	OLGD01003501.1	-	11407	11310	**RNA**→ AspB (COG0436)PRK07324 (PRK07324)→ PLN00175 (PLN00175)AspB (COG0436)→ SnoaL_2 (pfam12680)→ hypo→ hypo→
env-140	OLGB01027708.1	+	696	794	**RNA**→ AspB (COG0436)PRK07324 (PRK07324)→
env-141	UMGS1600-329	-	1897	1799	**RNA**→ AAT_like (cd00609)viomycin_VioD (TIGR03947)→ LMWPTP (cd16343)LMWPc (smart00226)→
env-142	DJOK01000052.1	-	22955	22858	**RNA**→ AspB (COG0436)PRK07324 (PRK07324)→
env-143	4491413.3_NODE_46556	-	5417	5320	**RNA**→ ←hypo
env-144	OGGI01025437.1	-	103	6	**RNA**→
env-145	Ga0169793_105935	+	576	673	**RNA**→ AspB (COG0436)PRK07324 (PRK07324)→
env-146	OLGH01046591.1	-	796	699	**RNA**→ AspB (COG0436)PRK07324 (PRK07324)→
env-147	OLGJ01044759.1	-	501	404	**RNA**→ ←hypo
env-148	OGJJ01070228.1	+	163	260	**RNA**→ PRK07324 (PRK07324)→
env-149	OGUJ01013785.1	+	487	584	**RNA**→ AspB (COG0436)PRK07324 (PRK07324)→
env-150	OIWM01023925.1	-	1214	1117	**RNA**→ Phage_GP20 (pfam06810)→ hypo→
env-151	Ga0134447_1044110	+	153	250	**RNA**→ ←hypo
env-152	scaffold96224_4_MH0006	-	1041	944	**RNA**→ PRK07324 (PRK07324)AAT_like (cd00609)→

env-153	OGFF01069906.1	-	480	383	RNA→ PRK07324 (PRK07324)AAT_like (cd00609)→
env-154	OGEP01005001.1	-	1617	1520	RNA→ PRK07324 (PRK07324)AAT_like (cd00609)→ PRK07324 (PRK07324)AAT_like (cd00609)→ PRK06046 (PRK06046)OCDMu (COG2423)→
env-155	Pasolli2019-15194-14	-	21564	21465	RNA→ AspB (COG0436)viomycin_VioD (TIGR03947)→ OCDMu (COG2423)ala_DH_arch (TIGR02371)→
env-156	UMGS1617-4	+	35975	36069	RNA→ AAT_like (cd00609)viomycin_VioD (TIGR03947)→ AspB (COG0436)viomycin_VioD (TIGR03947)→
env-157	SRS017307_C2293409	-	232	133	RNA→ PRK07324 (PRK07324)→
env-158	scaffold242831_4_MH0006	-	986	888	RNA→ PRK07324 (PRK07324)AAT_like (cd00609)→
env-159	OIYO01000246.1	-	21721	21622	RNA→ PRK07324 (PRK07324)AAT_like (cd00609)→
env-160	Pasolli2019-4723-105	-	9867	9764	RNA→ AAT_like (cd00609)viomycin_VioD (TIGR03947)→ AspB (COG0436)viomycin_VioD (TIGR03947)→ OCDMu (COG2423)ala_DH_arch (TIGR02371)→ COG3603 (COG3603)ACT_7 (pfam13840)→
env-161	DPHU01000046.1	-	56128	56028	RNA→ PRK07324 (PRK07324)AAT_like (cd00609)→
env-162	4491485.3_NODE_3922	-	36785	36685	RNA→ PRK07324 (PRK07324)AAT_like (cd00609)→
env-163	SRS015264_WUGC_scaffold_21721	+	999	1099	RNA→ PRK07324 (PRK07324)AAT_like (cd00609)→ hypo→
env-164	JGI2065J20421_1039381	+	537	635	RNA→
env-165	AUXO014646129.1	-	293	193	RNA→ PRK07324 (PRK07324)→
env-166	RUMENNODE_1527695_1	+	6864	6964	RNA→ PRK07324 (PRK07324)AAT_like (cd00609)→ Glyco_10 (smart00633)Glyco_hydro_42 (pfam02449)→ Glyco_hydro_35 (pfam01301)→ hypo→ hypo→
env-167	AUXO018293870.1	-	784	685	RNA→ PRK07324 (PRK07324)AAT_like (cd00609)→
env-168	Ga0256405_10007637	+	8025	8125	RNA→ AspB (COG0436)PRK07324 (PRK07324)→ PRK08912 (PRK08912)AspB (COG0436)→
env-169	Ga0256404_1139133	+	870	970	RNA→ PRK07324 (PRK07324)AAT_like (cd00609)→
env-170	RUMENNODE_4405231_1	+	5232	5332	RNA→ PRK07324 (PRK07324)→
env-171	Ga0120381_1144615	-	542	443	RNA→ PRK07324 (PRK07324)AAT_like (cd00609)→
env-172	Ga0256406_1227460	+	1	100	RNA→ PRK07324 (PRK07324)AAT_like (cd00609)→
env-173	Ga0256404_1048146	+	1260	1359	RNA→ PRK07324 (PRK07324)AAT_like (cd00609)→ PRK08912 (PRK08912)AspB (COG0436)→
env-174	Ga0256404_1004416	+	6792	6891	RNA→ PRK07324 (PRK07324)AAT_like (cd00609)→ PRK08912 (PRK08912)AspB (COG0436)→
env-175	Ga0120384_1010171	-	4261	4162	RNA→ PRK07324 (PRK07324)AAT_like (cd00609)→ PRK08912 (PRK08912)AspB (COG0436)→
env-176	Ga0120387_1026592	+	1910	2010	RNA→ PRK07324 (PRK07324)→
env-177	Ga0120381_1157373	+	310	411	RNA→ hypo→ PRK07324 (PRK07324)→
env-178	AUXO013029068.1	+	597	697	RNA→ PRK07324 (PRK07324)AAT_like (cd00609)→ PRK06046 (PRK06046)OCDMu (COG2423)→ PRK06046 (PRK06046)OCDMu (COG2423)→
env-179	Ga0120384_1031185	+	96	197	RNA→ PRK12314 (PRK12314)AAK_G5K_ProB (cd04242)→ ALDH_F18-19_ProA-GPR (cd07079)proA (PRK00197)→
env-180	Ga0120387_1087069	-	1076	975	RNA→ PRK07324 (PRK07324)→
env-181	Ga0208297_1087086	-	454	353	RNA→ AspB (COG0436)PRK07324 (PRK07324)→
env-182	Ga0256405_10066238	-	158	57	RNA→ ←hypo
env-183	RUMENNODE_2308512_22000	-	9789	9690	RNA→ PRK07324 (PRK07324)AAT_like (cd00609)→ PRK08912 (PRK08912)AspB (COG0436)→ PLN00175 (PLN00175)AAT_like (cd00609)→
env-184	Pasolli2019-15210-34	-	2362	2264	RNA→ AspB (COG0436)viomycin_VioD (TIGR03947)→ OCDMu (COG2423)ala_DH_arch (TIGR02371)→
env-185	Pasolli2019-15211-61	+	11665	11762	RNA→ AspB (COG0436)viomycin_VioD (TIGR03947)→ OCDMu (COG2423)ala_DH_arch (TIGR02371)→
env-186	UMGS530-15	+	34691	34788	RNA→ AspB (COG0436)viomycin_VioD (TIGR03947)→ OCDMu (COG2423)ala_DH_arch (TIGR02371)→
env-187	DJXY01000172.1	+	6215	6310	RNA→ PRK07324 (PRK07324)→
env-188	DGVH01000063.1	+	16414	16509	RNA→ PRK07324 (PRK07324)AAT_like (cd00609)→ AspB (COG0436)PRK07777 (PRK07777)→
env-189	Ga0075011_10162258	-	808	714	RNA→ Acetyltransf_1 (pfam00583)Acetyltransf_10 (pfam13673)→ hypo→
env-190	DJGY01000029.1	-	46927	46834	RNA→ Acetyltransf_1 (pfam00583)Acetyltransf_7 (pfam13508)→ hypo→
Csp-3-1	NZ_LN999828.1	-	1628254	1628157	RNA→ Acetyltransf_1 (pfam00583)Acetyltransf_7 (pfam13508)→
env-191	Ga0209064_1147995	-	373	274	RNA→ Acetyltransf_10 (pfam13673)→
env-192	BMHBC_98655	+	285	381	RNA→ Acetyltransf_1 (pfam00583)Acetyltransf_7 (pfam13508)→ hypo→
Csp-5-1	NC_015737.1	-	2782475	2782382	RNA→ Acetyltransf_1 (pfam00583)Acetyltransf_10 (pfam13673)→ hypo→ PRK09453 (PRK09453)MPP_superfamily (cd00838)→
env-193	AUXO011763636.1	+	468	561	RNA→
env-194	OGJW01025078.1	-	377	286	RNA→ YitT_membrane (pfam02588)→
[re-1-1	NZ_CYYW01000004.1	-	166812	166721	RNA→ YitT_membrane (pfam02588)DUF2179 (pfam10035)YitT_C (cd16380)→
env-195	OGKR01000347.1	+	198	289	RNA→ YitT_membrane (pfam02588)DUF2179 (pfam10035)YitT_C (cd16380)→ COG2326 (COG2326)poly_P_AMP_trns (TIGR03708)→

env-196	DLM006_scaffold4592_1	+	2627	2718	RNA→ YitT_membrane (pfam02588)DUF2179 (pfam10035)YitT_C (cd16380)→
env-197	SRS056259_LANL_scaffold_39528	+	496	587	RNA→ hypo→
env-198	scaffold3322_4_MH0046	+	776	867	RNA→ YitT (COG1284)DUF2179 (pfam10035)→
					PPK2 (pfam03976)COG2326 (COG2326)poly_P_AMP_trns (TIGR03708)→
env-199	DOM021_scaffold12910_3	+	30	121	RNA→ YitT (COG1284)→
env-200	OGEE01016093.1	-	801	710	RNA→ YitT_membrane (pfam02588)→
env-201	scaffold1570_2_MH0072	+	248	339	RNA→ YitT_membrane (pfam02588)DUF2179 (pfam10035)YitT_C (cd16380)→
					PPK2 (pfam03976)COG2326 (COG2326)poly_P_AMP_trns (TIGR03708)→
Ere-1-1	NC_012781.1	+	1295582	1295673	RNA→ YitT_membrane (pfam02588)DUF2179 (pfam10035)YitT_C (cd16380)→
env-202	scaffold3391_6_MH0043	+	139	231	RNA→ YitT (COG1284)DUF2179 (pfam10035)→
					PPK2 (pfam03976)COG2326 (COG2326)poly_P_AMP_trns (TIGR03708)→
env-203	DOM026_scaffold13057_4	-	4118	4028	RNA→ YitT_membrane (pfam02588)DUF2179 (pfam10035)YitT_C (cd16380)→
env-204	NLM004_scaffold8875_1	+	6002	6093	RNA→ YitT_membrane (pfam02588)DUF2179 (pfam10035)YitT_C (cd16380)→
env-205	Ga0129317_1008884	+	2786	2876	RNA→
env-206	OIYV01002562.1	+	435	526	RNA→ YitT_membrane (pfam02588)DUF2179 (pfam10035)YitT_C (cd16380)→ hypo→
env-207	scaffold82219_2_MH0077	+	234	324	RNA→ YitT (COG1284)→
env-208	Ga0134492_1011242	-	4576	4486	RNA→ YitT_membrane (pfam02588)DUF2179 (pfam10035)YitT_C (cd16380)→ hypo→
env-209	scaffold46457_3_MH0068	-	342	252	RNA→ YitT_membrane (pfam02588)→
env-210	OGGT01024343.1	+	50	140	RNA→ YitT_membrane (pfam02588)→
env-211	scaffold12407_15_MH0081	-	542	452	RNA→ YitT (COG1284)→
env-212	scaffold100726_1_MH0025	+	357	447	RNA→ YitT (COG1284)→
env-213	SRS022524_Baylor_scaffold_52019	+	65	155	RNA→
Ebi-1-1	NZ_DS996841.1	-	351476	351386	RNA→ YitT_membrane (pfam02588)DUF2179 (pfam10035)YitT_C (cd16380)→ hypo→
env-214	Pasolli2019-6816-38	+	10703	10793	RNA→ YitT_membrane (pfam02588)YitT_C (cd16380)DUF2179 (pfam10035)→ hypo→
env-215	UMGS1984-49	+	4626	4716	RNA→ YitT_membrane (pfam02588)YitT_C (cd16380)DUF2179 (pfam10035)→ hypo→
env-216	DLM008_scaffold50472_3	-	455	365	RNA→ YitT_membrane (pfam02588)→
env-217	OGLB01039490.1	+	592	682	RNA→ YitT_membrane (pfam02588)→
env-218	Ga0129315_1006925	+	545	636	RNA→ YitT (COG1284)DUF2179 (pfam10035)→
Osp-1-1	NZ_MUHW01000004.1	+	159175	159270	RNA→ Acetyltransf_1 (pfam00583)Acetyltransf_7 (pfam13508)→ hypo→
env-219	OGIG01017810.1	+	858	950	RNA→ Acetyltransf_1 (pfam00583)Acetyltransf_7 (pfam13508)→ hypo→
env-220	C3879317_1_MH0055	-	546	454	RNA→ Acetyltransf_1 (pfam00583)Acetyltransf_7 (pfam13508)→
env-221	DLM014_C710967_1	-	740	648	RNA→ Acetyltransf_1 (pfam00583)Acetyltransf_7 (pfam13508)→
env-222	SRS016954_C2819659	-	1010	918	RNA→ Acetyltransf_1 (pfam00583)Acetyltransf_7 (pfam13508)→ hypo→
env-223	SRS015065_C2411824	-	888	794	RNA→ Acetyltransf_1 (pfam00583)Acetyltransf_7 (pfam13508)→
env-224	SRS056519_LANL_scaffold_36631	-	7538	7444	RNA→ ElaA (COG2153)Acetyltransf_10 (pfam13673)→
env-225	SRS017191_C1540692	+	197	291	RNA→
env-226	SRS015578_C2534294	-	469	375	RNA→ ElaA (COG2153)→
env-227	SRS011302_C3153996	+	214	305	RNA→
env-228	SRS048164_C2262406	-	494	400	RNA→ Acetyltransf_1 (pfam00583)Acetyltransf_7 (pfam13508)→
env-229	OGMF01043907.1	+	259	353	RNA→ ←PHA00148 (PHA00148)
env-230	UMGS935-20	-	16949	16854	RNA→ Acetyltransf_1 (pfam00583)Acetyltransf_7 (pfam13508)→ hypo→
env-231	EMG_10031954	-	920	826	RNA→ Acetyltransf_1 (pfam00583)Acetyltransf_7 (pfam13508)→
Smo-1-1	NZ_KB913028.1	-	1996152	1996058	RNA→ Acetyltransf_1 (pfam00583)Acetyltransf_7 (pfam13508)→
env-232	Ga0172377_11960189	+	19	113	RNA→ ElaA (COG2153)Acetyltransf_7 (pfam13508)→
env-233	JGI2065J20421_1130138	-	106	11	RNA→
Csp-1-1	NZ_JAGI01000002.1	+	1361399	1361493	RNA→ Acetyltransf_1 (pfam00583)Acetyltransf_7 (pfam13508)→ hypo→
[ae-1-1	NZ_JHWJ01000012.1	-	85067	84973	RNA→ Acetyltransf_1 (pfam00583)Acetyltransf_7 (pfam13508)→
env-234	Ga0121585_100006	+	15719	15813	RNA→ Acetyltransf_1 (pfam00583)Acetyltransf_7 (pfam13508)→ hypo→
Cpa-1-1	NZ_ACXX02000002.1	+	203155	203249	RNA→ hypo→ Acetyltransf_1 (pfam00583)Acetyltransf_7 (pfam13508)→
					Acetyltransf_1 (pfam00583)Acetyltransf_7 (pfam13508)→ AcrR (COG1309)DHAK_reg (TIGR02366)→
					AcrR (COG1309)DHAK_reg (TIGR02366)→ hypo→ hypo→ hypo→ hypo→ hypo→
					TyrS (COG0162)PRK05912 (PRK05912)→ TyrS (COG0162)PRK05912 (PRK05912)→
Lba-2-1	NZ_LT860099.1	+	925292	925389	RNA→ Acetyltransf_1 (pfam00583)Acetyltransf_7 (pfam13508)→

env-235	DHOR01000055.1	-	5866	5770	**RNA**→ NAT_SF (cd04301)Acetyltransf_7 (pfam13508)→
env-236	Ga0121666_125164	+	61	157	**RNA**→ hypo→
env-237	Ga0121346_100024	+	6479	6575	**RNA**→ Acetyltransf_1 (pfam00583)Acetyltransf_7 (pfam13508)→
env-238	Ga0122071_102542	+	1067	1163	**RNA**→ ElaA (COG2153)MSMEG_0567_GNAT (TIGR04045)→
env-239	Ga0120789_101934	-	2022	1927	**RNA**→ Acetyltransf_1 (pfam00583)Acetyltransf_7 (pfam13508)→ hypo→
Cbe-2-1	NZ_KK211336.1	+	35574	35670	**RNA**→ Acetyltransf_1 (pfam00583)Acetyltransf_7 (pfam13508)→
Cbe-1-1	NZ_LZZC01000060.1	+	14248	14344	**RNA**→ Acetyltransf_1 (pfam00583)Acetyltransf_7 (pfam13508)→
Csp-2-1	NZ_PQCR01000003.1	-	77709	77613	**RNA**→ Acetyltransf_1 (pfam00583)Acetyltransf_7 (pfam13508)→
env-240	JXWT01081599.1	+	68	164	**RNA**→ hypo→
Bxy-1-1	NZ_PTJA01000009.1	-	140418	140322	**RNA**→ Acetyltransf_1 (pfam00583)Acetyltransf_7 (pfam13508)→ hypo→ hypo→ hypo→ hypo→
					tRNA_SAD (smart00863)AlaRS_core (cd00673)→ RimI (COG0456)Acetyltransf_7 (pfam13508)YoaP (pfam14268)→
env-241	Ga0121343_100073	+	4192	4288	**RNA**→ Acetyltransf_1 (pfam00583)Acetyltransf_7 (pfam13508)→ hypo→
env-242	Ga0120983_12029	+	243	339	**RNA**→ hypo→
Csa-2-1	NZ_ASRV01000154.1	-	1799	1703	**RNA**→ hypo→ hypo→ hypo→
Csa-1-1	NZ_CP016087.1	+	3172440	3172537	**RNA**→ Acetyltransf_1 (pfam00583)Acetyltransf_7 (pfam13508)→ hypo→
env-243	Ga0121520_108770	+	256	353	**RNA**→ Acetyltransf_1 (pfam00583)Acetyltransf_7 (pfam13508)→
env-244	Ga0122764_101145	-	1047	950	**RNA**→ Acetyltransf_1 (pfam00583)Acetyltransf_7 (pfam13508)→
env-245	Ga0122086_106318	-	1003	905	**RNA**→ Acetyltransf_1 (pfam00583)Acetyltransf_7 (pfam13508)→ Acetyltransf_10 (pfam13673)→ hypo→
env-246	Ga0121530_128175	+	153	253	**RNA**→
env-247	Ga0120789_107337	-	1207	1107	**RNA**→ Acetyltransf_1 (pfam00583)Acetyltransf_7 (pfam13508)→ hypo→
Ppr-1-1	NZ_FOTS01000031.1	+	16469	16567	**RNA**→ Acetyltransf_1 (pfam00583)Acetyltransf_7 (pfam13508)→ hypo→ hypo→
					def (PRK00150)Pep_deformylase (pfam01327)→
Psp-1-1	NZ_NFKI01000027.1	-	18466	18365	**RNA**→ hypo→
env-248	scaffold87698_2_MH0056	-	304	202	**RNA**→ ←hypo
env-249	OGCZ01005061.1	-	1450	1348	**RNA**→ PLN00175 (PLN00175)AspB (COG0436)→
env-250	Ga0134540_1011626	-	2910	2814	**RNA**→ Acetyltransf_1 (pfam00583)Acetyltransf_7 (pfam13508)→
env-251	contig39910	+	1020	1115	**RNA**→
Lba-1-1	NZ_KE159638.1	+	199563	199661	**RNA**→ Acetyltransf_1 (pfam00583)Acetyltransf_7 (pfam13508)→ hypo→ hypo→ hypo→ hypo→
env-252	scaffold50452_5_O2.UC-23	-	6721	6623	**RNA**→ Acetyltransf_1 (pfam00583)Acetyltransf_7 (pfam13508)→ hypo→
env-253	Pasolli2019-4513-9	+	120235	120333	**RNA**→ Acetyltransf_1 (pfam00583)Acetyltransf_7 (pfam13508)→
env-254	Pasolli2019-6794-4	+	66065	66164	**RNA**→ NorM (COG0534)Polysacc_synt_C (pfam14667)→
env-255	UnmappedStool_Broad_C251107655	+	74	170	**RNA**→ MSMEG_0567_GNAT (TIGR04045)→
env-256	Pasolli2019-4597-122	-	4552	4456	**RNA**→ Acetyltransf_1 (pfam00583)Acetyltransf_10 (pfam13673)→
Lsp-1-1	NZ_NFLI01000001.1	+	347559	347656	**RNA**→
env-257	Ga0134410_111722	-	924	832	**RNA**→ AspB (COG0436)PRK07324 (PRK07324)→
env-258	scaffold24592_3_MH0086	+	822	914	**RNA**→ AspB (COG0436)PRK07324 (PRK07324)→
env-259	SRS020328_C2922443	-	307	215	**RNA**→ PRK07324 (PRK07324)→
env-260	SRS014613_C1460706	+	261	353	**RNA**→ PRK07324 (PRK07324)→
env-261	SRS019910_C1660490	-	185	93	**RNA**→ ←hypo
env-262	OIYE01028171.1	+	479	571	**RNA**→ PRK07324 (PRK07324)→
env-263	Ga0129307_1035944	+	414	510	**RNA**→ PRK07324 (PRK07324)AAT_like (cd00609)→
env-264	Pasolli2019-15188-6	+	1869	1966	**RNA**→ AspB (COG0436)viomycin_VioD (TIGR03947)→
env-265	Ga0134540_1351462	+	142	238	**RNA**→
env-266	Ga0134540_1081127	+	128	225	**RNA**→ PRK07324 (PRK07324)→
env-267	Ga0134523_1000084	+	207776	207873	**RNA**→ AspB (COG0436)PRK07324 (PRK07324)→ PRK06046 (PRK06046)OCDMu (COG2423)→
env-268	Pasolli2019-15193-6	+	30316	30410	**RNA**→ AspB (COG0436)viomycin_VioD (TIGR03947)→ OCDMu (COG2423)ala_DH_arch (TIGR02371)→
env-269	Ga0134389_1083156	+	165	260	**RNA**→
env-270	scaffold571_3_MH0003	-	934	839	**RNA**→ AspB (COG0436)PRK07324 (PRK07324)→
env-271	OIWU01085457.1	-	440	346	**RNA**→ PRK07324 (PRK07324)AAT_like (cd00609)→
env-272	OGDY01022617.1	+	222	316	**RNA**→ AspB (COG0436)PRK07324 (PRK07324)→
env-273	C4413838_1_MH0053	+	296	390	**RNA**→ PRK07324 (PRK07324)→
env-274	scaffold16730_2_MH0012	-	433	339	**RNA**→ PRK07324 (PRK07324)→
env-275	Ga0116649_1139585	-	168	74	**RNA**→ hypo→

env-276	SRS013951_WUGC_scaffold_51804	+	363	457	**RNA**→ PRK07324 (PRK07324)→
env-277	SRS052697_C3077239	+	76	170	**RNA**→ PRK07324 (PRK07324)→
env-278	Ga0256406_1093232	+	780	874	**RNA**→
env-279	OIZA01039030.1	+	639	733	**RNA**→ PRK07324 (PRK07324)→
env-280	OLGV01044598.1	+	303	397	**RNA**→ PRK07324 (PRK07324)→
env-281	OIXU01035279.1	-	403	309	**RNA**→ PRK07324 (PRK07324)→
env-282	OGKG01026945.1	+	568	662	**RNA**→ AspB (COG0436)PRK07324 (PRK07324)→
env-283	OIWN01066924.1	+	301	395	**RNA**→ PRK07324 (PRK07324)→
env-284	SRS016753_C1083743	-	1333	1239	**RNA**→ AspB (COG0436)PRK07324 (PRK07324)→
env-285	Pasolli2019-15253-32	+	8879	8972	**RNA**→ AAT_like (cd00609)viomycin_VioD (TIGR03947)→
env-286	OIXV01000964.1	-	20860	20767	**RNA**→ PRK07324 (PRK07324)AAT_like (cd00609)→
env-287	scaffold27329_2_MH0037	-	1921	1828	**RNA**→ PRK07324 (PRK07324)AAT_like (cd00609)→
env-288	Ga0129311_1000221	+	5279	5373	**RNA**→ PRK07324 (PRK07324)AAT_like (cd00609)→
env-289	Ga0129312_1062540	+	217	310	**RNA**→ PRK07324 (PRK07324)AAT_like (cd00609)→
env-290	SRS058770_LANL_scaffold_39642	-	4173	4082	**RNA**→ AspB (COG0436)PRK07324 (PRK07324)→ PRK06046 (PRK06046)OCDMu (COG2423)→
env-291	Ga0116646_1029352	-	1155	1064	**RNA**→ PRK07324 (PRK07324)AAT_like (cd00609)→
env-292	scaffold33312_11_MH0040	-	844	752	**RNA**→ AspB (COG0436)PRK07324 (PRK07324)→
env-293	BAAY01003274.1	+	27	119	**RNA**→ AspB (COG0436)PRK07324 (PRK07324)→
env-294	DLM008_C884989_1	+	248	339	**RNA**→ PRK07324 (PRK07324)AAT_like (cd00609)→
env-295	OGKO01011304.1	-	765	674	**RNA**→ AspB (COG0436)PRK07324 (PRK07324)→
env-296	DOM013_scaffold21690_3	-	354	263	**RNA**→ PRK07324 (PRK07324)→
env-297	Ga0117795_1007148	-	1616	1525	**RNA**→ AspB (COG0436)PRK07324 (PRK07324)→
env-298	DLF010_scaffold264_10	+	532	623	**RNA**→ AspB (COG0436)PRK07324 (PRK07324)→
env-299	scaffold9795_2_MH0020	+	349	440	**RNA**→ PRK07324 (PRK07324)→
env-300	Ga0134428_100999	+	2432	2523	**RNA**→ AspB (COG0436)PRK07324 (PRK07324)→
env-301	OIZE01000240.1	-	28648	28557	**RNA**→ AspB (COG0436)PRK07324 (PRK07324)→
env-302	OGES01001052.1	+	14760	14851	**RNA**→ AspB (COG0436)PRK07324 (PRK07324)→
env-303	Ga0116620_1068784	+	233	324	**RNA**→
env-304	DQFY01000086.1	-	13964	13873	**RNA**→ AspB (COG0436)PRK07324 (PRK07324)→
env-305	OJAR01022809.1	-	709	618	**RNA**→ PRK07324 (PRK07324)AAT_like (cd00609)→
env-306	OGDA01002138.1	-	2438	2347	**RNA**→ AspB (COG0436)PRK07324 (PRK07324)→
env-307	Ga0134371_1000430	+	4044	4135	**RNA**→ AspB (COG0436)PRK07324 (PRK07324)→
env-308	Ga0134555_1021030	-	657	566	**RNA**→ PRK07324 (PRK07324)AAT_like (cd00609)→
env-309	OGFD01013571.1	+	32	123	**RNA**→ PRK07324 (PRK07324)AAT_like (cd00609)→ AspB (COG0436)PRK07324 (PRK07324)→
Osp-2-1	NZ_JPJG01000046.1	-	29558	29467	**RNA**→ AspB (COG0436)PRK07324 (PRK07324)→
env-310	SRS015663_WUGC_scaffold_22415	-	4933	4842	**RNA**→ AspB (COG0436)PRK07324 (PRK07324)→
env-311	OGLR01010168.1	-	273	182	**RNA**→ PRK07324 (PRK07324)→
env-312	Ga0116649_1002436	+	4045	4139	**RNA**→ PRK07324 (PRK07324)→
env-313	scaffold189312_3_MH0006	+	1363	1457	**RNA**→ hypo→
env-314	OIWT01011988.1	+	1356	1451	**RNA**→ PRK07324 (PRK07324)AAT_like (cd00609)→ PRK06046 (PRK06046)OCDMu (COG2423)→
env-315	SRS011134_C5105442	-	623	529	**RNA**→ PRK07324 (PRK07324)AAT_like (cd00609)→
env-316	OIXQ01070515.1	+	362	456	**RNA**→ hypo→
env-317	DPDF01000033.1	-	10969	10875	**RNA**→ AspB (COG0436)PRK07324 (PRK07324)→ PRK06046 (PRK06046)OCDMu (COG2423)→
env-318	OIYG01038569.1	+	450	544	**RNA**→ AspB (COG0436)PRK07324 (PRK07324)→
env-319	OIZA01044421.1	-	314	220	**RNA**→ hypo→
env-320	scaffold58436_1_MH0006	-	309	215	**RNA**→ PRK07324 (PRK07324)→
env-321	C4607443_1_V1.CD-8	+	415	509	**RNA**→ hypo→
env-322	Ga0134555_1025570	-	161	67	**RNA**→
env-323	OIYO01060334.1	+	322	416	**RNA**→ hypo→
env-324	OLGF01013261.1	+	1498	1592	**RNA**→ AspB (COG0436)PRK07324 (PRK07324)→
env-325	scaffold216697_1_MH0006	+	336	430	**RNA**→ PRK07324 (PRK07324)→
env-326	SRS017103_C2633210	-	397	302	**RNA**→ PRK07324 (PRK07324)→

env-327	OGLB01011258.1	-	191	96	**RNA**→ PRK07324 (PRK07324)→
env-328	Pasolli2019-15088-9	-	3000	2905	**RNA**→ AAT_like (cd00609)viomycin_VioD (TIGR03947)→ OCDMu (COG2423)ala_DH_arch (TIGR02371)→
env-329	scaffold7920_4_MH0003	+	5161	5256	**RNA**→ AspB (COG0436)PRK07324 (PRK07324)→
env-330	Pasolli2019-15087-20	-	14154	14059	**RNA**→ AspB (COG0436)viomycin_VioD (TIGR03947)→
env-331	Ga0116650_1001392	-	2721	2627	**RNA**→ AspB (COG0436)PRK07324 (PRK07324)→ PRK06046 (PRK06046)OCDMu (COG2423)→
env-332	OIWU01026292.1	-	1594	1500	**RNA**→ PRK07324 (PRK07324)AAT_like (cd00609)→
env-333	scaffold119784_1_MH0012	+	186	280	**RNA**→ PRK07324 (PRK07324)AAT_like (cd00609)→
env-334	OIYO01074968.1	-	407	313	**RNA**→ YqgV (COG0011)PRK07324 (PRK07324)→
env-335	OIYF01076714.1	+	98	192	**RNA**→ PRK07324 (PRK07324)AAT_like (cd00609)→
env-336	OIZA01036847.1	-	667	572	**RNA**→ PRK07324 (PRK07324)→ hypo→
env-337	Ga0116616_1036127	-	544	450	**RNA**→ PRK07324 (PRK07324)AAT_like (cd00609)→
env-338	OIXH01020913.1	+	958	1051	**RNA**→ PRK07324 (PRK07324)→ AspB (COG0436)PRK07324 (PRK07324)→
env-339	Ga0116649_1013105	+	570	664	**RNA**→ AspB (COG0436)PRK07324 (PRK07324)→
env-340	OLGF01001534.1	-	20808	20714	**RNA**→ PRK07324 (PRK07324)AAT_like (cd00609)→
env-341	SRS017521_C2613139	-	379	285	**RNA**→ PRK07324 (PRK07324)→
env-342	scaffold114617_1_MH0042	+	134	227	**RNA**→ PRK07324 (PRK07324)AAT_like (cd00609)→
env-343	scaffold82849_1_MH0009	-	370	276	**RNA**→ PRK07324 (PRK07324)→
env-344	scaffold71959_1_MH0058	-	475	381	**RNA**→ PRK07324 (PRK07324)AAT_like (cd00609)→
env-345	DJPP01000136.1	+	1352	1446	**RNA**→ PRK07324 (PRK07324)AAT_like (cd00609)→ PRK06046 (PRK06046)OCDMu (COG2423)→

1.3 Conserved domains

Conserved domains found in protein-coding genes listed in Section 1.2 are shown below, with the first sentence in their description from the Conserved Domain Database (if any). Conserved domains associated with more than one G-IV-variant RNA are assigned a color, while others are shown in gray. The number in parentheses after the colored domain name is the number of occurrences in Section 1.2.

cd00609 (93) Aspartate aminotransferase family.
cd00673 (1) Alanyl-tRNA synthetase (AlaRS) class II core catalytic domain.
cd00838 (1) metallophosphatase superfamily, metallophosphatase domain.
cd01359 (1) Argininosuccinate lyase (argininosuccinase, ASAL).
cd04242 (1) AAK_G5K_ProB: Glutamate-5-kinase (G5K) catalyzes glutamate-dependent ATP cleavage; G5K transfers the terminal phosphoryl group of ATP to the gamma-carboxyl group of glutamate, in the first and controlling step of proline (and, in mammals, ornithine) biosynthesis.
cd04301 (3) N-Acyltransferase superfamily: Various enzymes that characteristically catalyze the transfer of an acyl group to a substrate.
cd05710 (1) A subgroup of the SIS domain.
cd07079 (1) Gamma-glutamyl phosphate reductase (GPR), aldehyde dehydrogenase families 18 and 19.
cd13138 (8) Subfamily of the multidrug and toxic compound extrusion (MATE)-like proteins similar to Bacillus subtilis yoeA.
cd13143 (2) Subfamily of the multidrug and toxic compound extrusion (MATE)-like proteins similar to Streptococcus aureus MepA.
cd13547 (2) Substrate binding domain of an uncharacterized ferric iron transporter, a member of the type 2 periplasmic binding fold superfamily.
cd16343 (1) Low molecular weight protein tyrosine phosphatase.
cd16380 (12) C-terminal domain of Bacillus subtilis YitT and similar protein domains.
COG0011 (1) Uncharacterized conserved protein YqgV, UPF0045/DUF77 family [Function unknown]
COG0162 (2) Tyrosyl-tRNA synthetase [Translation, ribosomal structure and biogenesis]
COG0436 (72) Aspartate/methionine/tyrosine aminotransferase [Amino acid transport and metabolism]
COG0456 (1) Ribosomal protein S18 acetylase RimI and related acetyltransferases [Translation, ribosomal structure and biogenesis]
COG0524 (6) Sugar or nucleoside kinase, ribokinase family [Carbohydrate transport and metabolism]
COG0534 (4) Na+-driven multidrug efflux pump [Defense mechanisms]
COG0697 (1) Permease of the drug/metabolite transporter (DMT) superfamily [Carbohydrate transport and metabolism, Amino acid transport and metabolism, General function prediction only]
COG1082 (3) Sugar phosphate isomerase/epimerase [Carbohydrate transport and metabolism]
COG1178 (1) ABC-type Fe3+ transport system, permease component [Inorganic ion transport and metabolism]
COG1234 (1) Ribonuclease BN, tRNA processing enzyme [Translation, ribosomal structure and biogenesis]
COG1284 (8) Uncharacterized membrane-anchored protein YitT, contains DUF161 and DUF2179 domains [Function unknown]
COG1285 (1) Uncharacterized membrane protein YhiD, involved in acid resistance [Function unknown]
COG1309 (2) DNA-binding transcriptional regulator, AcrR family [Transcription]
COG1476 (1) DNA-binding transcriptional regulator, XRE-family HTH domain [Transcription]

COG1840 (3) ABC-type Fe3+ transport system, periplasmic component [Inorganic ion transport and metabolism]
COG2153 (7) Predicted N-acyltransferase, GNAT family [General function prediction only]
COG2159 (3) Predicted metal-dependent hydrolase, TIM-barrel fold [General function prediction only]
COG2222 (2) Fructoselysine-6-P-deglycase FrlB and related proteins with duplicated sugar isomerase (SIS) domain [Cell wall/membrane/envelope biogenesis]
COG2316 (15) Predicted hydrolase, HD superfamily [General function prediction only]
COG2326 (4) Polyphosphate kinase 2, PPK2 family [Energy production and conversion]
COG2423 (40) Ornithine cyclodeaminase/archaeal alanine dehydrogenase, mu-crystallin family [Amino acid transport and metabolism]
COG3603 (2) Uncharacterized protein [Function unknown]
COG3842 (1) ABC-type Fe3+/spermidine/putrescine transport systems, ATPase components [Amino acid transport and metabolism]
pfam00583 (68) Acetyltransferase (GNAT) family.
pfam00892 (1) EamA-like transporter family.
pfam01261 (3) Xylose isomerase-like TIM barrel.
pfam01301 (1) Glycosyl hydrolases family 35.
pfam01327 (1) Polypeptide deformylase.
pfam01894 (3) Uncharacterized protein family UPF0047.
pfam02449 (1) Beta-galactosidase.
pfam02588 (18) Uncharacterized 5xTM membrane BCR, YitT family COG1284.
pfam03976 (3) Polyphosphate kinase 2 (PPK2).
pfam04977 (1) Septum formation initiator.
pfam06810 (1) Phage minor structural protein GP20.
pfam07690 (2) Major Facilitator Superfamily.
pfam08902 (1) Domain of unknown function (DUF1848).
pfam10035 (16) Uncharacterized protein conserved in bacteria (DUF2179).
pfam12680 (1) SnoaL-like domain.
pfam13444 (1) Acetyltransferase (GNAT) domain.
pfam13508 (68) Acetyltransferase (GNAT) domain.
pfam13673 (8) Acetyltransferase (GNAT) domain.
pfam13840 (2) ACT domain.
pfam14268 (1) YoaP-like.
pfam14667 (8) Polysaccharide biosynthesis C-terminal domain.
PHA00148 (1) lower collar protein
PLN00175 (5) aminotransferase family protein; Provisional
PRK00150 (1) peptide deformylase; Reviewed
PRK00197 (1) gamma-glutamyl phosphate reductase; Provisional
PRK00855 (1) argininosuccinate lyase; Provisional
PRK05912 (2) tyrosyl-tRNA synthetase; Validated
PRK06046 (29) alanine dehydrogenase; Validated
PRK07324 (177) transaminase; Validated
PRK07777 (2) aminotransferase; Validated
PRK08618 (1) ornithine cyclodeaminase; Validated
PRK08912 (5) hypothetical protein; Provisional
PRK09453 (1) phosphodiesterase; Provisional
PRK09575 (4) multidrug efflux pump VmrA; Reviewed
PRK09813 (4) fructoselysine 6-kinase; Provisional
PRK09856 (4) fructoselysine 3-epimerase; Provisional
PRK09977 (1) putative Mg(2+) transport ATPase; Provisional
PRK11382 (2) fructoselysine-6-P-deglycase; Provisional
PRK12314 (1) gamma-glutamyl kinase; Provisional
sd00006 (1) Tetratricopeptide repeat.
smart00226 (1) Low molecular weight phosphatase family.
smart00382 (1) ATPases associated with a variety of cellular activities.
smart00471 (15) Metal dependent phosphohydrolases with conserved 'HD' motif.
smart00530 (1) Helix-turn-helix XRE-family like proteins.
smart00633 (1) Glycosyl hydrolase family 10.
smart00849 (1) Metallo-beta-lactamase superfamily.
smart00863 (1) Threonyl and Alanyl tRNA synthetase second additional domain.
TIGR00149 (3) secondary thiamine-phosphate synthase enzyme. [Unknown function, Enzymes of unknown specificity]
TIGR00711 (1) drug resistance transporter, EmrB/QacA subfamily. [Cellular processes, Toxin production and resistance, Transport and binding proteins, Other]
TIGR00797 (2) putative efflux protein, MATE family. [Transport and binding proteins, Other]
TIGR00880 (1) Multidrug resistance protein.
TIGR00950 (1) Carboxylate/Amino Acid/Amine Transporter. [Transport and binding proteins, Amino acids, peptides and amines]
TIGR00971 (1) sulfate/thiosulfate-binding protein. [Transport and binding proteins, Anions]
TIGR01135 (1) glucosamine–fructose-6-phosphate aminotransferase (isomerizing). [Cell envelope, Biosynthesis and degradation of murein sacculus and peptidoglycan, Central intermediary metabolism, Amino sugars]
TIGR02168 (1) chromosome segregation protein SMC, common bacterial type. [Cellular processes, Cell division, DNA metabolism, Chromosome-associated proteins]
TIGR02366 (2) probable dihydroxyacetone kinase regulator.
TIGR02371 (10) alanine dehydrogenase, Archaeoglobus fulgidus type.
TIGR03025 (2) exopolysaccharide biosynthesis polyprenyl glycosylphosphotransferase.
TIGR03234 (1) hydroxypyruvate isomerase.
TIGR03261 (3) putative 2-aminoethylphosphonate ABC transporter, periplasmic 2-aminoethylphosphonate-binding protein. [Transport and binding proteins, Amino acids, peptides and amines]
TIGR03262 (1) putative 2-aminoethylphosphonate ABC transporter, permease protein. [Transport and binding proteins, Amino acids, peptides and amines]
TIGR03708 (4) polyphosphate:AMP phosphotransferase. [Central intermediary metabolism, Phosphorus compounds]
TIGR03947 (24) capreomycidine synthase. [Cellular processes, Biosynthesis of natural products]
TIGR04045 (3) putative N-acetyltransferase, MSMEG_0567 N-terminal domain family.
TIGR04379 (1) myo-inosose-2 dehydratase. [Energy metabolism, Sugars]
TIGR04382 (2) 5-dehydro-2-deoxygluconokinase. [Energy metabolism, Sugars]

1.4 Multiple-sequence alignment

Each G-IV-variant RNA is denoted by an abbreviation (like "Eco-1-1") that refers to a taxonomy given in Section 1.1. The alignment may include sequences containing the RNA motif, as well as flanking sequence. The G-IV-variant RNA itself is denoted by the line underneath marked 5′ and 3′ on either end. Nucleotides in flanking sequences (i.e., not part of the motif) are written in gray letters. Stems of predicted rho-independent transcription terminators, if any, are shaded yellow. (Note: terminator predictions have not been analyzed manually, and many are likely to be false positives. Terminator predictions are those of the RNIE software.) Nucleotides predicted to function (as DNA) as transcription-factor binding sites, if any, are shaded in green. (But note: these predictions are manually annotated, so they might be under-predicted.) Annotated start codons, if any, are shaded green. (Note: start codons are frequently misannotated, especially in environmental samples.) Nucleotides proposed to basepair as part of the consensus structure are shaded in color when they comprise Watson-Crick or G-U pairs. Otherwise they are shaded gray. Conserved stems are also indicated at the bottom of the alignment by angle brackets, where matching < and > denote base-paired columns. Below these angle brackets, the symbol "2" denotes base pairs exhibiting covariation according to the statistically well-founded R-scape method. "1" denotes base pairs exhibiting covariation according to R2R's simplistic method. "0" denotes base pairs that are not observed to mutate and "?" denotes base pairs that have a significant frequency of non-canonical nucleotides for Watson-Crick or G-U pairs (> 5%). Below these base pair annotation is the consensus sequence: "R" = "A" or "G", "Y" = "C" or "U", red nucleotides: nucleotide identity conserved more than 97% of the time, black nucleotides: 90%, gray nucleotides: 75%, red circle (•): nucleotide is present 97% of the time, black circle (•): 90%, gray circle (•): 75%, white circle (◦): 50%. All percentages of sequences just described (e.g. 97% conserved) assume that sequences have been weighted by the GSC algorithm implemented by the Infernal software package.

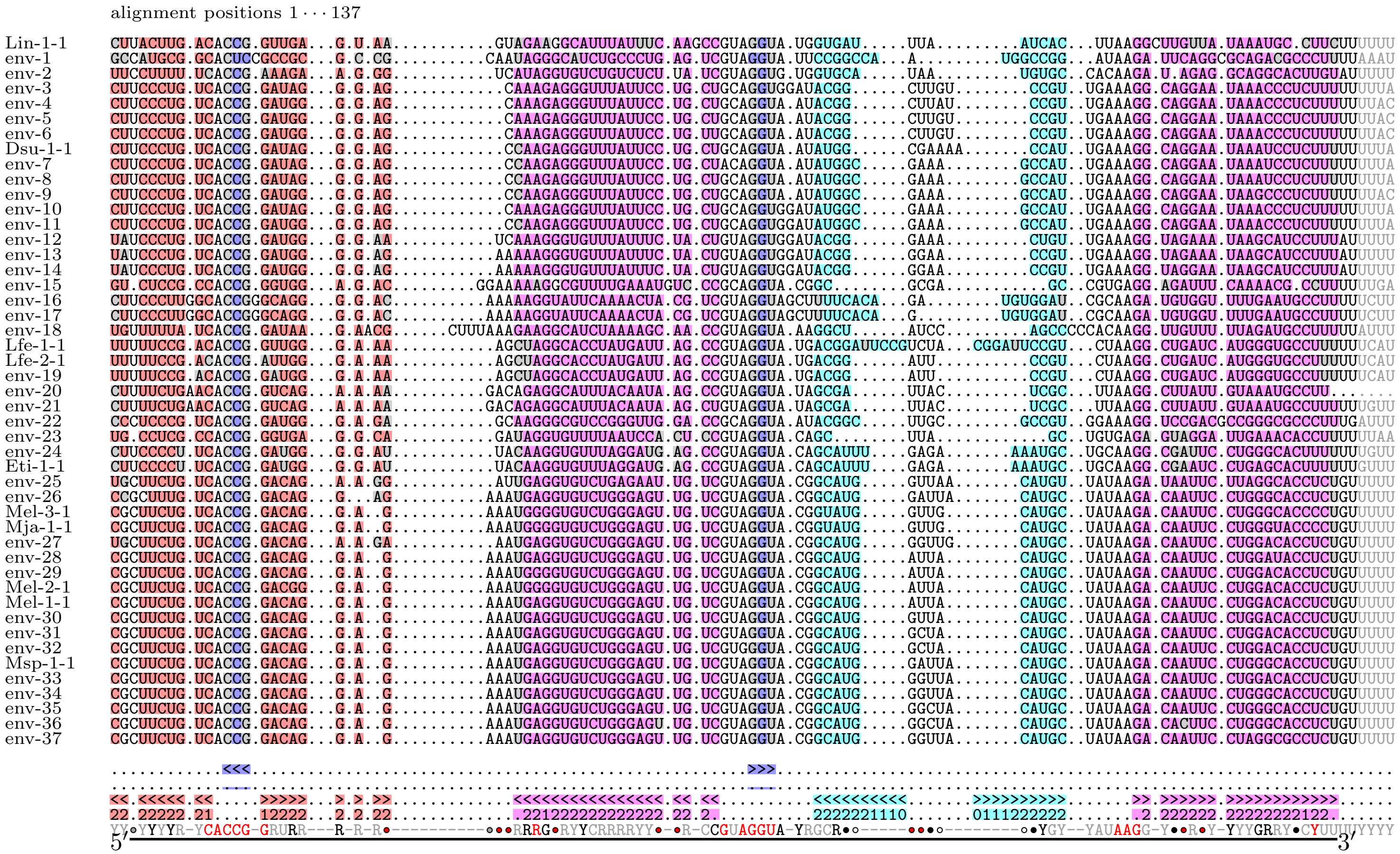

```
Cma-1-1  CGCUUCUG.UCACCG.GACAG...A.A..G.........AAAUGAGGUGUCUGGGAGU.UG.UCGUAGGUA.CGGCAUG.....GGUUG.......CAUGC..UAUAAGA.CAAUUC.CUGGACACCUCUGUUUUU
env-38   CGCUUCUG.UCACCG.GACAG...A.A..G.........AAAUGAGGUGCCUGGGAGU.UG.UCGUAGGUA.CGGCAUG.....GGUUG.......CAUGC..UAUAAGA.CAAUUC.CUGGACACCUCUGUUUUU
env-39   CGCUUCUG.UCACCG.GACAG...A.A..G.........AAAUGAGGUGUCUGGGAGU.UG.UCGUAGGUA.CGGCAUG.....GGUUG.......CAUGC..UAUAAUA.CAAUUC.CUGGACACCUCUGUUUUU
env-40   CGCUUCUG.UCACCG.GACAG...G.A..G.........AAAUGAGGUGUCUGGGAGU.UG.UCGUAGGUA.CGGCAUG.....GUUG........CAUGC..UACAAGA.CAAUUC.CUGGACACCUCUGUUUUU
env-41   CGCUUCUG.UCACCG.GACAG...G.A..G.........AAAUGAGGUGUCUGGGAGU.UG.UCGUAGGUACGGCAUG.....GUUG........CAUGC..UACAAGA.CAAUUC.CUGGACACCUCUGUUUUU
env-42   UGCUUUUG.UCACCG.GACAG...G.A.GG..........AAUCAGGUGUCUGGGAGU.UG.UCGCAGGUA.CGGCGUGC....ACG........GCAUGC..CAUAAGA.CAAUUC.CCGGGCACCUUUGUUUUU
env-43   UGCUCCUG.UCACCG.GACAG...G.A.GG...........UAAGAGGUGUCCGGGGAU.UG.CCGUAGGUA.CGGCAUGC....AAU........GCAUGC..UACAAGG.CAAUCC.CUGGGCACCUCUGUUUUU
env-44   CGCUUCUG.UCACCG.GACAG...G.A.GA..........AAUGAGGUGUCUGGGGAGU.UG.UCGUAGGUA.CGGCAUG.....AUUA........CAUGC..UAUAAGA.CAAUU..CCUGGACACCUCUGUUUU
env-45   CGCUUCUG.UCACCG.GACAG...G.A.GA..........AAUGAGGUGUCUGGGGAGU.UG.UCGUAGGUA.CGGCAUG.....AUUA........CAUGC..UAUAAGA.CAAUU..CCUKGACACCUCUGUUUU
env-46   UUCUCGGA.CCACCG.GGUUU...G.A.GA..........UUAUAAGGUAUGCUACCUU.UU.CCGUAGGUA.CGGCU.......GGAA..........AGU..UAUAAGG.AAAAGG.UGGUGUACCUUUUUGUUU
Lva-1-1  AU.UUCUA.UCACCG.GGUAG...A.A.AU........UACUUUGGGCAUCUGUACUGU.AAACCGCAGGUA.CGGCGU......GUACGA.......GCGC..CAUAAGGGUUACAG.UAUGGGUGCUUUUGUAUU
env-47   GGCCCCCA.UCACCG.GAUGGACAG.G.CC..........UUGAAGGCAUUCAGUAGAA.AA.CCGCAGGUG.UGGCGUC.....CUCG........GACGC..U.AAAGG.UUUUCU.CCUGAAUGCCUUUUUUUG
env-48   UCCGCCCG.UCGCCG.GAUAG...G.C.AG........AGCCGGAAGCAUCCAGAAACA.UU.CCGUAGGUA.UGGCGU......UUC.........GCGC..CAUAAGG.AAUGUU.UUCGGAUGCUUUUAUUUU
env-49   AUUACGGU.UCACCG.GAACG...G.U.AA.........UAUAAAGGCAUCUAGACCUA.GG.UCGUAGGUA.CGGCGC......GAAA........GCGU..UAUAAGA.CUUGGG.UCUGGAUGCUUAUUUUUU
[am-1-1  CU.GCGGU.UCACCG.GAACA...G.C.AG.........CAUGAUGGCAUCCGACCCCC.GGAUCGCAGGUA.CGGCGU......GUAU........GCGC..UAUAAGAUUCGUG..GACGGAUGCCUGUUUUUU
env-50   CU.GCGGU.UCACCG.GAACA...G.C.AG.........CAUGAUGGCAUCCGACCCCG.GA.UCGCAGGUA.CGGCGU......GUAU........GCGC..UAUAAGA.UUCGUG.GACGGAUGCCUGUUUUUU
env-51   CUCCCCCU.UCACCG.GAUGG...G.G.GA..........AUCAAGGCAUCCAAACCAA.UC.ACGUAGGUA.UGCGGU......CCAG........GCCG..UUCAAG..GAUUGG.UUUGGAUGCCUUUUUCUU
Bru-1-1  CUCCCCCU.UCACCG.GAUGG...G.G.GA..........AUCAAGGCAUCCAAACCAA.UC.ACGUAGGUA.UGCGGC......CCAA........GCCG..UUCAAG..GAUUGG.UUUGGAUGCCUUUUUCUU
env-52   CG.CUUCG.UCACCG.GAUGA...A.G.CG......GGUUUUCAGGCAUCCGGAAACU.GG.CCGCAGGUG.UAGCAUG.....UUU........UGUGC..UAUAAGG.UCAGUUUCAUGGGUGCCUUUUUUAA
env-53   CG.CUUCG.UCACCG.GAUGA...A.G.CG......GGUUUUCAGGCAUCCGGAAACU.GG.CCGCAGGUG.UAGCAUG.....UUU........CAUGC..UAUAAGG.UCAGUUUCAUGGGUGCCUUUUUUAA
env-54   UUUCUCCA.UCACCG.GGUGG...A.G.AG........CAGAAAGGGGACCGGGGACU.CG.CCGCAGGUA.CGGCAC......GCAA.........GUGU..UAUAAGG.C.AGCC.CCGGGUCCCUUUUUAAG
env-55   UUUCUCCA.UCACCG.GGUGG...A.G.AG........CAGAUAGGGGACCGGGGACU.CG.CCGCAGGUA.CGGCAC......GCAA.........GUGU..UAUAAGG.C.AGCC.CCGGGUCCCCUUUUUAA
Lsi-1-1  UUGGUAUG.UCACCG.GAUAU...A.U.UCAACUAUAAGCACAAUGGUAUUUGUUAUUC.UACCCGUAGGUA.CGGCGCC.....GCAU........GGCGU..UACAAGGGUUGAAU.GAUGAAUACCUUUUUCU
Lpe-1-1  UCGGUAUG.UCACCG.GAUAU...A.C.CAGACUAUAAGAAUGAAGGUAUUCAUUAUUC.UACCCGUAGGUA.CGGCGC......CGUAAA.......GCGU..UACAAGGGUUGAAU.GAUGGAUACCUUUUUCU
env-56   UCGAUAUG.UCACCG.GAUAU...A.U.UAGACUAUAAGAGUGAUGGUAUUCAUUAUUC.UACCCGUAGGUA.CGGCGCU.....GUAA........AGCGU..UACAAGGGUUGAAU.GGUGGAU.ACCUUUUUUC
Lsi-2-1  UCGAUAUG.UCACCG.GAUAU...A.U.CAGACUAUAAGAGUGAUGGUAUUCAUUAUUC.UACCCGUAGGUA.CGGCGCU.....GCAA........AGCGU..UACAAGGGUUGAAU.GGUGGAU.ACCUUUUUUC
Lsi-3-1  GA.UUAUA.UCACCG.GGUAU...A.A.UCAACUUUAAGAUGGAUGGUAUUCAUUGUCU.UACUCGUAGGUA.CGGCGCA.....UU..........UGUGU..UAUAAGAGUAGGAC.GAUGGAU.ACCAUUUUUU
env-57   UGGUCUCA.CCACCG.GGUGG...G.G.CG..........GCGCUGGGCCUCCGGGCUC.UG.CCGUAGGUA.CGGCACAUGCA.UUC.....UGCGUGUGC..UACAAGG.CGGAGC.UUGGGGGCCUUUAUUGUU
env-58   UGGUCUCA.CCACCG.GGUGA...G.G.CG..........GCGCUGGGCCUCCGGGCUC.UG.CCGUAGGUA.CGGCACAUGCA.UUC.....UGCGUGUGC..UACAAGG.CGGAGC.UUGGGGGCCUUUAUUGUU
env-59   AU.UCUGA.UCGCCG.GAUCG...G.A.AU.........UACAAGGGCACUCAAGACUU.UA.CCGUAGGUA.UGACAUGCC...GCU.......GGCCUGU..CACAAGG.UAAAGU.CCUGGGGGCCCUUUUUUU
env-60   CUCCUUCA.UCACCG.GGUGA...G.G.GA..........AGAAAGGCGUCUGGAUCUCUUG.CCGUAGGUA.CGGCGCGUC...UGCG.....GAUGCGC..UAUAAGG.CGGA..AUCCGGGCGCCUUUCGUCG
env-61   CUCCUUCA.UCACCG.GGUGA...A.G.GG..........UUCAAGGCGUCCGACCCCA.GU.CCGUAGGUA.CGGCGCGUU...UUC.......AACGCGU..UAUAAGG.C.AGGGUCUCGGACGCCUUUUUCU
env-62   CUCCUUCA.UCACCG.GGUGA...A.G.GG..........CUCAAGGCGUCCGACCCCA.GU.CCGUAGGUA.CGGCGCGUU...UUC.......AACGCGU..UAUAAGG.C.AGGGUCUCGGACGCCUUUUUCU
env-63   CUCCCCCU.UCACCG.GAUGG...G.G.GA..........UCAAAGGUGCCCG.GUCCC.AA.CCGUAGGUA.CGGCGC......GCAU.........GCGC..UAUAAGG.GAGGGAUCACGGGCACCUUUUUGCA
env-64   CCCCCCCU.UCACCG.GAUGG...G.G.GA..........AUCAAGGUGUCCGAUCCCC.GA.CCAUAGGUA.CGGCGC......UCAC.........GCGC..UAUAAGG.CAGGGU.CUCGGGCACCUUUUGUCU
env-65   CCCCCCCU.UCACCG.GAUGG...G.G.GA..........AUCAAGGUGUCCGAUCCCC.GA.CCAUAGGUA.CGGCGU......UCAC.........GCGC..UAUAAGG.CAGGGU.CUCGGGCACCUUUUGUCU
env-66   UCCCCCCU.UCACCG.GAUGG...G.G.GA..........AUCAAGGUGUCCGA.UCCC.CGACCGUAGGUA.CGGUGCG.....CACG........CGCGC..CAUAAGG.CGGGGAUCUCGGGCACCUUUUUUGC
env-67   CUCCCCCU.UCACCG.GAUGG...G.G.GA..........AUCAAGGUGUCCGGCCCCC.GA.CCGUAGGUA.CGGCGCU.....CACGC.......AGCGC..UAUAAGG.CGAGGGUCCCGGGCACCUUUUUGCA
Cph-1-1  CUCCCCCU.UCACCG.GAUGG...G.G.GA..........AUCAAGGUGUCCGGCCCCC.GA.CCGUAGGUA.CGGCGCU.....CGCGC.......AGCGC..UAUAAGG.CAGGGGUCCCGGGCACCUUUUUGCA
env-68   CUCCCCCU.UCACCG.GAUGG...G.G.GA..........AUCAAGGUGUCCGACCCCC.GA.CCGUAGGUA.CGGCGC......ACGC.........GCGC..CAUAAGG.CGGGGGUCUCGGGCACCUUUUUUGC
Cin-1-1  CUCCCCCU.UCACCG.GAUGG...G.G.GA..........AUCAAGGUGUCCGACCCCC.GA.CCGUAGGUA.CGGUGCG.....CACG........CGCGC..CAUAAGG.CGGGGGUCUCGGGCACCUUUUUUGC
env-69   CUCCCCCU.UCACCG.GAUGG...G.G.GA..........AUCAAGGUGUCCGACCCCC.GA.CCGUAGGUA.CGGUACG.....CACG........CGCGC..CAUAAGG.CGGGGGUCUCGGGCACCUUUUUUGC
env-70   CUCCCCCU.UCACCG.GAUGG...G.G.GA..........UCCAAGGCGUCCGAUCCCC.GA.CCGUAGGUA.CGGCGC......GCUCGCA......GCGC..UAUAAGG.CAGGGGUCUCGGGCGCCUUUUUGUC
env-71   CUCCUUCA.UCACCG.GGUGA...A.G.GA...........AGAAAGGCGUCUGAACUC.UG.CCGUAGGUA.CGGCGCUUC...UCUCC.....GAGGCGC..UAUAAGG.UCGGGU.UCGGACGCCUUUCGUGUU
env-72   CUCCUUCA.UCACCG.GGUGA...G.G.GA...........AGAAAGGCGUCUGAAUUC.UG.CCGUAGGUA.CGGCGUCC....GCUCG.....GGGCGC..UAUAAGG.CGGGGU.UCGGGCGCCUUUCGCGUU
env-73   CUUCCGCA.UCACCG.GGUGC...G.G.GC........UUUGAAGGUCGUCCGGGCAA.UU.CCGCAGGUA.CGGCGC......ACAC.........GCGC..UAUAAGG.AAUGCC.CCGGGCGUCCUUUUGCUU
env-74   CUUCCGCA.UCACCG.GGUGC...G.G.GU..........CAAAAAGACGUCCGGGCAA.UU.CCGCAGGUA.CGGCGCA.....CA..........UGCGC..UACAAGG.AAU.GC.CCGGACGCCUUUUGCUUU
env-75   CUUCCGCA.UCACCG.GGUGC...G.G.GU..........CAAAAAGACGUCCGGGCAA.UU.CCGCAGGUA.CGGCGCA.....CA..........UGCGC..UACAAGG.AAUGCC.CCGGACGCCUUUUGCUUU
env-76   CUUCCGCA.UCACCG.GGUGC...G.G.GU..........CAAAAAGACGUCCGGGCAA.UU.CCGCAGGUA.CGGCGCA.....CA..........UGCGC..UAUAAGG.AAUGCC.CCGGACGCCUUUUGCUUU
env-77   CUUCCGCA.UCACCG.GGUGC...G.G.GU..........CAAAAAGACGUCCGGGCAA.UU.CCGUAGGUA.CGGCGCA.....CA..........UGCGC..UAUAAGG.AAUGCC.CCGGACGCCUUUUGCUUU
env-78   CUUCCGCA.UCACCG.GGUGC...G.G.GU..........CAAAAAGGUGUCCGGGCAA.UU.CCGCAGGUA.CGGCGC......GCAG.........GCGC..UAUAAGG.AAUGCC.CCGGACGCCUUUUGCUUU
env-79   CUUCCGCA.UCACCG.GGUGC...G.G.GU..........CAGAAAGGUGUCCGGGCAA.UU.CCGCAGGUA.CGGCGC......GCAG.........GCGC..UAUAAGG.AAUGCC.CCGGACACCUUUUGCUUA
env-80   CUUCCGCA.UCACCG.GGUGC...G.G.GU..........CAGAAAGGUGUCCGGGCAA.UU.CCGCAGGUA.CGGCGC......GCAG.........GCGC..UAUAAGG.AAUGCC.CCGGACGCCUUUUGCUUU
env-81   CUUCCGCA.UCACCG.GGUGC...G.G.GU..........CGAAAAGGCGUCCGGGCAA.UU.CCGCAGGUA.CGGCGC......ACAG.........GCGC..UAUAAGG.AAUGCC.CCGGGCGCCUUUUGCCUU
env-82   CUUCCGCA.UCACCG.GGUGC...G.G.GU..........CAAAAAGACGUCCGGGCAA.UU.CCGCAGGUA.CGGCGC......ACAU.........GCGC..UAUAAGG.AAUGCC.CCGGGCGCCUUUUGCUUU
env-83   CUUCCCCA.CCACCG.GGUGC...G.G.GA........GCAUAUAAGGGGUUCGGGACCCGG.CCGUAGGUA.CGGCGUGC....GCG........GCACGU..UAUAAGG.CUGGUC.CCGGACC.CCUUAUUUCA
env-84   CU.GUCUA.UCACCG.GGUAG...A.C.AG........AUCAGGGGAUAUUCGAAGCUC.CG.CCGCAGGUA.CGGUGCGC....UGU........GCGCAU..UAUAAGG.CAGAGC.UCCGGAUAUCCCUUAGAC
env-85   GU.CCCUG.UCACCG.GAUGG...G.G.GC........GUAAAAAGGUGUUUCGAGAUU.UU.CCGCAGGUA.CGGCGC......UUCG.........GCGU..UACAAGG.AAAAUC.UUGGAACACCUUUUUUUC
env-86   GU.CCCUG.UCACCG.GAUGG...G.G.GC........GUAAAAAGGUGUUUCGAGAUU.UU.CCGUAGGUA.CGGCGC......UUCG.........GCGU..UACAAGG.AAGAUU.UUGGAGCACCUUUUUUUC
env-87   GU.CCCUG.UCACCG.GAUGG...G.G.GC........GUAAAAAGGUGUUUCGAGAUU.UU.CCGUAGGUA.CGGCGC......UUCG.........GCGU..UACAAGG.AAGAUU.UUGGAGCCCCUUUUUUUC
env-88   UUGCUUAU.UGCCUG.CAUUA...A.U.AA............UAAGGCAUCUGAAAUUA.AA.UCGCAGGUA.CGGUGUAU..............AUACAC..UAUAAGA.UUUAAU.UACAGAUGUCUUUUAUUU
env-89   CG.CGCUG.UCACCG.GGCAG...U.G.UG......CGAUAAAGGGCAUUCGGAAGCU.GA.CCGCAGGUA.CGGCAC......AGUU.........GUGC..UAUAAGG.UUGGCU.UACGGAUGCUCUUUGGCA
env-90   GACCGCGG.CCACCG.GGCUG...C.G.GC..........ACCAGAGUGUUCAGGACAC.CG.CCGUAGGUA.CGGCACAUC...UCG.......GAUGUGC..UAUAAGG.CGAUGU.CUUGGGCACCUUUUUCUU
env-91   UACUGCUG.CCACCG.GGCAA...C.A.GA............CAAGGGUGUUCAGGACAU.UA.CCGUAGGUA.CGGCACAUC...AAU.......GAUCUGU..UAUAAGG.AGGUGU.CUUGGACACCUUUUCUUU
env-92   AC.CACUG.CCACCG.GGCAG...U.G.GU............CAAGGGUGCUCAGGACAU.CA.CCGUAGGUA.CGGCACAU....GAAA.......AUGUGU..UACAAGG.GGGUGU.CUUGGGCACCUUUUUCUU
env-93   AC.CACUG.CCACCG.GGCAG...U.G.GU............CAAGAGUGCUCAGGACAU.CA.CCGUAGGUA.CGGCACAU....GAAA.......AUGUGU..UACAAGG.GGGUGU.CUUGGGCACCUUUUUCUU
env-94   AC.CGCUG.CCACCG.GGCAG...U.A.GU............CAAGGGUGCUCAGGACAU.CA.CCGUAGGUA.CGGCACAU....GAAA.......AUGUGU..UACAAGG.GGGUGU.CUUGGGCGCCUUUUUCUU
env-95   AU.CGUUG.CCACCG.GGCAG...C.G.AU......UAAAUGAAAGGUGUUCAGGACAC.CG.CCGUAGGUA.CAGCGCAUU...AAA.......AAUGUGC..UAUAAGG.CGAUGU.CUUGGAUCCUUUUUUCGU
Rfa-1-1  AU.CGUUG.CCACCG.GGCAG...C.G.AU......UAAAUGAAAGGUGUUCAGGACAC.CG.CCGUAGGUA.CAGCGCAUU...AAA.......AAUGUGC..UAUAAGG.CGAUGC.CUUGGAUACUUUUUUCUU
env-96   AU.CGUUG.CCACCG.GGCAG...C.G.AU......UAAAUGAAAGGUGUUCAGGACAC.CG.CCGUAGGUA.CAGCGCAUU...A.........AAUGUGC..UAUAAGG.CGAUGC.CUUGGAUACUUUUUUCUU
env-97   AU.CGUUG.CCACCG.GGCAG...C.G.AU......UAAAUGAAAGGUGUUCAGGACAC.AG.CCGUAGGUA.CAGCGCAUU...AAA.......AAUGUGC..UAUAAGG.CGAUGC.CUUGGAUACUUUUUUCUU
env-98   AU.CGUGG.CCCCCG.GGCAG...C.G.AU......UAAAUGAAAGGUGUUCAGGACAC.CG.CCGUAGGUA.CAGCGCAUU...AAA.......AAUGUGC..UAUAAGG.CGAUGC.CUUGGAUACUUUUUUCUU
env-99   GU.CGCUG.CCACCG.GGCAG...U.G.AU...........GGAGGGUGUUCAGGACAC.CG.CCGUAGGUA.CGGCACAUG...UAA.......CAUGUGU..UAUAAGG.CGAUGC.CCUGGACACCUUUUUUUC
env-100  AGCCAUUG.UCACCG.GAUGA...U.G.GU............CAAGGGUGUUCAGGACAC.CG.CCGUAGGUA.CGGCACAUG...UAA.......CAUGUGU..UAUAAGG.CGAUGU.CUUGGACACCCUUUUAUU
env-101  CCUCCACA.UCACCG.GAUGU...G.G.GC..........ACAAAGGAGUUCAAGACCA.AA.CCGUAGGUA.CGGCAUCA....A..........UGAUGC..UACAAGG.GGAGGA.UUUGAGCUCCUUUAUUUG
env-102  GU.UUUGA.UCACCG.GAUUG...A.G.AC.........GUCAAGGGCGUUCGGAACUU.UG.CCGUAGGUA.CGGCGUGCCG..GGA.....CGGCGCGU..UAUAAGG.CAAGGU.UCUGGGCGCCUUUUCGUG
env-103  UUUCUCUG.CCACCG.GGCGG...G.G.AU..........GCAAAAGGUGUUCAAAGUUU.UA.CCGUAGGUA.CGGCGC......UGAAAU.......GCGU..UAUAAGG.UGAGAC.UUUGAGCACCUUUGUUUU
Csp-4-1  UUUAAGAU.GGACCGG........U.U..G.........UUUAAAGGGCGUUCAAAAUUU.UG.CCGUAGGUA.CGGCAUACC...GAAA......GGUAUGU..CAUAAGG.CGAAGU.UUUGAGUGCCUUUUUCUU
Csp-6-1  UUCCUCCG.CCACCG.GGCGG...A.G.GG.........UGAAAAGCGUCCACGG.UC.UC.CCGUAGGUA.CGGCGCGC....UCU........GCGCGU..UACAAGG.GAG..C.CGCGGGCGCUUUAUUUUU
env-104  CUCCUGCA.CCACCG.GGUGU...G.G.GG........UCACAGGCUGCACGAAGCUC.UG.CCGUAGGUA.CGGCGCGC....ACAG.......GCGCGC..UAUAAGG.CAGCGC.CCCGUGCGGCUUUUUCUU
env-105  UGCCUGCA.CCACCG.GGUGC...A.G.GA.........CUACAGGGCAUCUGAAGGC.UGACCGCAGGUA.CGGUAUACC...GGAA......GGUAUAU..UAUAAGG.CAGGCU.UCAGGUGUUUAUUCUUUC

         ...........<<<......................................................>>>.........................................................................
         ..........................................................................................................................................................
         <<.<<<<<.<<.....>>>>>...>.>.>>.............<<<<<<<<<<<<<<<<.<<.<<..........<<<<<<<<<<.......>>>>>>>>>>.......>>.>>>>>>.>>>>>>>>>>>>......
         22.22222.21.....12222...2.2.22..............221222222222222.22.2...........2222221110.......0111222222........2.222222.22222222122.......
         YY∘YYYYR-YCACCG-GRURR---R-R-R•----------∘••RRRG•RYYCRRRRYY•-•R-CCGUAGGUA-YRGCR•∘-----•••∘--------∘•YGY--YAUAAGG-Y••R•Y-YYYGRRY•CYUUUUYYYY
         5′                                                                                                                                     3′
```

```
Pal-1-1   CUGCGCCA.UCACCG.GGUGG...C.G.UG............UCAAGGAUUCAAAGCCU.CA.CCGCAGGUA.CGGCGC......GUUU.........GCGC..UAUAAGGG..AGGC.UUUGAAUCCUUUUUUAAA
env-106   UGCUCCCA.UCACCG.GGUGG...G.A.GA.........GUAAAAGGCGUUCAAGAUUC.UG.CCGCAGGUA.CGGUAUGCC...GGCC......GGCAUGC...CCAAGG.CAGGAU.CCUGGGCGCCUUUUUCUG
env-107   CG.UCCUA.UCACCG.GAUAG...G.G.CA.........CGAAAGGGCGUUCAUGACGC.UG.CCGUAGGUA.CGGCGCAUCGG.GUGA....CUGAUGCGU..UAUAAGG.CAGGGU.CGUGAGCGCCUUUUGCAU
env-108   UUUCCUCA.CCAUUG.GAUGG...G.G.AU........GCGAAGAGGGAUUCAAGACUU.UG.CCGUAGGUA.CGGUAUGCU...UAAGC.....AGCAUGU..UGCAAGG.CGGGGG.UUUGGGUCUUCUUCUCUU
env-109   UUUCCUCA.CCACCG.GGUAG...G.G.AU........GCGAAGAGGGAUUCAAGACUU.UG.CCGUAGGUA.CGGUAUGCU...UAAGU.....GGCAUGU..UGCAAGG.CGGGGU.UUUGGGUCUUUUUCGUUU
env-110   UUUCCUCA.CCACCG.GGUAG...G.G.AU........GCGAAGAGGGAUUCAAGACUU.UG.CCGUAGGUA.CGGUAUGCU...UAAGC.....GGCAUGU..UGCAAGG.CGGGGU.UUUGGGUCUUUUUCGUUU
env-111   CUCGUGCA.CCACCG.GGUGC...G.C.GG..........UCAAUGGGCAUCCAAAGCUUCG.CCGUAGGUA.CGGCAUAC....UCAG.......GUAUGU..UAUAAGG.CGGGCU.UUGGGUG.CCCUUUUGCU
env-112   UUACCUCA.CCACCG.GGUGG...G.G.AU.........AAAGAAGAGGUUCAGGACUU.UA.CCGUAGGUA.CGGCGUACU...GAAU......AGUGCGU..UAUAAGG.CGAAGU.CUUGAGUCUCUUUACUUU
env-113   UUGCCUCA.CCACCG.GGUGA...G.G.AU.........UAAAAAGAGGUUCAGGACUU.UA.CCGUAGGUA.CGGCGCAUU...GAAU......AGUGCGU..UAUAAGG.CAAAGU.CUUGAGUCUCUUUUCUUU
env-114   UUGCCUCA.CCACCG.GGUGG...G.G.AU.........UAAAAAGAGGUUCAGGACUU.UA.CCGUAGGUA.CGGCGCAC....GGAACA.....GUGCGU..UAUAAGG.CGAAGU.CUUGAAUCUCUUUUCUUU
Dsa-1-1   GU.CCUCA.UCACCG.GGUGA...G.G.AU.........UUUAAAGGCGUUCAUAGCUU.UG.CCGUAGGUA.CGGCACUCC...GAGA......GGUGUGC..UACAAGG.C.AGGC.UGUGAACGCCUUUUUGUU
env-115   GU.UCCCA.UCACCG.GAUGG...G.A.AC.........UUUAAAGGCGUUCAUGGUUU.UG.CCGUAGGUA.CGGCACACC...GACA......GGUGUGC..UACAAGG.CA.GGC.CGUGAACGCCUUUUUGUU
env-116   GU.UUCUG.CCACCG.GGCAG...A.A.AC.........UCAAAAGACGUUCAGAACCU.UG.CCUUUAGUA.CGGCAUACC...AGUA......GGUAUGU..UAUAAAG.CGGGGG.UUUGAACGUCUUUUAUUU
env-117   GU.UUCUG.CCACCG.GGCAG...A.A.AC.........UCAAAAGGCGUUCAAAACCU.UG.CCGUAGGUA.CGGCAUACC...AUCA......GGUAUGU..UAUAAGG.CGGGGU.UUUGAACGCCUUUAAUUU
env-118   GU.UUCUG.CCAGCG.GGCAG...A.A.AC.........GAAAAUGGCGUUCAGAACCU.UG.CCGUAAGUA.CGGCAUACC...AGCA......GGUAUGU..UAUAAGG.C.AGGUGGUUGAACGUCUUUUAUUU
env-119   GU.UUCUG.CCAUCG.GGCAG...A.A.AC.........GCAAAAGGCGUUCAGAACCU.UG.CUGUAAGUA.CGGCAUACC...AACA......GGUAUGU..UAUAAGG.CGGGGG.UUUGAACGUCUAUUAAUA
env-120   GU.UUCUG.CCACCG.GGCAG...A.A.AC.........GCAAAUGGCGUUCAGAACCU.UG.CCGUAAGUA.CGGCAUACC...AGCA......GGUAUGU..UAUAAGG.CAGGGG.UUUGAACGUCUUUUGUUU
env-121   CUUCUCCA.CCACCG.GGUGG...A.G.AU.........UAAAAGGCUAAUCAGACUUU.UG.CCGUAGGUA.CGGUAUACC...AUU.......GGUAUAU..UAUAAGG.CUUAGG.UCUGAUUAGCCUUUUUUG
Vma-1-1   UUCCUUCA.CCACCG.GGUGA...A.G.GG..........UGAUAAGCAACCGAAGCUU.UU.CCGUAGGUA.CGGCGUU.....GUU.........GACGC..UAUAAGG.AAGAGC.UGCGGUUGCUUUUUUUAU
env-122   UUCCAUCG.CCACCG.GGUGA...U.G.GA..........UCAAAAGCGCACGGGACUU.UU.CCGUAGGUA.CGGCGUG.....GAAA........CACGU..UAUAAGG.AAGAGU.CUCGGGCGUUUUAUUGUU
env-123   UUCCGUCA.CCACCG.GGUGA...U.G.GA..........UCAAAAGCGCACGGGACUU.UU.CCGUAGGUA.CGACGUG.....GAAA........CACGU..UAUAAGG.AAGAGC.CUCGGGCGUUUUAUUGUU
env-124   UUCCGUCA.CCACCG.GAUGA...U.G.GA..........UCAAAAGCGCACGGGACUU.UU.CCGUAGGUA.CGGCGUG.....GAAA........CACGU..UAUAAGG.AAGAGC.CUCGGGCGUUUUAUUGUU
env-125   UUCCGUCA.CCACCG.GGUGA...U.G.GA..........UCAAAAGCGCACGGGACUU.UU.CCGUAGGUA.CGGCGUG.....GAAA........CACGU..UAUAAGG.AAGAGC.CUCGGGCGUUUUAUUGUU
env-126   UUCCGUCA.CCACCG.GGCGA...U.G.GA..........UAAAAAGCGCACGGGACUU.UU.CCGUAGGUA.CGGCGUG.....GAAA........CACGU..UAUAAGG.AAGAGC.CUCGGGCGUUUUAUUGUU
env-127   UGCCCUUA.CCGCCG.GGCGA...A.G.GA..........UCACGGGCGUGCGUGACUU.UU.CCAUAGGUA.CGGCGCG.....GAGA........CACGC..UAUAAGG.AAUAUC.CUCGGGCGUUUUAUUGUU
env-128   UUCCCUCA.UCACCG.GGUGA...G.G.GG..........CCAAAGGCACCUGGAGCUU.UC.CCGCAGGUA.CGGCGUGC....AAGA.......GCACGU..UAUAAGG.GAGGGCUCACAGGGGCCUUUCUUUU
env-129   UUCCCUCA.CCACCG.GGUGA...G.A.GG......GCAAAAAAGGGUAUUCAAAACCU.UG.CCGUAGGUA.UAGUGUGCG...AAA.......UGCGUGC..UACAAGG.CGAUGU.UCUGAAUACCUUUUUUAC
env-130   UCCCCUCA.CCACCG.GGUGA...G.A.GG.......AUUAAUGGGGUGUUCAAAACCU.CG.CCGUAGGUA.UAGUGUGCG...AAA.......UGCGUGC..UACAAGG.CGAUGU.UCUGAAUACUUUUUUUUA
env-131   UUCCCUCA.CCACCG.GGUGA...G.A.GG.......ACUAAUAGGGUAUUCAAAACCU.CG.CCGUAGGUA.UAGUGUGCG...AAA.......UGCGCGC..UACAAGG.CGAUGU.UCUGAAUACCUUUUUUUA
env-132   UUCCCUCA.CCACCG.GGUCA...G.A.GG.......ACUAAUAGGGUAUUCAAAACCU.CG.CCGUAGGUA.UAGUGUGCG...AAA.......UGCGCGC..UACAAGG.CGAUGU.UCUGAAUACCUUUUUUUA
env-133   UUCCCUCA.CCACCG.GGUGA...G.A.GG.......GCUAAAAGGGUAUUCAAAAUUU.CG.CCGUAGGUA.UAGUGUGCA...GUA.......UGCGUGC..UACAAGG.CGAUGU.UCUGAAUGCCUUUUUUUU
env-134   UUCCCUCA.CCACCG.GGUGA...G.A.GG.......GUUAAAAGGGUAUUCAAAAUUU.UG.CCGUAGGUA.UAGUGUGCA...GUA.......UGCGUGC..UACAAGG.CGAUGU.UCUGAAUGCCUUUUUUUU
env-135   UUCCCUCA.CCACCG.GGUGA...G.A.GG.......GUUAAAAAGGUAUUCAAAACCU.CG.CCGUAGGUA.UAGCAUGCA...GGA.......UGUGUGC..UACAAGG.CGAUGU.UCUGAAUGCCUUUUUUUU
env-136   CUUCCUCA.CCACCG.GGUGU...G.G.AU..........UAAAAGGCGUGCAAGGCUU.CA.CCGUAGGUA.CGGCAUU.....UAUU........AAUGC..UAUAAGG.AGAAGC.CCUGCACGCCUAUUUAAA
env-137   CUUCCUCA.CCACCG.GGUGG...G.G.AC..........AAAAGGGCGUCCAGGACGG.AU.CCGUAGGUA.CGGCA.......GAAA..........UGC..UAUAAGG.AGCUGU.CAUGGAGGCCUUUUCUUU
env-138   CUUCCUCA.CCACCG.GGUGG...G.G.AG.........CAAAAGGGCGUCCAGGACGG.AU.CCGUAGGUA.CGGCAG......CAA..........CUGC..UAUAAGG.AACCGC.CGUGGAGGCCUUUUCUUU
env-139   UUGUUCCA.CCACCG.GGUGG...A.A.UG.........UCAAAAGGUAUUCAGAACUU.UU.CCGUAGGUA.CGGCAUAG....GAU........UUAUGU..UAUAAGG.AAUGGU.UCUGAAUACCUUUUGUUU
env-140   UUACCCCA.UCACCG.GGUGG...G.G.AG..........UUUGAAGCAUCCAGAGCUU.UC.CCGUAGGUA.CGGCGUGCG...CUG.......CGCACGU..UAUAAGG.GAUGGC.UCUGGAUGCUUUCGCUUU
env-141   UUACCCCA.UCACCG.GGUGG...G.G.AA..........UUUGAAGCAUUCAGAGCUU.UC.CCGUAGGUA.CGGCGUGCG...CUG.......CGCACGU..UAUAAGG.GAUGGC.UCUGGAUGCUUUCGCUUU
env-142   CCUCUUCA.CCACCG.GGUGG...A.G.AU.........ACCAAGGGCAUACAGCGUUU.UU.CCGUAGGUA.CGGCAUGG....AUU........CCAUGU..UAUAAGG.AAAAAC.GCUGUAUGUCCUUUCUUU
env-143   CUUCUCCA.CCACCG.GGUGG...A.G.GC..........CAAAGGGCAUACAGAGUUU.UU.CCGUAGGUA.CGGCAUGG....UUCA.......CCAUGU..UAUAAGG.AAAAGC.UCUGUGUGCCCUUUUCUU
env-144   CCUCUCCA.CCACCG.GGUGG...A.G.GC..........CAAAGGGCACACAGAGUUU.UU.CCGUAGGUA.CGGCAUGG....UUCA.......CCAUGU..UAUAAGG.AAAAGC.UCUGUGUUCCUUUUCUU
env-145   CCUCUCCA.CCACCG.GGUGG...A.G.GC..........CAAAGGGCACACAGAGUUU.UU.CCGUAGGGA.CGGCAUGG....UUCA.......CCAUGU..UAUAAGG.AAAAGC.UCUGUGUGCCCUUUUCUU
env-146   CCUCUCCA.CCACCG.GGUGG...A.G.GC..........CAAAGGGAACACAGAGUUU.UU.CCGUAGGUA.CGGCAUGG....UUCA.......CCAUGU..UAUAAGG.AAAAGC.UCUGUGUGCCCUUUUCUU
env-147   CCUCUCCA.CCACUG.GGUGG...A.G.GC..........CAAAGGGCACACAGAGUUU.UU.CCGUAGGUA.CGGCAUGG....UUCA.......CCAUGU..UAUAAGG.AAAAGC.UCUGUGUGCCCUUUUCUU
env-148   UCUCUCCA.CCACCG.GGUGG...A.G.GC..........CAAAGGGCACACAGAGUUU.UU.CCGUAGGUA.CGGCAUGG....UUCA.......CCAUGU..UAUAAGG.AAAAGC.UCUGUGUGCCCUUUUCUU
env-149   CCUCUCCA.CCACCG.GGUGG...A.G.GC..........UAAAGGGCACACAGAGUUU.UU.CCGUAGGUA.CGGCAUGG....UUCA.......CCAUGU..UAUAAGG.AAAAGC.UCUGUGUGCCCUUUUCUU
env-150   CCUCUCCA.CCACCG.GGUGG...A.G.GU..........CAAAGGGCACACAGAGUUU.UU.CCGUAGGUA.CGGCAUGG....UUCA.......CCAUGU..UAUAAGG.AAAAGC.UCUGUGUGCCCUUUUCUU
env-151   CCUCUCCA.CCACCG.GGUGG...A.G.GC..........CAAAAGGCACACAGAGUUU.UU.CCGUAGGUA.CGGCAUGG....UUCA.......CCAUGU..UAUAAGG.AAAAGC.UCUGUGUGCCCUUUUCUU
env-152   CCUCUCCA.CCACCG.GGUGG...A.G.GC..........CAAAGGGCACACAGAGUUU.UU.CCGUAGGUA.CGGCAUGG....UUCA.......CCAUGU..UAUAAGG.AAAAGC.UCUGUGUGCCCUUUUCUU
env-153   CCUCUCCA.CCACCG.GGUGG...A.G.GC..........CAAAGGGCACACAGAGUUU.UU.CCGUAGGUA.CGGCAUGGU...UC........ACCAGGU..UAUAAGG.AAAAGC.UCUGUGUGCCUUUUUCUU
env-154   CCUCUCCA.CCCCCG.GGUGG...A.G.GC..........CAAAGGGCACACAGAGUUU.UU.CCGUAGGUA.CGGCAUGGU...UC........ACCAUGU..UAUAAGG.AAAAGC.UCUGUGUGCCCUUUUCUU
env-155   CCCCUCCA.UCACCG.GGUGG...A.G.GA.........CAAAGGGACACACAGAGUUU.UU.CCGUAGGUA.CGGCAUGGA...UCG.......UCUAUGC..UAUAAGG.AAAAGC.UAUGUGUGUUUUUUCUUU
env-156   UUUCCUCA.CCACCG.GGUGG...G.G.AU..........GAAAAGGCAUUCGGAGCAU.UU.CCGUAGGUA.CGGCGU......UUUAU........ACGU..UAUAAGG.AAAGGC.ACUGAAUGCCGUUUACUU
env-157   CUCCCUUA.CCACCG.GGUAG...G.G.AU..........UAGAAGGUAUUCAGAGUUU.UG.CCGUAGGUA.CGGCAUAUC...GCAA......GAUAUGU..UAUAAGG.CAGGAC.UCUGGAUACCUUUUCUUU
env-158   UC.CCUUA.CCACCG.GGUAG...G.G.GU..........UAGAAGGUAUUCAGAGUUU.UG.CCGUAGGUA.CGGCAUAUC...GCAA......GAUAUGU..UAUAAGG.CAGGAC.UCUGGAUACCUUUUCUUU
env-159   CUCCCGCA.CCACCG.GGUGC...G.G.GG..........CAAAAGGUGUUCAAAACCU.UG.CCGUAGGUA.CGGCAUGCCG..GA.......CGGUAUGC..UAUAAGG.CAGGGU.UUUGAGCAUCUUUUCUUU
env-160   UAUUUCCA.CCACCG.GGUGG...A.A.UG.........UGAAAGGGCAUUUAAACCAC.UU.CCGUAGGUA.CGGCAUACCGA.UGA.....UUGGUAUGU..UAUAAGG.CAGUGG.UUUGGAUGUCCUUUUAAU
env-161   UAUUUCCA.UCACCG.GAUGG...A.A.UG.........UUAAGGGGCAUCCGAACCAU.UG.CCGUAGGUA.CGGCAUACC...GAUU......GGUAUGU..UAUAAGG.CAGUGG.UUCGGAUGCCCUUUUGCU
env-162   UAUUUCCA.UCACCG.GGUGG...A.A.UG.........UUAAGGGGCAUCCGAACCAU.UG.CUGUAGGUA.CGGCAUACC...GAUU......GGUAUGU..UAUAAGG.CAGUGG.UUCGGAUGCCCUUUUGCU
env-163   UAUUUCCA.UCACCG.GGUGG...A.A.UG.........UUAAGGGGCAUCCGAACCAU.UG.CCGUAGGUA.CGGCAUACC...GAUU......GGUAUGU..UAUAAGG.CAGUGG.UUCGGAUGCCCUUUUGCU
env-164   CGUUCUCA.CCACCG.GGUGG...G.A.UG..........UCAAGGGCACUCAAAGCUU.UG.CCGUAGGUA.CGGCACGCC...GCA.......GGCGUGU..UAUAAGG.CGGGGC.UUUGGGUGCCCUUUUUCU
env-165   CUCCUGCA.UCACCG.GGUGC...A.G.GG.........CAAAAGGGCAUCCGGAGUUU.UG.CCGUAGGUA.CGGUAUACC...GUGA......GGUAUAU..UAUAAGG.CAGGAC.UCCGGGUGCCCUUUCCAU
env-166   CUCCCGCA.CCACCG.GGUGC...A.G.GG........UUCAAAGGGCAUCCGAAACAU.CG.CCGUAGGUA.CGGCAUACU...GUC.......AGUAUGU..UAUAAGG.CGGAGU.UUCGGAUGCCCUUUUCCG
env-167   UC.UCACA.CCACCG.GGUGU...G.A.GA.........UCCAAGGGCAUCCAGAGCUU.CG.CCGUAGGUA.CGGCAUACC...GGUU......GGUAUGU..UACAAGG.CAGAGC.UUUGGAUGCCCUUUCCAA
env-168   CUCUCACA.CCACCG.GGUGU...G.A.GA.........CCCAAGGGCAUCCAAAGCUU.GG.CCGUAGGUA.CGGCAUACC...GGCA......GGUAUGU..UACAAGG.CGGAGC.UUUGGAUGCCCUUUCAAA
env-169   CUCUCACA.CCACCG.GGUGU...G.A.GA.........CCCAAGGGCAUCCAGAGCUU.GG.CCGUAGGUA.CGGCAUACC...GGCA......GGUAUGU..UACAAGG.CGGAGC.UUUGGAUGCCCUUUCAAA
env-170   CUCUCGCA.CCACCG.GGUGU...G.A.GA.........UCCAAGGGCAUCCAGAGCUU.CG.CCGUAGGUA.CGGCAUACC...GGCA......GGUAUGU..UAUAAGG.CAGAGC.UUUGGAUGCCCUUUCAAA
env-171   UC.UCACA.CCACCG.GGUGC...G.A.GA.........UCCAAGGGCAUCCAGAGCUU.GG.CCGUAGGUA.CGGCAUACC...GGCA......GGUAUGU..UACAAGG.CGGAGC.UCUGGAUGCCCUUUCCAA
env-172   UC.UCACA.CCACCG.GGUGC...G.A.GA.........UUCAAGGGCAUCCAGAGCUU.GG.CCGUAGGUA.CGGCAUACC...GGCA......GGUAUGU..UACAAGG.CGGAGC.UUUGGAUGCCCUUUUCAA
env-173   UC.UCACA.CCACCG.GGUGC...G.A.GA.........UCCAAGGGCAUCCAGAGCUU.GG.CCGUAGGUA.CGGCAUACC...GGCA......GGUAUGU..UAUAAGG.CGGAGC.UUUGGAUGCCCUUUCAAA
env-174   UC.UCACA.CCACCG.GGUGG...G.A.GA.........UUCAAGGGCAUCCGGAGCUU.GG.CCGUAGGUA.CGGCAUACC...GGCA......GGUAUGU..UAUAAGG.CGGAGC.UCUGGAUGCCCUUUCAAA
env-175   UC.UCACA.CCACCG.GGUGG...G.A.GA.........UUCAAGGGCAUUCAGAGCUU.GG.CCGUAGGUA.CGGCAUACC...GGCA......GGUAUGU..UACAAGG.CGGAGC.UCUGGAUGCCCUUUCAAA
env-176   CUCUCACA.CCACCG.GGUGG...G.A.GA.........UGCAAGGGCAUUCAGAGCUU.GG.CCGUAGGUA.CGGCAUACC...GGCA......GGUAUGU..UAUAAGG.CGGAGC.UCUGGAUGCCCUUUCAAA
env-177   CGCCUGCA.CCACCG.GGUGC...G.G.GA........UCAGAAGGGCAUCCAAAACUU.CA.CCGUAGGUA.CGGCAUACC...GGAA......GGUAUGU..UAUAAGG.CGGAGU.UUUGGAUGCCCUUUUCAA
env-178   UC.CUGCA.CCACCG.GGUGC...G.G.GA........UCAGAAGGGCAUCCAAAACUU.CA.CCGUAGGUA.CGGCAUACC...GGAA......GGUAUGU..UAUAAGG.CGGAGU.UUUGGAUGCCCUUUUCAA
env-179   CGCCUGCA.CCACCG.GGUGC...G.G.GA........UCAAAAGGGCAUCCAGAGCUU.UU.CCGUAGGUA.CGGCGUACC...GAAA......GGUAUGU..UAUAAGG.AAAGGC.UUUGGAUGCCCUUUCAAA
env-180   CGCCUGCA.CCACCG.GGUGC...G.G.GA........UCAAAAGGGCAUCCAGAGCUU.UU.CCGUAGGUA.CGGCGUACC...GAAA......GGUAUGU..UAUAAGG.AAAGGC.UUUGGAGGCCCUUUCAAA
env-181   CUCCCGCA.CCACCG.GGUGC...G.G.GA........UCAACAGGGCAUCCAAAGCUC.CG.CCGUAGGUA.CGGCAUACC...GAGG......GGUAUGU..UAUAAGG.CGGUGC.UUUGGACGCCCUGUUCCA
env-182   UUCCCGCA.CCACCG.GGUGC...G.G.GA........CUAAAAGGGCGUCCAAAACCC.CA.CCGUAGGUA.CGGCAUGCC...GGAA......GGCGUGU..UAUAAGG.CGGGGC.UUUGGAUGCCUUUUUCGA
env-183   UC.CUGCA.CCACCG.GGUGC...G.G.GA.........UCCAAGGGCAUCCGAAGCUU.CG.CCGCAGGUA.CGGUAUACC...GUUU......GGUAUAU..UAUAAGG.CGCUGC.UUUGGAUGCCUUUUCAAG

          ............<<<.....................................................>>>..................................................................
          ............---.....................................................---..................................................................
          <<.<<<<<.<<.....>>>>>...>.>.>>............<<<<<<<<<<<<<<<<<.<<.<<..........<<<<<<<<<<.......>>>>>>>>>>.......>>.>>>>>>.>>>>>>>>>>>>......
          22.22222.21.....12222...2.2.22.............2212222222222222.22.2...........2222221110.......0111222222........2.222222.22222222122.......
          YY∘YYYYR-YCACCG-GRURR---R-R-R•----------∘••RRRG•RYYCRRRRYY•-•R-CCGUAGGUA-YRGCR•∘-----•••∘--------∘•YGY--YAUAAGG-Y••R•Y-YYYGRRY•CYUUUUYYYY
          5′                                                                                                                                     3′
```

```
env-184   CUCGCACA.CCACCG.GGUGU...G.C.GG.........UCAACGGGCAUCCAAAACCC.CG.CCGUAGGUA.CGGCGCG.....CACAGU......CGCGC..UAUAAGG.CAGGGU.UUUGGGUGCCCGUUUCCA
env-185   CUCGCGCA.CCACCG.GGUGC...G.C.GG.........UCAACGGGCAUCCAAAACUC.CG.CCGUAGGUA.CGGCGCGC....UUG........GCGCGU..UAUAAGG.CAGGGU.CUUGGAUGCCCGUUUCGC
env-186   CUCGCGCG.CCACCG.GGUGC...G.C.GG.........UCAACGGGCAUCCAAAACUC.CG.CCGUAGGUA.CGGCGCGC....UUA........GCGCGU..UAUAAGG.CAGGGU.CUUGGAUGCCCGUUUCGC
env-187   UUUCUCCA.CCACCG.GGUGG...A.G.UG..........UAAAGGACAUUCAGAGCUUAUG.CCGUAGGUA.CGGCGC......GUUCA........GCGU..UAUAAGG.CAGAGC.UUUGGAUGUCCUUUCGUU
env-188   UUUCUCCA.CCACCG.GGUGG...A.G.GG..........UAAAGGACAUUCAGAGCUUAUG.CCGUAGGUA.CGGCGC......GUUCA........GCGU..UAUAAGG.CAGAGC.UUUGGAUGUCCUUUCGUU
env-189   GU.UUUCA.UCACCG.GGUGA...A.A.GC..........AAAAAGGCAUCCCUAAUUG.AG.CCGCAGGUA.CGGUGUA.....GCAA........UACAC..UAUAAGG.UUCAAU.UACGGGUGCCUUUUUUUG
env-190   GU.UUUCA.UCACCG.GGUGA...A.A.AU...........GAAAAAGCAUCUUAAUUG.AG.CCGUAGGUA.CAGUGUA.....GCAA........UACAC..CACAAGG.UUCAAU.UGGGAUGCUUUGUUUUUU
Csp-3-1   UG.CUUUA.UCACCG.GGUAA...A.G.CG.......AAUUUUAAGGCAUAUAAAAAUC.GA.CCGCAGGUA.CGGCGCC.....GAAU........GGCGU..UAUAAGG.UUGGUU.UUUAUAUGCCUUUAUUUU
env-191   AAACACCA.UCACCG.GAUAG...U.G.UG.....UGUAUUUCAAGGCAUUCAAAAGUU.AG.CUGCAGGUA.CGGUGCA.....GUU.........UGCAC..UAUAAGG.CUGGCU.UUUGGAUGCCUUUUGCUU
env-192   GU.CUUUG.UCACCG.GAUGA...A.G.GC........ACUUCAAGGCACUCAUAAUCA.AA.CCGUAGGUA.CGGUGCA.....GCAU........UGCAC..CAUAAGG.UUUGGU.UCUGGGUGCCUUUUUUUU
Csp-5-1   UUCAUUUA.UCACCG.GAUAA...A.U.GA..........UUCAAAGCAUCCGGGAUUC.AA.UCGCAGGUA.CGGCAU......GGAU.........AUGC..UAUAAGA.UUGGAU.CCUGGGUGCUUUUUUGCG
env-193   UUCAUUUA.UCACCG.GAUAA...A.U.GA..........UUCAAAGCAUCCGGGAUUC.AA.UCGCAGGUA.CGGCAU......GGAU.........AUGC..UAUAAGA.UUGGAU.CUUGGGUGCUUUUUU...
env-194   UUCACUCU.UCACCG.GAUGU...G.U.GG...........UUAAAGCUUCUGGGAGCA.AA.UCGUAGGUG.CAGU........AAUUUAC........AC..UGUAAGA.UUUGUU.UCCAGACGCUUAUUUAUU
[re-1-1   UUCACUCU.UCACCG.GAUGA...G.U.GG...........UUAAAGCGUCUGGGAGCA.AA.UCGUAGGUG.CAGU........AAUUUAC........AC..UGUAAGA.UUUGUU.UCCGGACGCUUAUUUAUU
env-195   UUCACUCU.UCACCG.GAUGA...G.U.GG...........UUAAAGCGUCUGGGAGCA.AA.UCGUAGGUG.CAGU........AAUUUAC........AC..UGUAAGA.UUUGUU.UCCAGACGCUUAUUUAUU
env-196   UUCACUCU.UCACCG.GAUGA...G.U.GG...........UUAAAGCGUCUGGGAACA.AA.UCGUAGGUG.CAGU........AAUUUAC........AC..UGUAAGA.UUUGUU.UCCGGGCGCUUAUUUAUU
env-197   UUCACUCU.UCACCG.GAUGA...G.U.GG...........UUAAAGCGUCUGGGAGCA.AA.UCGUAGGUG.CAGU........AAUUUAC........AC..UGUAAGA.UUUGUU.UCCAGGCGCUUAUUUAUU
env-198   UUCACUCU.UCACCG.GAUGA...G.U.GG...........UUAAAGCGUCUGGGAGCA.AA.UCGUAGGUG.CAGU........AAUUUAC........AC..UGUAAGA.UUUGUU.UUCGGGCGCUUAUUUAUU
env-199   UUCACUCU.UCACCG.GAUGA...G.U.GG...........UUAAAGCGUUUGGGAGCA.AA.UCGUAGGUG.CAGU........AAUUUAC........AC..UGUAAGA.UUUGUU.UCCGGGCGCUUAUUUAUU
env-200   UUCACUCU.UCACCG.GAUGA...G.U.GG...........UUAAAGCGUCUGGGAGCA.AA.UCGUAGGUG.CAGU........AAUUUAC........AC..UGUAAGA.UUUGUU.UCUGGGCGCUUAUUUAUU
env-201   UUCACCCU.UCACCG.GAUGA...G.U.GG...........UUAAAGCGUCUGGGAGCA.AA.UCGUAGGUG.CAGU........AAUUUAC........AC..UGUAAGA.UUUGUU.UCCGGGCGCUUAUUUAUU
Ere-1-1   UUCACUCU.UCACCG.GAUGA...G.U.GG...........UUAAAGCGUCUGGGAGCA.AA.UCGUAGGUG.CAGU........AAUUUAC........AC..UGUAAGA.UUUGUU.UCCGGGCGCUUAUUUAUU
env-202   UUCACUCU.UCACCG.GAUGA...G.U.GG...........UUAAAGCGUCUGGGAGCA.AA.UCGUAGGUG.CAGU........AAUUUAC........AC..UGUAAGA.UUUGUUUUCCGGGCGUUUAUUUAUU
env-203   UUCACUCU.UCACCG.GAUGA...G.U.GG...........UUAAAGCGUCUGGGAGCA..A.UCGUAGGUG.CAGU........AAUUUAC........AC..UGUAAGA.UUUGUU.UCCGGGCGCUUAUUUAUU
env-204   UUCACUCU.UCACCG.GAUGA...G.U.GG...........UUAAAGCGUCUGGGAGCA.AA.UCGUAGGUG.CAGU........AAUUUAC........AC..UGUAAGA.UUUGUC.UCCGGGCGCUUAUUUAUU
env-205   UUCACUCU.UCACCG.GAUGA...G.U.GG...........UUAAAGCGUCUAGGAUCA.AA.UCGUAGGUG.CAGUG.......AUUU..........CAC..UGUAAGA.UAUGGU.CUUGGGCGCUUCUUUUUU
env-206   UUCACUCU.UCACCG.GAUGA...G.U.GG...........UUAAAGCGUCUAGAAUCA.AA.UCGUAGGUG.CAGUG.......AUUUA.........CAC..UGUAAGA.UUUGGU.UUUAGGCGCUUCUUUUUU
env-207   UUCACUCU.UCACCG.GAUGA...G.U.GG...........UUAAAGCGUCUAGGGUCA.AA.UCGUAGGUG.CAGUG.......AUUU..........CAC..UGUAAGA.UUUGGU.CUUAGGCGCUUCUUUUUU
env-208   UUCACUCU.UCACCG.GAUGA...G.U.GG...........UUAAAGCAUCUAGGAUCA.AA.UCGUAGGUG.CAGUG.......AUUU..........CAC..UGUAAGA.UUUGAU.CUUAGGCGUUUCUUUUUU
env-209   UUCACUCU.UCACCG.GAUGA...G.U.GG...........UUAAAGCGUCUAGGAUCA.AA.UCGUAGGUG.CAGUG.......ACUU..........CAC..UGUAAGA.UUUGGU.CUUAGGCGCUUCUUUUUU
env-210   CUCACUCU.UCACCG.GAUGA...G.U.GG...........UUAAAGCGUCUAGGAUCA.AA.UCGUAGGUG.CAGUG.......AUUU..........CAC..UGUAAGA.UUUGGU.CUUAGGCGCUUCUUUUUU
env-211   UUCACUCU.UCACCG.GAUGA...G.U.GG...........UUAAAGCGUCUAGGAUCA.AA.UCGUAGGUG.CAGUG.......AUUU..........CAC..UGUAAGA.UUUGGU.CUUAGACGCUUCUUUUUU
env-212   UUCACUCU.UCACCG.GAUGA...G.U.GG...........UUAAAGCGUCUAGGAUCA.AA.UCGUAGGUG.CAGUG.......AUUU..........CAC..UGUAAGA.UUUGAU.CUUAGGCGUUUCUUUUUU
env-213   UUCACUCU.UCACCG.GAUGA...G.U.GG...........UUAAAGCGUCUAGAAUCA.AA.UCGUAGGUG.CAGUG.......AUUU..........CAC..UGUAAGA.UUUGGU.CUUAGGCGCUUCUUUUUU
Ebi-1-1   UUCACUCU.UCACCG.GAUGA...G.U.GG...........UUAAAGCGUCUAGGAUCA.AA.UCGUAGGUG.CAGUG.......AUUU..........CAC..UGUAAGA.UUUGGU.CUUAGGCGCUUCUUUUUU
env-214   UUCACUCU.UCACCG.GAUGG...G.U.GG...........UUAAAGCGUCUAGGAUCA.AA.UCGUAGGUG.CAGUG.......AUUU..........CAC..UGUAAGA.UUUGGU.CUUAGGCGCUUCUUUUUU
env-215   GUCACUCU.UCACCG.GAUGA...G.U.GG...........UUAAAGCGUCUAGAAUCA.AA.UCGUAGGUG.CAGUGA......UU...........UCAC..UGUAAGA.UUUGGU.CUUAGGCGCUUCUUUUUU
env-216   UUCACUCU.UCACCG.GAUAA...G.U.GG...........UUAAAGCGUCUAGGAUCA.AA.UCGUAGGUG.CAGUGA......UU...........UCAC..UGUAAGA.UUUGGU.CUUGGGCGCUUCUUUUUU
env-217   UUCACUCU.UCACCG.GAUGA...G.U.GG...........UUAAAGCGUCUAGGAUCA.AA.UCGUAGGUG.CAGUGA......UU...........UCAC..UGUAAGA.UUUGGC.UUUAGGCGCUUCUUUUUU
env-218   UUCACUCA.UCACCG.GAUGA...G.U.GG...........GGAAGGCGUCUGGGAUUA.AA.UCGUAGGUG.CAGUGA......UGA..........UCAC..UGUAAGA.UUUGAU.CCUGGGCGCCUAUUUUUG
Osp-1-1   UUCACUCG.UCACCG.GAUGA...G.U.GA........UUUAUAAAGCAUCUGGAACUU.AA.UCGUAGGUA.CGGUGU......UCAC.........ACAC..CAUAAGA.UUAGGU.UCUGGAUGCUUUUUUGUU
env-219   UUCACUCA.UCACCG.GAUGG...G.U.GG.........UUAUAAGGCAUUCAAAGCUU.AA.UCGUAGGUA.CGGCA.......UUUU..........UGC..UAUAAGA.UUUUGC.UUUGGAUGCCUUUUGCUU
env-220   UUCACUCA.UCACCG.GAUGA...G.U.GG.........UCAAAAGGCAUUCAAAGCAU.AA.UCGUAGGUA.CGGCA.......UUUU..........UGC..UAUAAGA.UUUUGC.UUUGGAUGCCUUUUGCAU
env-221   UUCACUCA.UCACCG.GAUGG...G.U.GG.........UCAAAAGGCAUUCAAAGCAU.AA.UCGUAGGUA.CGGCA.......UUUU..........UGC..UAUAAGA.UUUUGC.UUUGGAUGCCUUUUGCAU
env-222   UUCACUCA.UCACUG.GAUGA...G.U.GG.........CAAUAAGGCAUUCAAAGCAU.AA.UCGUAGGUA.CGGCA.......UUUU..........UGC..CAUAAGA.UUUUGC.UUUGGAUGCUUUUUGUUU
env-223   UUCAUUCA.UCACCG.GAUGA...G.U.GG.........UAAUAAAGCAUUCAAGACUA.AA.UCGUAGGUA.CGGUGU......UUCG.........GCAU..UAUAAGA.UUUAGU.GUUGAAUGCUUAUUUUUU
env-224   UUCACUCA.UCACUG.GAUGA...G.U.GG.........CAAUAAAGCAUUCAAGACUA.AA.UCGUAGGUA.CGGUGU......UUCG.........GCAU..UAUAAGA.UUUAGU.UUUGAAUGCUUAUUUUUU
env-225   UUCACUCA.UCACCG.GAUGA...G.U.GG.........UAAUAAAGCAUUCAAGACUA.AA.UCGUAGGUA.CGGUGU......UUCG.........GCAU..UAUAAGA.UUUAGU.UUUGAGUGCUUAUUUUUU
env-226   UUCACUCA.UCACCG.GAUGA...G.U.GG.........UAAUAAAGCAUUCAAGACUA.AA.UCGUAGGUA.CGGUGU......UCCG.........GCAU..UAUAAGA.UUUAGU.UUUGAAUGCUUAUUUUUU
env-227   UUCACUCA.UCACCG.GAUGA...G.U.GG.........UAAUAAAGCAUUCAAGACUA.AA.UCGUAGGUA.CGGUGU......UUCG.........GCAU..UAUAAGA.UUUAGU.UUUGAAUGCUU.......
env-228   UUCACUCA.UCACCG.GAUGA...G.U.GG.........UAAUAAAGCAUUCAAGACUA.AA.UCGUAGGUA.UGGUGU......UUCG.........GCAU..UAUAAGA.UUUAGU.UUUGAAUGCUUAUUUUUU
env-229   UUCACUCA.UCACCG.GAUGA...G.U.GG.........UAAUAAAGCAUUCAAGACUA.AA.UCGUAGGUA.CGGUGU......UUCG.........GCAU..UAUAAGA.UUUAGU.UUUGAAUCAAGAAAAAGU
env-230   UUCACUCA.UCACCG.GAUGA...G.U.GA........UUACAAAGGCAUCCAAGAUUA.AA.UCGUAGGUA.CGGUGU......GUAU.........GCAC..UAUAAGA.UUUGAU.UUUGGGUGUUUUUUGUGU
env-231   UUCACUCG.UCACCG.GAUGA...G.U.GG.........UUAUAAGGCAUCCAAGAUUA.AA.UCGUAGGUA.CGGUGU......UUCG.........ACAC..UAUAAGA.UUUAGU.CUUGGGUGCCUAUUUUGU
Smo-1-1   UUCGCUCA.UCACCG.GAUGA...G.U.GG.........UAAUAAGGCAACCAAGAUUA.AA.UCGUAGGUA.CGGUGU......UUCG.........ACAC..UAUAAGA.UUUAGU.CUUGGUUGCUUAUUUUGU
env-232   UUCACUCA.UCACCG.GAUGA...G.U.GA.........UUAUAAGGCAUCCAAGAUUA.AA.UCGUAGGUA.CGGUGU......GUAU.........ACAC..UAUAAGA.UUUAGU.CUUGGAUGCCUUUUGUGU
env-233   UUCACUCA.UCACCG.GAUGA...G.U.GG........UUAUAAAGGCAUCCAAGACUU.AA.UCGUAGGUA.CGGUGU......UUCG.........ACAC..UAUAAGA.UUUAGU.CUUGGAUGCCUUUUCUUA
Csp-1-1   UUCACUCA.UCACCG.GGUGA...G.U.GA.........UUAUAAGGCAUCCAAGACUU.AA.UCGUAGGUA.CGGUGU......UUCG.........ACAC..UAUAAGA.UUUAGU.CUUGGAUGCCUAUUUUGU
[ae-1-1   UUCACUCA.UCACCG.GAUGA...G.U.GA.........CUAUAAGGCAUCCAAGACUU.AA.UCGUAGGUA.CGGUGU......UUCG.........ACAC..UAUAAGA.UUUAGU.CUUGGAUGCCUAUUUUUU
env-234   UUCACUCA.UCACCG.GAUGA...G.U.GA.........CUAUAAGGCAUCCAAGACUU.AA.UCGUAGGUA.CGGUGU......UUCG.........ACAC..UAUAAGA.UUUCGU.CUUGGAUGCCUAUUUUGU
Cpa-1-1   UUCACUCA.UUACCG.GAUGA...G.U.GA.........CUAUAAGGCAUUCAAGACUU.AA.UCGUAGGUA.CGGUGU......UUCG.........ACAC..UAUAAGA.UUUAGU.UUUGGAUGCCUAUUUUGU
Lba-2-1   UGCAUUCU.UCACCG.GAAGA...A.G.CA..........UAUAAGGCAUUCAAAAACA.GA.CCGCAGGUA.CGGUGUGG....CUUU.......CCACAC..UAUAAGG.UUUGUU.UUUGAAUGCCUUUUGUAU
env-235   AU.CCUCA.UCACCG.GAUGA...A.G.AU........AUGUUUAAGCAUCCAAAAGUA.AA.CCGUAGGUA.CGGUGUG.....AUUU........CACAC..UAUAAGG.UUUGCU.UUUGGGUGCUUUUUGUUU
env-236   GC.UUUCU.UCACCG.GAUGA...A.A.GC.........AUAUAAGGCAUUCAGGAAUA.AA.CCGCAGGUA.CGGUGUGA....UUU........UCACAC..UAUAAGG.UUUGUU.CCUGGGUGCUUUUUGUUU
env-237   GC.UUUCU.UCACCG.GAUGA...A.G.GC.........AUAUAAGGCAUUCAGGAAUA.AA.CCGCAGGUA.CGGUGUGA....UUU........UCACAC..UAUAAGG.UUUGUU.CCUGGGUGCUUUUUGUUU
env-238   GC.UUUCU.UCACCG.GAUGA...A.A.GC.........AUAUAAGGCAUCCAAAGGCA.AA.CCGCAGGUA.CGGUGUGA....UUU........UCACAU..CAUAAGG.UUUGAC.UUUGGAUGCCUUUUGUUU
env-239   GC.UGUUA.UCACCG.GAUAA...U.A.GC.........AUUAUAGGCAUCCAAAAGCC.AA.CCGUAGGUA.CGGUGUG.....UUUU........CACAC..UAUAAGG.UCAGCU.UUUGGGUGCCUUUUUAUU
Cbe-2-1   AC.UUUCA.UCACCG.GAUGA...U.U.GU........AUUAUAAGGCAUACAAAAGCU.AG.CCGUAAGUA.CGGUGUG.....UUUU........CACAC..UAUAAGG.UUAGCU.UUUGGAUGCCUUUUGUUU
Cbe-1-1   AC.UUUCA.UCACCG.GAUGA...U.U.GU........AUUAUAAGGCAUACAAAAGCU.AG.CCGUAGGUA.CGGUGUG.....UUUU........CACAC..UAUAAGG.UUAGCU.UUUGGAUGCCUUUUGUUU
Csp-2-1   AC.UUUCA.UCACCG.GAUGA...U.U.GU........AUUAUAAGGCAUACAAAAGCC.AG.CCGUAGGUA.CGGUGUG.....UUUU........CACAC..UAUAAGG.UUAGCU.UUUGGAUGCCUUUUGUUU
env-240   GC.UUUCA.UCACCG.GAUGA...U.U.GC........AUUAUAAGGCAUCCAAAAGCC.AG.CCGUAGGUA.CGGUGUG.....UUUU........CACAC..UAUAAGG.UUAGCU.UUUGGGUGCUUUUUGUUU
Bxy-1-1   GC.UUUCA.UCACCG.GAUGA...U.U.GC........AUUAUAAGGCAUCCAAAAGCC.AA.CCGUAGGUA.CGGUGUG.....UUAU........CACAC..UAUAAGG.UUAGCU.UUUGGGUGUCUUUUAUUU
env-241   GC.UUUCA.UCACCG.GAUGA...U.U.GC........AUUAUAAGGCAUCCAAAAGCC.AA.CCGUAGGUA.CGGUGUG.....UUUU........CACAC..UAUAAGG.UUAGCU.UUUGGGUGCCUUUUGUUA
env-242   GC.UUUCA.UCACCG.GAUGA...U.U.GC........AUUAUAAGGCAUCCAAAAGCC.AA.CCGUAGGUA.CGGUGUG.....UUUU........CACAC..CAUAAGG.UUAGCU.UUUGGAUGCCUUUUGUUA
Csa-2-1   GC.UUUCA.UCACCG.GAUGA...U.U.GC........AUUAUAAGGCAUCCAAAAGCC.AA.CCGUAGGUA.CGGUGUG.....UUUU........CACAC..UAUAAGG.UUAGCU.UUCGGAUGCCUUUUGUUU
Csa-1-1   UGCUUUCA.UCACCG.GGUGA...U.U.GU........AUUAUAAGGCAUCCAAAAGCC.AA.CCGUAGGUA.CGGUGUG.....UUUU........CACAC..UAUAAGG.UUAGCU.UUUGGGUGCCUUUUAUUU
env-243   UGCCAUCA.UCACCG.GAUGA...UUG.CA.........UUAUAAGGCAUCCAAAAGCC.AA.CCGUAGGUA.CGGUGUG.....UUAU........CACAC..UAUAAGG.UUAGCU.UUUGGGUGUCUUUUAUUU
env-244   UGCCUUCA.CCACCG.GGUGA...UUG.CA.........UUAUAAGGCAUCCAAAAGCC.AG.CCGUAGGUA.CGGUGUG.....UUUU........CACAC..CAUAAGG.UUAGCU.UUUGGAUGUCUUUUUUUA
env-245   UUCUUUUA.UCACCG.GAUAA...A.A.GG..........UAUCAGGCAUCCAGGACUA.AA.CCGUAGGUA.CGGUGCAGC...UAU.......GCUGCAU..UAUAAGG.UUUGGU.CUUGGGUGCCUGUUUUUU
env-246   AU.UUUUA.UCACCG.GAUAA...A.A.AU........AGUUGAAAGCGUCUGAAAUCA.UA.CCGUAGGUA.CGGCGUAAC...UUAU......GUUGCGU..UAUAAGG.UUUGGU.UUUGGAGGCUUAUUUUUU
env-247   AU.UUUUG.UCACCG.GAUAA...A.A.AU........AGUUGAAAGCGUCUGAAAUCA.UA.CCGUAGGUA.CGGCGUAAC...UUGU......GUUGCGU..UAUAAGG.UUUGGU.UUUGGAGGCUUAUUUUUU

          ............<<<.....................................................>>>..................................................................
          .........................................................................................................................................
          <<.<<<<<.<<.....>>>>>...>.>.>>.............<<<<<<<<<<<<<<<<.<<.<<..........<<<<<<<<<<.......>>>>>>>>>>.......>>.>>>>>>.>>>>>>>>>>>>......
          22.22222.21.....12222...2.2.22..............221222222222222.22.2...........2222221110.......0111222222........2.222222.222222222122......
          YY∘YYYYR-YCACCG-GRURR---R-R-R•----------∘••RRRG•RYYCRRRRYY•-•R-CCGUAGGUA-YRGCR•∘-----•••∘---------∘•YGY--YAUAAGG-Y••R•Y-YYYGRRY•CYUUUUYYYY
```

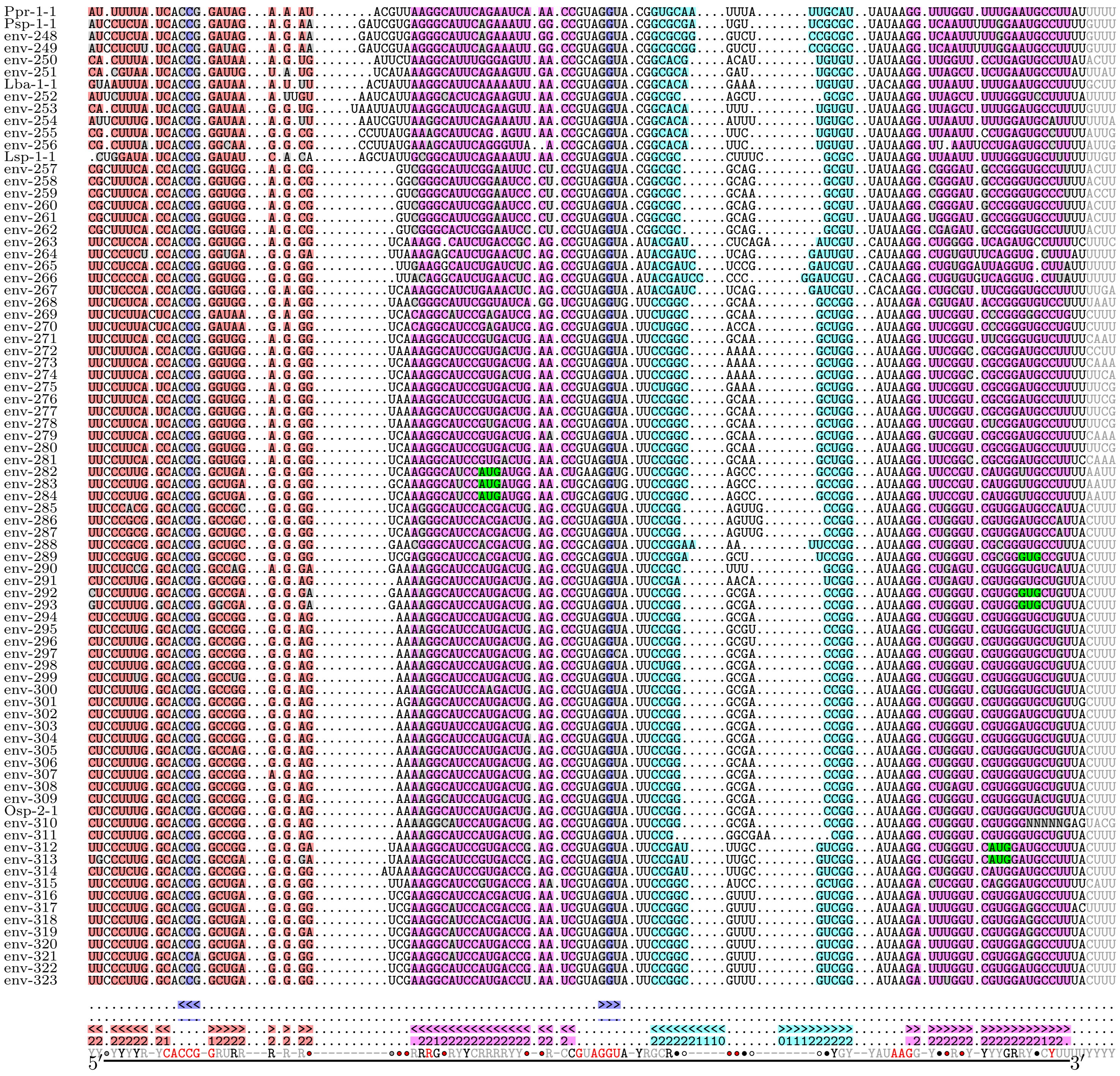
Ppr-1-1
Psp-1-1
env-248
env-249
env-250
env-251
Lba-1-1
env-252
env-253
env-254
env-255
env-256
Lsp-1-1
env-257
env-258
env-259
env-260
env-261
env-262
env-263
env-264
env-265
env-266
env-267
env-268
env-269
env-270
env-271
env-272
env-273
env-274
env-275
env-276
env-277
env-278
env-279
env-280
env-281
env-282
env-283
env-284
env-285
env-286
env-287
env-288
env-289
env-290
env-291
env-292
env-293
env-294
env-295
env-296
env-297
env-298
env-299
env-300
env-301
env-302
env-303
env-304
env-305
env-306
env-307
env-308
env-309
Osp-2-1
env-310
env-311
env-312
env-313
env-314
env-315
env-316
env-317
env-318
env-319
env-320
env-321
env-322
env-323
5′
3′

```
env-324   UUCCCUUG.GCACCG.GCUGA...G.G.GG..........UCAAAGGCAUUCGUGACCG.AA.UCGUAGGUG.UUCCGGC.....AUUU........GCUGG...AUAAGA.UUUGGU.CAUGAAUGUCUUUACUUU
env-325   UUCCCUUG.GCACCG.GCUGA...G.G.GG..........UCAAAGGCAUCCGUGACCA.AA.UCGUAGGUA.UUCCGGC.....AUUU........GCCGG...AUAAGA.UUUGGC.CAUGGAUGUCUUUACAUU
env-326   UUCCCUUG.GCACCG.GCUGA...G.G.GG.........CUCACAGGCAUCUGUGACCG.AA.UCGUAGGUA.UUCCGGC.....AUCU........GCUGG...AUAAGA.UUUGGC.CAUGGAUGCCUAUAUUUU
env-327   UUCCCUUG.GCACCG.GCUGA...G.G.GG.........CUCACAGGUAUCCGUGACCG.AA.UCGUAGGUA.UUCCGGC.....AUCU........GCUGG...AUAAGA.UUUGGU.CAUGGAUGCCUGUAUUUU
env-328   UUCCCUUG.GCACCG.GCUGA...G.G.GG.........CUCACAGGCAUCCGUGACCG.AA.UCGUAGGUA.UUCCGGC.....AUCU........GCUGG...AUAAGA.UUUGGU.CAUGGAUGCCUAUAUUUU
env-329   UUCCCUUG.GCACCG.GCUGA...G.G.GG.........CUCACAGGCAUCCGUGACCG.AA.UCGUAGGUA.UUCCGGC.....AUCU........GCUGG...AUAAGA.UUUGGC.CAUGGAUGCCUAUAUUUU
env-330   UUCCCUUG.GCACCG.GCUGA...G.G.GG.........CUCACAGGCAUCCGUGACCG.AA.UCGUAGGUA.UUCCGGC.....AUCU........GCUGG...AUAAGA.UUUGGA.CAUGGAUGCCUAUAUUUU
env-331   UUCCCUUG.GCACCG.GCUGA...G.G.GG..........UCAAAGGCAUCCAUGACCG.AA.UCGUAGGUA.UUCCGGC.....GUUU........GCUGG...AUAAGA.UUUGGU.CAUGGAUGUCUUUACUUU
env-332   UUCCCUUG.GCACCG.GCUGA...G.G.GG..........UCAAAGGCAUUCGUGACCG.AA.UCGUAGGUG.UUCCAGCA....UU.........UGCUGG...AUAAGA.UUUGGU.CAUGGAUGUCUUUAUUUU
env-333   UUCCCUUG.GCACCG.GCUGA...G.G.GG..........UCAAAGGCAUUCGUGACCG.AA.UCGUAGGUG.UUCCGGCA....UU.........UGCUGG...AUAAGA.UUUGGU.CAUGGAUGUCUUUAUUUU
env-334   UUCCCUUG.GCACCG.GCUGA...G.G.GG..........UCAAAGGCAUUCGUGACCG.AA.UCGUAGGUA.UUCCGGCA....UU.........UGCUGG...AUAAGA.UUUGGU.CAUGGAUGUCUUUACUUU
env-335   UUCCCUUG.GCACCG.GCUGA...G.G.GG..........UCAAAGGCAUCCAUGACCG.AA.UCGUAGGUA.UUCCGGCA....UU.........UGCUGG...AUAAGA.UUUGGU.CAUGGAUGCCUUUACUUU
env-336   UUCCCUUG.GCACCG.GCCGA...G.G.GG.........UCAAAAGGCAUCCGUGACCG.AA.UCGUAGGUA.UUCCGGC.....AUCU........GCUGG...AUAAGA.UUUUGU.CGUGGAUGUCUUUACUUU
env-337   UUCCCUUG.GCACCG.GCCGA...G.G.GG..........UGAAAGGCAUCCGUGACCA.GA.UCGUAGGUA.UUCCGGCA....UU.........UGCCGG...AUAAGA.UCAGGU.CAUGGACGCCUUUACUUU
env-338   UUCCCCCG.GCACCG.GCUGG...G.G.GG...........CAAGGGCAUCCAUGACCG.AA.CCGUAGGUA.UUCCGGC.....GUCU........GCCGG...AUAAGG.UUCCGU.CGUGGAUGCCCGUAUUUU
env-339   CUCCCCCG.UCACCG.GAUGG...G.G.GG..........CCGAAGGCAUCCAGGAUCG.GA.ACGCAGGUA.UUCCAGC.....CCCC........GCUGG...AUAAGU.UCCGGU.CCCGGGUGCCCAUUCUUU
env-340   UGCCCCUG.UCACCG.GAUGG...G.G.GA..........GAACAGGCAUCCAGGAUCG.GG.ACGUAGGUA.UUCCAGC.....ACAC........GCUGG...AUAAGC.CCCGGU.CUUGGGUGCCUGUAACUU
env-341   UGCCCCUG.UCACCG.GAUGG...G.G.GA..........GAACAGGCAUCCAGGAUCG.GG.ACGUAGGUA.UUCCAGC.....GCAC........GCUGG...AUAAGC.CCCGGU.CUUGGGUGUCUGUAACUU
env-342   CUCCCCCG.UCACCG.GAUGG...G.G.GA...........UCAAGGUAUCCGGGACCG.GA.GCGUAGGUA.UUCCGACA....UU.........UGUCGG...AUAAGC.GCCGGU.CUUGGGUGCCUUUACUUU
env-343   CUCCCCCG.UCACCG.GAUGG...G.G.GG..........AUCAAGGUAUCCGGGACCG.GA.GCGUAGGUA.UUCCGACA....UU.........UGUCGG...AUAAGC.GCCGGU.CUUGGGUGCCUUUACUUU
env-344   CUCCCCCG.UCACCG.GCUGG...G.G.GG..........AUCAAGGUAUCCGGGACCG.GA.GCGUAGGUA.UUCCGACA....UU.........UGUCGG...AUAAGC.GCCGGU.CUUGGGUGCCUUUACUUU
env-345   CUCCCCCG.UCACCG.GAUGG...G.G.GG..........AUCAAGGUGUCCGGGACCG.GA.GCGUAGGUA.UUCCGACA....UU.........UGUCGG...AUAAGC.GCCGGU.CUUGGGUGCCUUUACUUU

          ............<<<.....................................................>>>..................................................................
          .........................................................................................................................................
          <<.<<<<<.<<.....>>>>>...>.>.>>.............<<<<<<<<<<<<<<<<.<<.<<..........<<<<<<<<<<.......>>>>>>>>>>.......>>.>>>>>>.>>>>>>>>>>>>......
          22.22222.21.....12222...2.2.22.............2212222222222222.22.2...........2222221110.......0111222222........2.222222.22222222122.......
          YY∘YYYYR-YCACCG-GRURR---R-R-R•----------∘••RRRG•RYYCRRRRYY•-•R-CCGUAGGUA-YRGCR•∘-----•••∘--------∘•YGY--YAUAAGG-Y••R•Y-YYYGRRY•CYUUUUYYYY
        5′                                                                                                                                       3′
```
